# Atmospheric pressure water–gas shift reaction and transfer hydrogenation of ketones using a ruthenium pincer catalyst

**DOI:** 10.1039/d6cy01293k

**Published:** 2026-09-25

**Authors:** James Luk, Luming Guo, Michael Bühl, Amit Kumar

**Affiliations:** a EaStCHEM, School of Chemistry, University of St Andrews North Haugh KY16 9ST St Andrews UK ak336@st-andrews.ac.uk

## Abstract

We report the use of a Ru-MACHO pincer complex as a catalyst for the homogeneous water–gas shift reaction (WGSR) under atmospheric pressure of CO and at 150 °C, achieving cumulative turnover numbers (TONs) of more than 6000 from the catalyst recycling of a total of 4 runs. This activity was then exploited to reduce ketones to secondary alcohols *via* transfer hydrogenation using CO and water as a reducing agent. Stoichiometric NMR experiments and DFT calculations were performed to elucidate key organometallic intermediates and the catalytic cycle.

## Abstract 2

A Ru-MACHO pincer complex catalyses the homogeneous water–gas shift reaction under 1 atm CO at 150 °C, achieving a cumulative TON >6000 over four catalyst-recycling runs.

## Graphical abstract

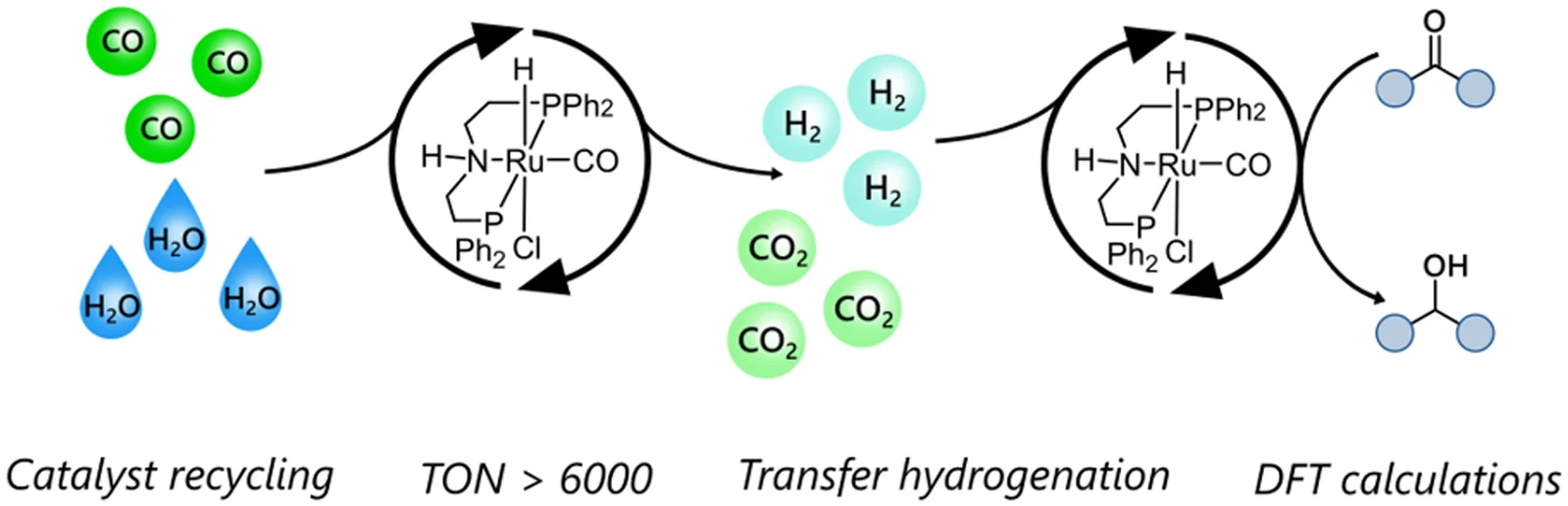

## Introduction

Hydrogen gas is increasingly important to modern chemical technology, with key applications in energy and atom-efficient chemical transformations, including petroleum refining (hydrocracking and desulfurisation), ammonia synthesis, and methanol production.^1^ As a result, global demand for H_2_ continues to rise, reaching 100 MT in 2024.^2^

The water–gas shift reaction (WGSR) plays a key role in industrial production of H_2_ by converting CO and H_2_O into CO_2_ and H_2_.^3^ In conventional steam methane reforming, the WGSR plays an important role in enhancing H_2_ output while reducing CO content. The industrial WGSR is carried out heterogeneously in two stages: a high-temperature stage (300–450 °C) that provides fast kinetics, and a low-temperature stage (200–250 °C) that improves H_2_ selectivity. Both steps also require different catalysts (for example, stage 1: Fe–Cr oxide and stage 2: Cu–Zn–Al).^4,5^

Homogeneous transition metal-catalysed systems have been used to achieve the WGSR at lower temperatures (65–150 °C). The earliest of these was reported by Ford *et al.* in 1977,^6^ who achieved the WGSR using Ru_3_(CO)_12_. Subsequently, several catalysts based on Rh,^7–9^ Ru,^9–11^ and Ir^12^ have been reported for the WGSR. However, many of these examples required moderate to high CO pressure (10–100 bar) to obtain satisfactory catalytic activity.^13^ While examples conducted at atmospheric pressure have been reported, these systems exhibited low catalytic TONs (<150).^6,13^ The required high pressure necessitates the use of specialised high-pressure equipment and infrastructure to conduct these reactions. Consequently, a catalytic system combining the atmospheric pressure with lower temperatures, while maintaining competitive activity, would be highly desirable from both safety and environmental perspectives. Other than hydrogen production, the WGS reaction has found application as a source of *in situ*-generated hydrogen for reductive transformations.^14^ Using water and CO as the hydrogen source for transfer hydrogenation would generate the product with CO_2_ as a by-product, which can be easily removed compared to the generation of stoichiometric waste formed from current transfer hydrogenation methods (*e.g.* acetone from *i*PrOH). Transfer hydrogenation using CO/water as a reductant has been demonstrated in the past^14^ for the reduction of nitroarenes,^15–18^ imines,^19^ alkenes,^20–24^ and alkynes.^25^ A few examples for the reduction of carbonyl bonds have also been reported; however, they typically require high CO pressure (10 bar).

We have recently reported the use of sequential amine formylation and decarbonylation to achieve the reverse water–gas shift (RWGS) reaction catalysed by a Ru-MACHO pincer complex.^26^ Here, we report that the Ru-MACHO pincer complex can also catalyse the water–gas shift (WGS) reaction. Furthermore, the water–gas shift reaction could be coupled with the hydrogenation reaction to demonstrate a one-pot transfer hydrogenation system where water serves as the hydrogen source.

## Results and discussion

Building on our previous studies of the reverse water–gas shift reaction,^26^ initial screening of the water–gas shift reaction was performed using precatalysts 1–4 (0.01 mmol), KO*t*Bu (0.04 mmol), and H_2_O (1 mL) in THF (1 mL), at 150 °C for 18 h and 1.5 bar pressure (2 : 1 Ar : CO). Under these conditions, 1 led to the formation of H_2_ and CO_2_ with a TON of 10 (entry 1), while precatalysts 2, 3 and 4 did not form any product (entries 2–4).

Evaluation of other solvents revealed a pronounced influence of the solvent on catalytic performance. Switching from THF to 2-MeTHF led to a significant enhancement, increasing the TON from 10 to 50 (entry 5). Further improvements were observed with 1,4-dioxane, *t*-BuOH and *t*-AmOH showing a TON of 180, 160, and 190, respectively (entries 6–8). Notably, toluene and anisole afforded substantially higher TONs of 460 and 1210, respectively (entries 9 and 10), whereas xylene delivered an even higher TON of 2570 (entry 11). We postulate that the large variation in TON due to a change in solvent is due to the competing solubilities or gas–liquid mass transfer of the reactants (H_2_O and CO), catalyst, and products (H_2_O and CO_2_) in the reaction medium.

The effect of water loading was examined next. Halving the water volume (0.5 mL) lowered the TON to 780 (entry 12), whereas doubling to 2 mL (entry 13) also decreased the activity (TON 1930), suggesting that the reaction benefits from an optimal water : solvent balance attributed to the varying solubility of substrates and products as stated previously. Similarly, changing the total xylene volume to 0.5 mL (entry 14) or 2 mL (entry 15) also decreased the catalytic efficiency.

The nature of the base also strongly influenced the catalytic activity. Changing the anion decreased the TON (KOH = 1470 and K_2_CO_3_ = 1250, entries 16 and 17, respectively), while changing the cation to Na^+^ or Li^+^ resulted in even lower TONs (340 and 370, entries 18 and 19, respectively). In the absence of a base (entry 20), the catalytic activity dropped significantly (TON = 2). Increasing the base loading to 0.1 mmol (entry 21) and 0.2 mmol (entry 22) resulted in reduced TONs of 1100 and 1060, respectively, compared with 0.04 mmol KO*t*Bu (entry 11). Notably, complex 5 (Ru-MACHO-BH), a form of 1 (Ru-MACHO) that does not require base activation, afforded a TON of 2160 in the absence of a base (entry 23), comparable to that achieved with 1 and 0.04 mmol KO*t*Bu (TON = 2570, entry 11). This result indicated that the negligible activity of 1 in the absence of a base reflects incomplete catalyst activation rather than an intrinsic requirement for a base during turnover. It is worth noting that all these reactions were performed using a magnetic stirrer at 550 rpm. Considering the possible effect of stirring on mass transfer and gas mixing, we performed two catalytic experiments, one at 100 rpm and the other at 850 rpm under the conditions described in Table 1 entry 11 (TON 2570). The reaction performed at 100 rpm showed a TON of 1438, whereas that at 850 rpm showed a TON of 2500. This suggests that 550 rpm is sufficient for the needed mass transfer under the reaction conditions. To facilitate catalyst and substrate interactions in a biphasic solvent, the optimised reaction (Table 1, entry 11) was repeated in the presence of a phase transfer catalyst, tetrabutylammonium bromide (0.05 mmol). Unfortunately, the reaction showed only a TON of 35, which is significantly lower than that without using the phase transfer catalyst (2570, entry 11). We speculate this could be due to the inhibition of ruthenium catalysis by the phase transfer catalyst.

**Table 1:** Optimisation of the WGS reaction^a^

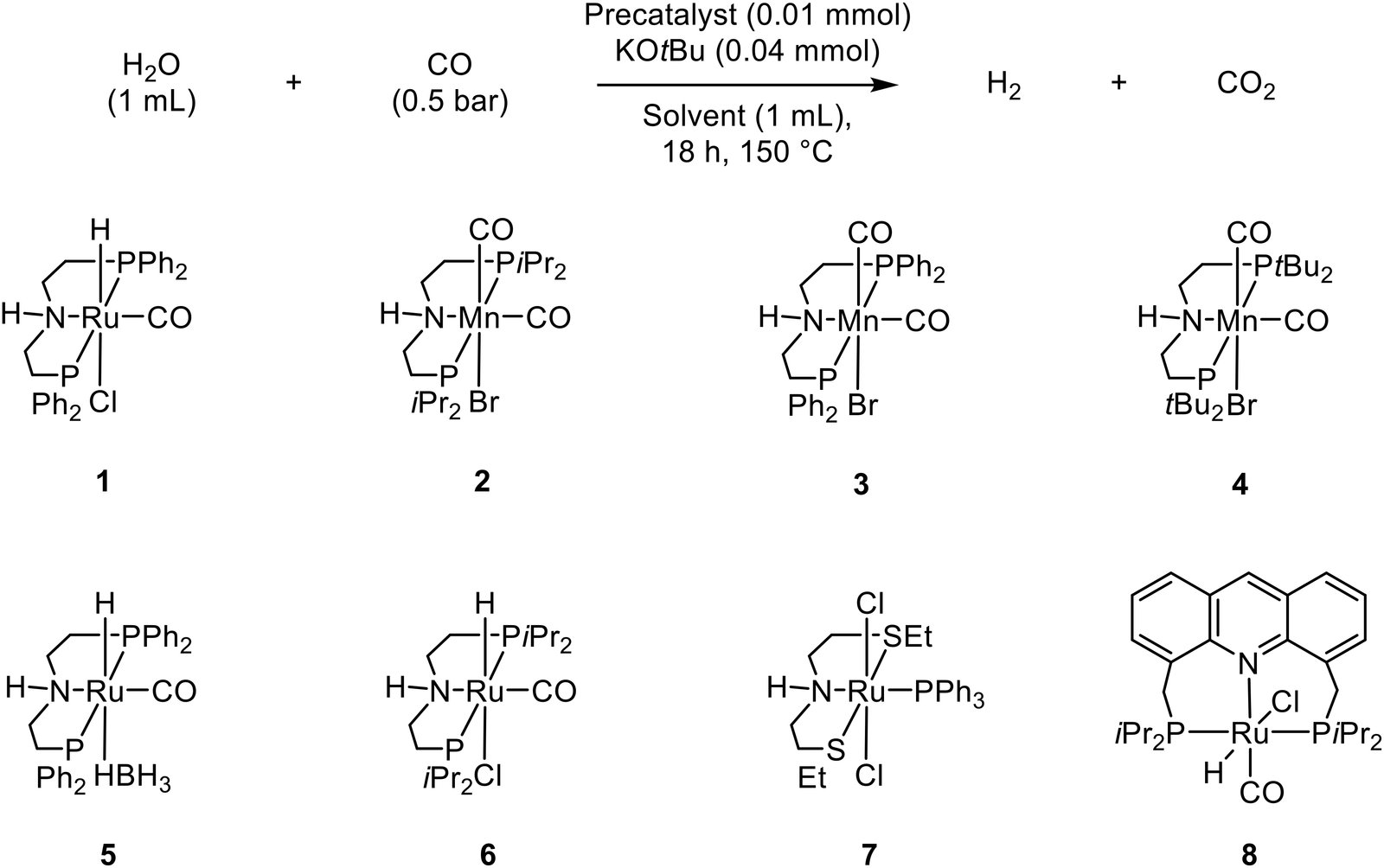					
Entry	Precatalyst (mmol)	Base (mmol)	H_2_O (mL)	Solvent (mL)	TON^b^
1	1 (0.01)	KO*t*Bu (0.04)	1	THF	10
2	2 (0.01)	KO*t*Bu (0.04)	1	THF	n.o.
3	3 (0.01)	KO*t*Bu (0.04)	1	THF	n.o.
4	4 (0.01)	KO*t*Bu (0.04)	1	THF	n.o.
5	1 (0.01)	KO*t*Bu (0.04)	1	2-MeTHF	50
6	1 (0.01)	KO*t*Bu (0.04)	1	1,4-Dioxane	180
7	1 (0.01)	KO*t*Bu (0.04)	1	*t*BuOH	160
8	1 (0.01)	KO*t*Bu (0.04)	1	*t*AmOH	190
9	1 (0.01)	KO*t*Bu (0.04)	1	Toluene	460
10	1 (0.01)	KO*t*Bu (0.04)	1	Anisole	1210
11	1 (0.01)	KO*t*Bu (0.04)	1	Xylene	2570
12	1 (0.01)	KO*t*Bu (0.04)	0.5	Xylene	780
13	1 (0.01)	KO*t*Bu (0.04)	2	Xylene	1930
14	1 (0.01)	KO*t*Bu (0.04)	1	Xylene (0.5)	800
15	1 (0.01)	KO*t*Bu (0.04)	1	Xylene (2)	1800
16	1 (0.01)	KOH (0.04)	1	Xylene	1470
17	1 (0.01)	K_2_CO_3_ (0.04)	1	Xylene	1250
18	1 (0.01)	NaO*t*Bu (0.04)	1	Xylene	340
19	1 (0.01)	LiO*t*Bu (0.04)	1	Xylene	370
20	1 (0.01)	—	1	Xylene	2
21	1 (0.01)	KO*t*Bu (0.10)	1	Xylene	1100
22	1 (0.01)	KO*t*Bu (0.20)	1	Xylene	1060
23	5 (0.01)	—	1	Xylene	2160
24	6 (0.01)	KO*t*Bu (0.04)	1	Xylene	6
25	7 (0.01)	KO*t*Bu (0.04)	1	Xylene	<1
26	8 (0.01)	KO*t*Bu (0.04)	1	Xylene	<1
27	2 (0.01)	KO*t*Bu (0.04)	1	Xylene	<1
28	3 (0.01)	KO*t*Bu (0.04)	1	Xylene	<1
29	4 (0.01)	KO*t*Bu (0.04)	1	Xylene	<1

a Conditions: precatalyst (0.01 mmol), KO*t*Bu (0.04 mmol), H_2_O (1 mL), xylene (1 mL), 150 °C, 18 h, 1.5 bar (1 : 2 CO : Ar).

b TONs determined by GC-TCD analysis of headspace ((total mmol of CO_2_ and H_2_ formed/2)/(mmol of catalyst used)). The experiments were conducted in a 250 mL J-Young's flask using a magnetic stirrer at 550 rpm. The TONs have been rounded to the nearest 10 in most cases.

Under these optimised conditions, the choice of the precatalyst was reevaluated. Complexes 6–8 (entries 24–26) and 2–4 (entries 27–29) exhibited significantly lower TONs of ≤6, confirming that 1 (Ru-MACHO) and 5 (Ru-MACHO-BH) are the most suitable catalysts for this transformation.

Having established conditions for a high TON, we then sought to improve the yield by shifting the equilibrium toward the products by removing CO_2_ as it was formed. A dual-chamber “H-flask” design was tested (Fig. 1): one chamber (chamber A) contained the catalyst (1, KO*t*Bu), water, and solvent (xylene), while the second (chamber B) held an aqueous ethanolamine solution (30 wt%) to capture CO_2_ as it formed. An atmospheric pressure of CO was introduced to the flask for the water gas shift reaction. Chamber A was kept at the reaction temperature, whereas chamber B was kept at room temperature. At 150 °C, solvent transfer occurred between chambers, affording a lower yield of H_2_ (39%, Table 2, entry 1). However, lowering the temperature to 135 °C prevented solvent migration and enabled quantitative removal of CO_2_, leaving H_2_ (95% yield) and unreacted CO as the only headspace gases (confirmed by GC-TCD), demonstrating that continuous removal of CO_2_ shifts the WGS equilibrium towards complete H_2_ formation even under milder conditions (Table 2, entry 2). Indeed, ^1^H NMR analysis of the solution present in chamber B revealed the formation of the corresponding ethanolamine carbamic acid formed from the reaction of aqueous ethanolamine with CO_2_. Reducing the temperature reduced the yield to 48%, suggesting that 135 °C is the optimum temperature for this setup (Table 2, entry 3). By reducing the loading of 1 to 0.001 mmol, a higher TON of 1000 was achieved; however, the conversion of CO and the yield of H_2_ were found to be lower in this case (Table 2, entry 4).

**Fig. 1:**
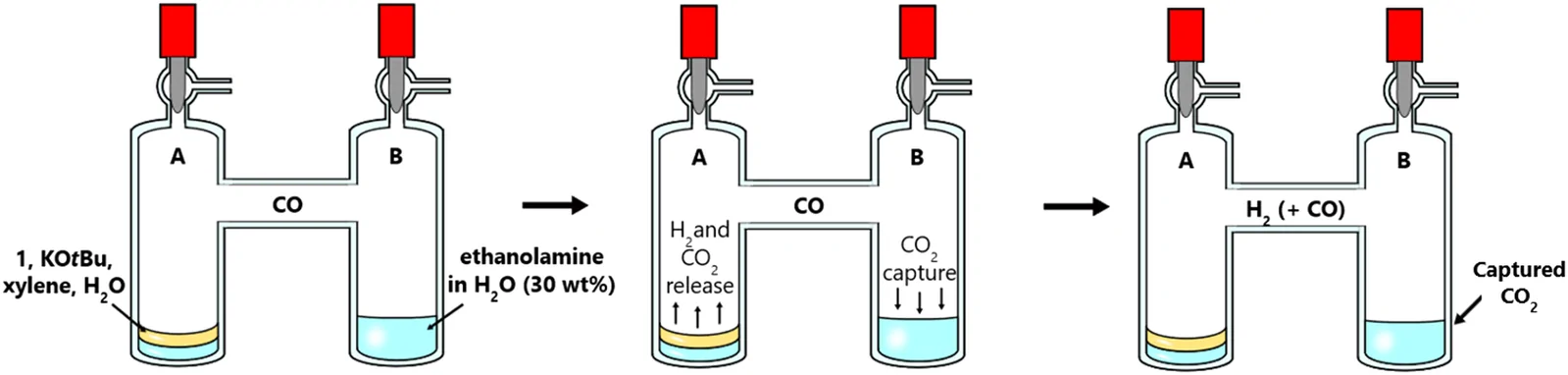
Schematic of H-flask reactions with chamber A containing 1 (0.01 mmol), KO*t*Bu (0.04 mmol), xylene (1 mL), and water (1 mL), and chamber B containing ethanolamine in H_2_O (30 wt%, 2 mL, RT) and shared headspace.

**Table 2:** H-flask reactions, with CO_2_ capture for pure H_2_ formation^a^

Entry	1 (mmol)	*T* (°C)	TON^b^	Yield_H_2__^c^ (%)
1	0.01	150	180	39
2	0.01	135	420	95
3	0.01	110	230	48
4	0.001	135	1000	23

a Conditions: chamber A: 1 (0.001–0.01 mmol), KO*t*Bu (0.04 mmol), H_2_O (1 mL), xylene (1 mL), 110–150 °C, 18 h. Chamber B: ethanolamine in H_2_O (30 wt%, 2 mL), room temperature. All under 1 bar CO.

b TONs determined by GC-TCD analysis of headspace ((mmol of H_2_ formed)/(mmol of catalyst used)).

c Yield of H_2_ determined with respect to CO as the limiting reagent. The TONs have been rounded to the nearest 10.

As complex 1 is also known to hydrogenate ketones at low pressures of H_2_,^27^ the use of this system was investigated for transfer hydrogenation of ketones. Under these conditions, benzophenone was hydrogenated to diphenylmethanol in 64% yield (Table 3, entry 1) in a 250 mL J-Young's flask. Reducing the size of the flask to 100 mL increased the product yield to 95%, likely by increasing hydrogen pressure. Under these conditions, a series of ketones was studied for transfer hydrogenation. It was found that aromatic ketones (entries 2–5) afforded higher yields, whereas aliphatic substrates (entries 6 and 7) gave significantly lower conversions. Compared with a previous report^27^ on the transfer hydrogenation of ketones, although using methanol–water as a hydrogen source, our system demonstrated lower turnover numbers, presumably due to CO inhibition. Benzophenone imine was also tested as an example of the transfer hydrogenation of imine substrates under these conditions; however, this only resulted in the formation of the hydrogenation product, diphenylmethanamine, in ∼20% yield.

**Table 3:** Transfer hydrogenation of ketones^a^

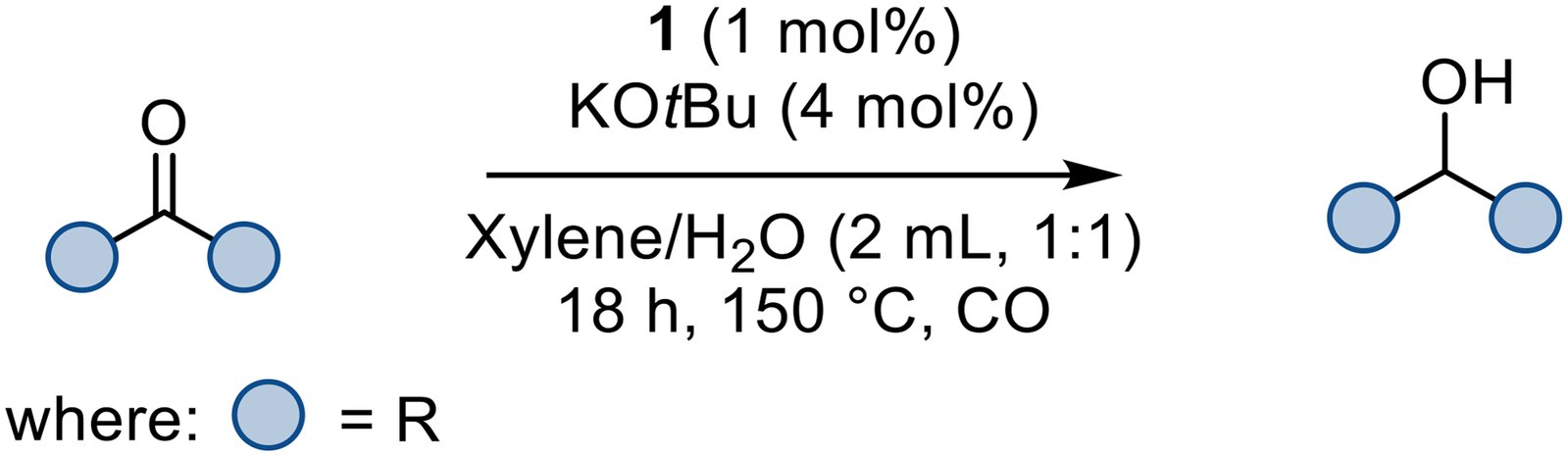		
Entry	Ketone	Yield (%)^b^
1^c^	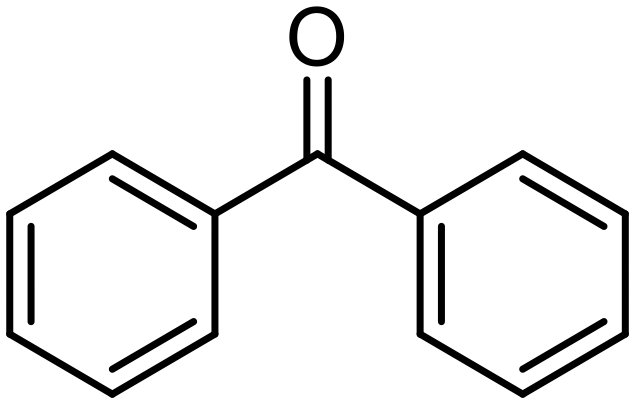	64
2	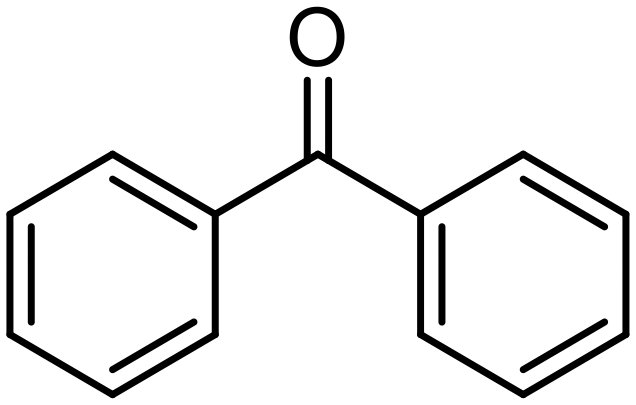	95
3	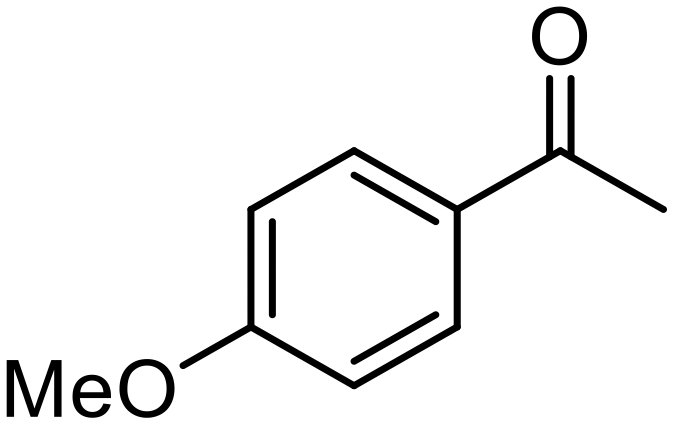	60
4	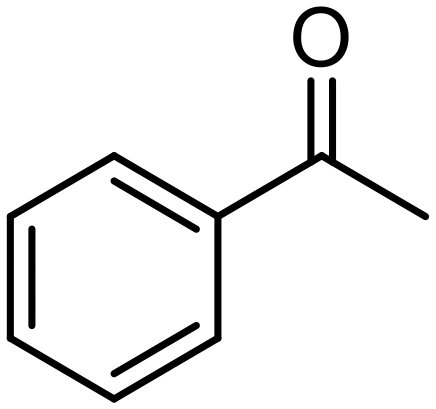	87
5	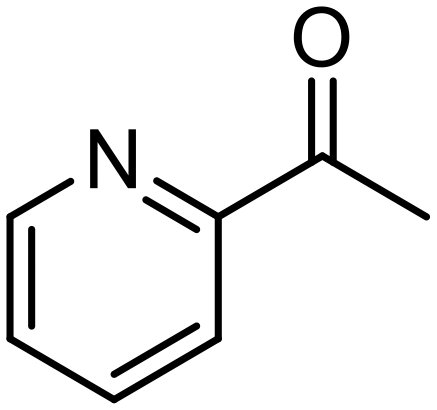	80
6	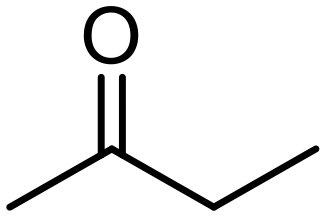	48
7	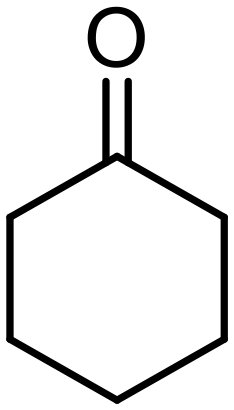	15

a Conditions: 1 (0.01 mmol), KO*t*Bu (0.04 mmol), H_2_O (1 mL), xylene (1 mL), 150 °C, 18 h, 1.5 bar (1 : 2 CO : Ar), in a 100 mL J-Young's flask.

b Yield determined by reference to the internal standard by ^1^H NMR spectroscopy.

c 250 mL J-Young's flask was used.

To gain insights into the organometallic species present during the catalytic transformation, we conducted a reaction in a J-Young's NMR tube and monitored it by NMR spectroscopy. Upon addition of KO*t*Bu (0.02 mmol) and H_2_O (0.1 mL) to a solution of 1 (0.02 mmol) in toluene-*d*_8_ (5 h, 110 °C), the disappearance of the signal corresponding to precatalyst 1 (^1^H NMR: *δ* −14.89 ppm, ^31^P{^1^H} NMR: *δ* 53.3 ppm) was observed alongside the emergence of new signals in NMR spectra (^1^H NMR: *δ* −14.96 ppm, ^31^P{^1^H} NMR: *δ* 53.1 ppm). This species was tentatively proposed to be the hydroxide complex 1A–**OH** (Fig. 3) based on previously reported RuMACHO-hydroxide complexes containing *i*Pr-and phenyl substituents on phosphine.^28,29^ This postulation was supported by two further NMR-scale reactions. Heating Ru-MACHO-BH (5, 0.02 mmol) in toluene-*d*_8_ with H_2_O (0.1 mL) for 2 h at 110 °C formed the same complex (assigned as 1A–**OH**), eliminating the possibility of the involvement of *tert*-butoxide ions in the product (Fig S56 and S57). Furthermore, the addition of KO*t*Bu (0.05 mmol) to 1 (0.02 mmol) in toluene-*d*_8_ and heating at 110 °C for 1 h showed signals corresponding to a deprotonated complex and degradation species (consistent with the literature),^30^ which, upon subsequent addition of H_2_O, also generated the complex identified as 1A–**OH** (Fig. S54 and S55). Replacing the atmosphere of Ar with CO and heating at 110 °C for 1.5 h resulted in the formation of the cationic dicarbonyl complex 1G with formate as a counterion (^1^H NMR: *δ* −6.28 ppm, ^31^P{^1^H} NMR: *δ* 59.2 ppm) with concomitant disappearance of complex 1A–**OH**. This is similar to literature reports of analogous species formed from complex 1 in the presence of acetate^31^ and bicarbonate^32^ anions. The structure of this species was confirmed by single-crystal X-ray diffraction to be 1G (Fig. 3). Continued heating led to the gradual decrease of 1G and concomitant increase in 1A–**OH**, with concurrent formation of CO_2_ and H_2_, as confirmed by GC-TCD analysis and ^1^H NMR spectroscopy (signal at *δ* 4.55 ppm for dissolved H_2_). These experiments suggest that under catalytic conditions, the precatalyst is activated in the presence of a base and reacts with water to form the hydroxide complex 1A–**OH**. The presence of a CO atmosphere then results in the formation of complex 1G from 1A–**OH**. Upon heating, CO is consumed *via* the water gas shift reaction to generate CO_2_ and H_2_. Consequently, the presence of 1G decreases, and 1A–**OH** increases due to the large excess of water.

Having developed some understanding of the organometallic transformations involved in the reported water–gas shift reaction, DFT calculations were performed to elucidate the reaction pathway. We have recently reported a part of this pathway in the context of the reverse water–gas shift reaction.^26^ For the present study, key reaction steps leading to the main products were calculated using complex 1A (Fig. 4) as the catalyst alongside H_2_O and CO as substrates. For consistency with previous work, we used the same level of DFT (see caption of Fig. 4 and the full details in the SI), including the solvent in the polarisable continuum model (THF – note that the bulk solvation effects modelled this way will be very similar for this solvent and xylene, which have been used for most of the experiments in the present work; test calculations using xylene as the solvent lead to deviations of less than 2 kcal mol^−1^ and to a reduced overall energy span of *ca.* 37 kcal mol^−1^, see Fig. S73, compared to 40 kcal mol^−1^ in THF, see Fig. 4).

The computational studies suggested that the active species 1A reacts with H_2_O to form 1A–**OH** as an off-cycle stable intermediate, consistent with the experimental observation (Fig. 2). It can also react with CO to form the dicarbonyl complex 1B*via* transition state **TSA-B**. It was found that excess water could provide enhanced stabilisation *via* hydrogen bonding between the complex 1A–**OH** and H_2_O, forming a 6-membered ring to form the off-cycle species 1A–**OH**–**H**_**2**_**O**. 1D is an interesting intermediate containing a formate ligand bound through the CH bond, which is then cleaved in **TSD-E**. Formate ligands are known to coordinate *via* an O atom,^32^ and indeed the corresponding isomer 1F is more stable than 1D. Whatever the binding mode of formate, an additional CO is bound more strongly, which leads to 1G, the lowest point on the profile. As the elimination of CO_2_ and H_2_ from 1F or 1G does not seem easily possible, we propose that 1G, once formed, can revert to 1D and complete turnover as described above. In such a scenario, 1F and 1G are off-cycle intermediates.

**Fig. 2:**
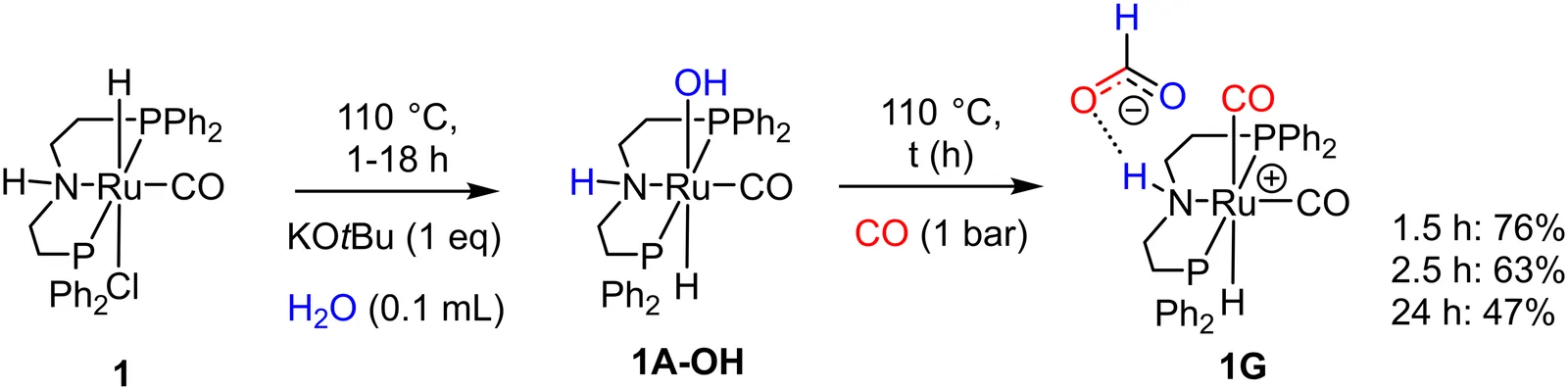
Reactivity of complex 1 with H_2_O and CO as monitored by ^1^H and ^31^P{^1^H} NMR spectroscopy.

**Fig. 3:**
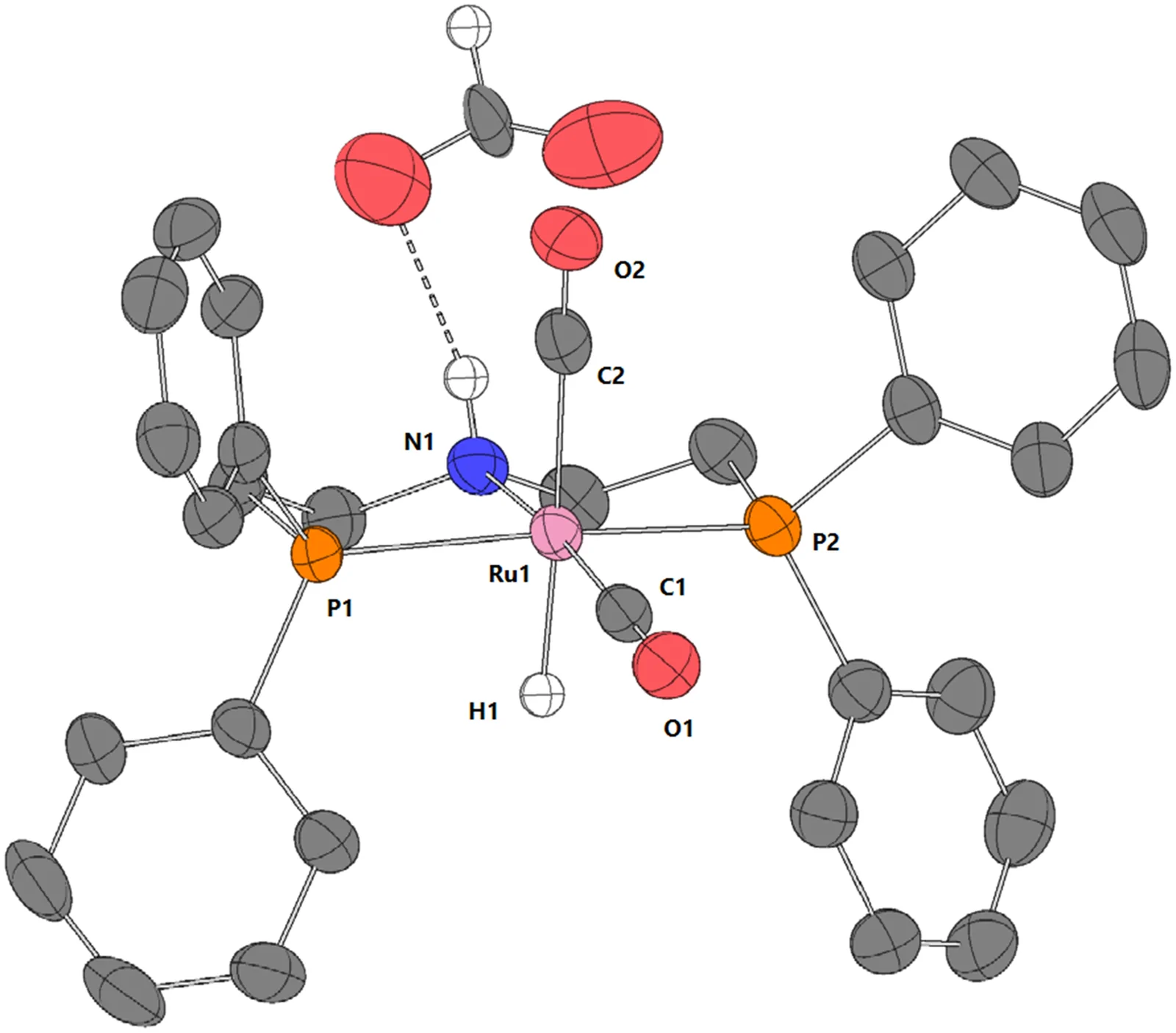
Single-crystal X-ray structure of the asymmetric unit cell of complex 1G. The ORTEP diagram plotted at 50% probability level. Selected hydrogen atoms have been omitted for clarity.

**Fig. 4:**
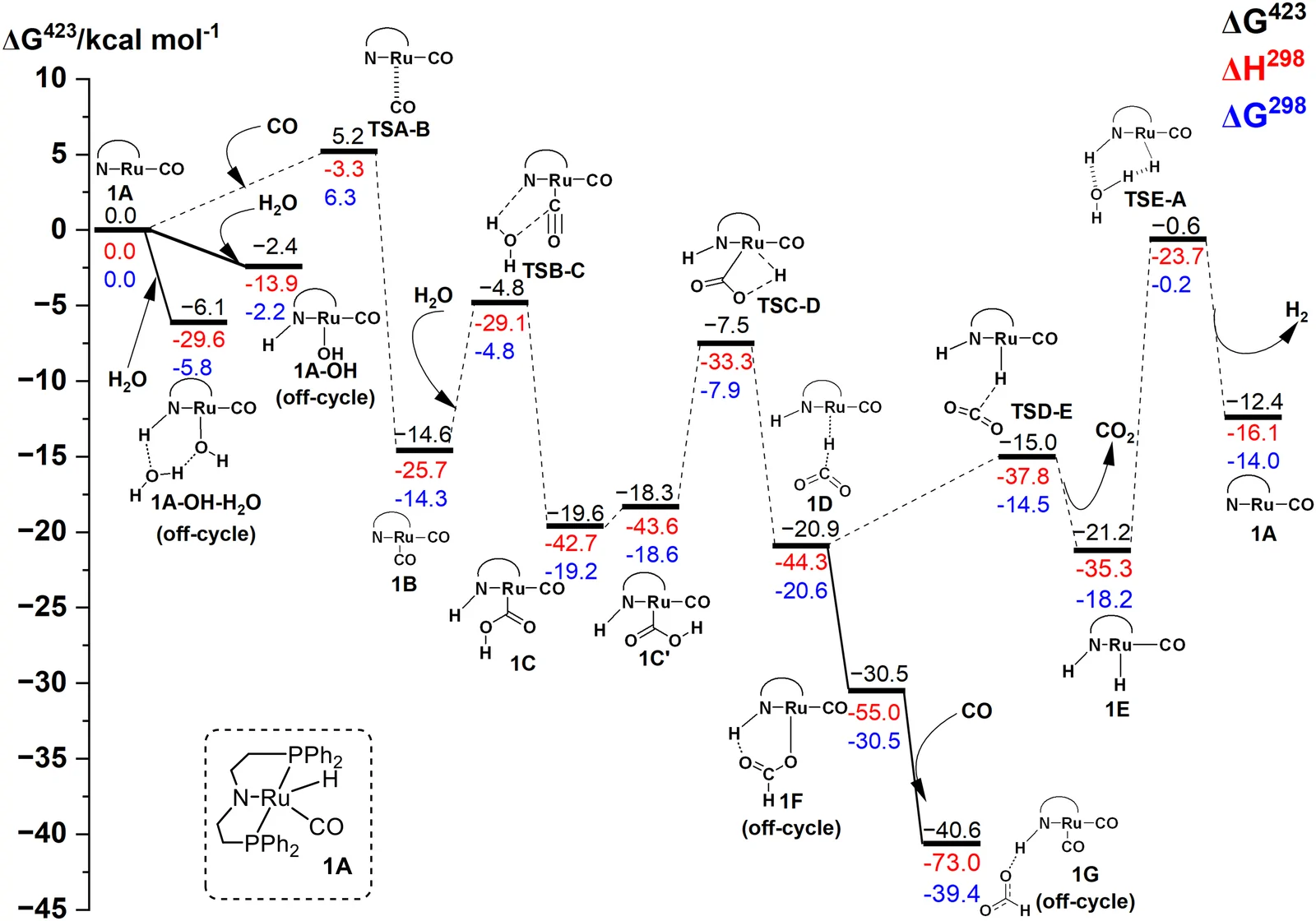
Energy profile showing the important mechanistic steps in the water–gas shift reaction using an activated Ru-MACHO catalyst, 1A (PBE0-D3(BJ)_PCM(THF)_/def2-TZVP//RI-BP86_PCM(THF)_/def2-SVP level). Δ*H* and Δ*G* are the relative enthalpies and free energies at the given temperature (superscript in K).

Interestingly, rather low barriers are predicted for the key elementary steps, OH activation and CO elimination (on the order of 10–12 kcal mol^−1^), and regeneration of 1A through H_2_ release, 20.6 kcal mol^−1^ from 1E to **TSE-A**. However, the rate-limiting states (based on Shaik and Kozuch's energy span model),^33^ need to include the lowest intermediate, even if it is off-cycle, *i.e.*1G in our case. The highest barrier is therefore between 1G and **TSE-A**, 40 kcal mol^−1^ in Fig. 4 using THF, and (as mentioned above) 37.2 kcal mol^−1^ in xylene. Such a barrier is, arguably, compatible with the observed activity under the reaction conditions (*i.e.* the elevated temperature).

Together, the NMR and DFT studies lead us to propose the mechanism outlined in Fig. 5. After base activation, the 5-coordinate complex 1A is formed from 1. 1A can react with water to form the off-cycle intermediate 1A–**OH** or can undergo CO insertion to form the dicarbonyl complex 1B. This intermediate can then react with water to generate the formate complex 1C, which can form the stable off-cycle intermediate 1G, proposed to be one of the resting states of the catalytic cycle. Release of CO_2_ then results in the dihydride complex 1E, and finally, H_2_ can be released to regenerate the active species 1A, which is the rate-determining step.

**Fig. 5:**
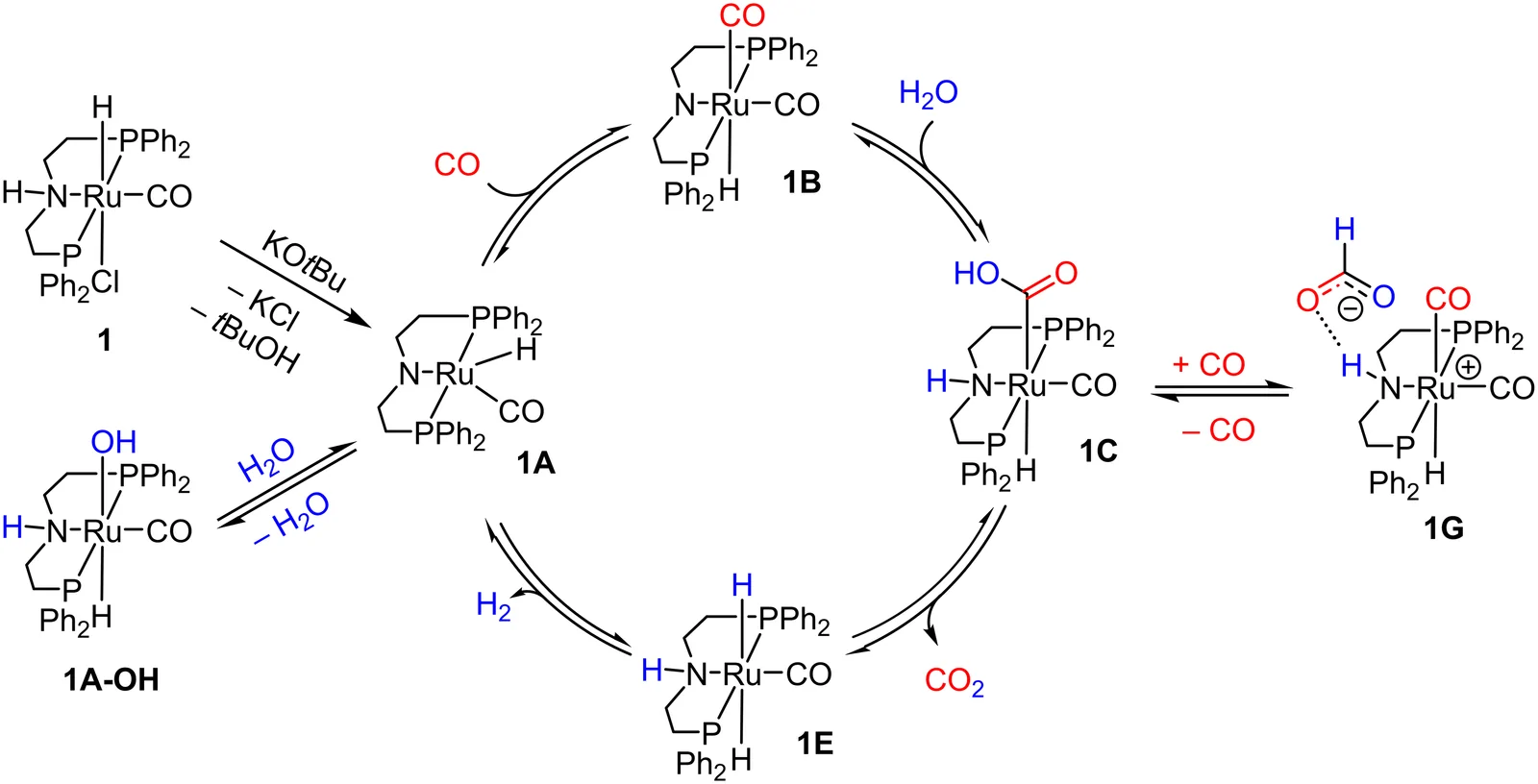
Proposed catalytic cycle for the WGS reaction using precatalyst 1.

Considering that the reactants and products are gaseous whereas the catalyst stays in the solution, we also studied catalyst recyclability to evaluate the catalyst's stability for this system. After each 24 h cycle (as per the conditions mentioned in Table 1, entry 11), the headspace gas was collected and analysed by GC-TCD. The reaction mixture was then frozen under liquid nitrogen, the atmosphere was replaced with fresh CO (1 bar), and the flask was reheated to 150 °C for the subsequent cycle. After each 24 h period, the gas was collected and the TON was recorded. This resulted in little observed loss of activity over 3 continuous cycles and a large decrease in TON for the fourth cycle. Significantly, this method of recycling resulted in a total TON of >6000 for H_2_ (Table 4). Upon acquiring the ^31^P{^1^H} NMR spectrum of the reaction mixture at the end of the reaction, the predominant signal was consistent with the literature-reported phosphorus signal of the oxidised ligand.^34^ We therefore suggest that catalyst oxidation and ligand dissociation are the primary factors contributing to catalyst deactivation.

**Table 4:** Recyclability studies of 1 for the WGS reaction^a^

Cycle	1	2	3	4
TON^b^	2370	2110	2020	420

a Conditions: 1 (0.01 mmol), KO*t*Bu (0.04 mmol), H_2_O (1 mL), xylene (1 mL), 150 °C, 18 h, 1.5 bar CO.

b TONs determined by GC-TCD analysis of headspace ((mmol of H_2_)/(mmol of catalyst used)). The TONs have been rounded to the nearest 10.

## Conclusion

In summary, we report the homogeneous water–gas shift reaction using the Ru-MACHO catalyst under substantially milder conditions (150 °C, 0.5 bar over-pressure of CO) compared to previously reported homogeneous and heterogeneous systems, achieving a TON of 2570. Dual-chamber reactor engineering enabled quantitative CO_2_ removal and the production of high-purity H_2_. The catalyst recyclability was also demonstrated four times, with a cumulative TON of >6000. Furthermore, this process was coupled with transfer hydrogenation of ketones to make secondary alcohols using CO and H_2_O as reducing agents, forming CO_2_ as the only by-product that can be easily removed from the reaction in contrast to the conventional transfer hydrogenation system, where acetone is generated as the by-product while using isopropanol. Mechanistic studies have been conducted by performing NMR studies and DFT computations to provide insights into the organometallic species formed in the catalytic cycle.

## Conflicts of interest

There are no conflicts to declare.

## Supplementary Material

CY-OLF-D6CY01293K-s001

CY-OLF-D6CY01293K-s002

## Data Availability

The research data supporting this publication can be accessed from the University of St Andrews Research Portal https://doi.org/10.17630/1f7285b4-3457-47dc-a087-e33858e492a6. Supplementary information (SI) is available. See DOI: https://doi.org/10.1039/d6cy01293k. CCDC 2553626 contains the supplementary crystallographic data for this paper.^35^
