# Supplementary material for: Atmospheric pressure water–gas shift reaction and transfer hydrogenation of ketones using a ruthenium pincer catalyst
Source: Catal Sci Technol. 2026 Sep 25. Online ahead of print. doi: 10.1039/d6cy01293k (PMC13684356; doi:10.1039/d6cy01293k)
Supplement: Supplementary file 1 [file CY-OLF-D6CY01293K-s002.pdf]

Supplementary Information (SI) for Catalysis Science & Technology.
This journal is © The Royal Society of Chemistry

Atmospheric Pressure Water Gas Shift Reaction and Transfer Hydrogenation of Ketones Using a Ruthenium Pincer Catalyst

James Luk[a], Luming Guo[a], Michael Bühl,[a]* and Amit Kumar*[a]

[a] EaStCHEM, School of Chemistry, University of St Andrews, North Haugh, KY16 9ST St Andrews, (UK)

E-mail: Amit Kumar: ak336@st-andrews.ac.uk; Michael Bühl: buehl@st-andrews.ac.uk.

Contents

1. General Information 2
2. General Procedures 3
   2.1. General procedure for WGSR optimisation 3
   2.2. General procedure for H-flask reactions 3
   2.3. General procedure for transfer hydrogenation 4
   2.4. General procedure for catalyst recycling studies 4
   2.5. General procedure for NMR scale reactions 4
3. GC-TCD data 5
   3.1. GC-TCD data for WGSR optimisation 5
   3.2. GC-TCD data for H-flask reactions 20
   3.3. GC-TCD data for catalyst recycling studies 22
   3.4. GC-TCD data for NMR scale reactions 24
4. NMR data 25
   4.1. Representative NMR data for WGS reactions 25
   4.2. Representative NMR data for aqueous side of H-flask reactions 26
   4.3. NMR data for transfer hydrogenation reactions 27
   4.4. NMR data for NMR scale reactions 30
5. GC-MS data 35
   5.1. GC-MS data for transfer hydrogenation reactions 35
6. Crystallography data 40
7. Computational data 41
   7.1. General Considerations 41
   7.2. Computational Raw Data 43
8. References 68

1. General Information

All manipulations were carried out under an inert atmosphere of argon or nitrogen using standard Schlenk and glove-box techniques unless specified. All chemicals (e.g. diamines, diols, formic acid, etc) were purchased from Sigma-Aldrich, Thermo Fisher, TCI or Strem, unless stated otherwise, and used as received. Complexes **2**,[1] **3**,[1] **4**,[1] **6**[2] and **8**[3] were synthesised according to literature procedures. THF and toluene were dried by a solvent purification system and degassed using freeze-pump-thaw technique before being dried on 4Å molecular sieves before use, while anisole was only dried on 4Å molecular sieves and degassed using freeze-pump-thaw technique.

All NMR spectroscopic experiments were carried out at 298 K using a Bruker Avance II 400 MHz or a Bruker AV-III HD 500 MHz, unless stated otherwise, and reported in ppm (δ). NMR spectroscopy abbreviations: b - broad, s – singlet, d – doublet, t – triplet, q – quartet, m – multiplet. Error in the estimation of conversion and yields is ±5%. Gas Chromatography (GC-MS) was performed on an Agilent 8860 series GC using an Agilent 5977B GC/MSD, fitted with an Agilent J&W HP-5ms Ultra Inert GC-column (30 m, 0.25 mm, 0.25 µm, 7-inch cage). Helium was used as carrier gas with a column flow of 12 mL/min. Inlet temperature (250 °C) and initial oven temperature (50 °C) were used for an initial hold of 4.2 minutes, before an oven ramp of 10 °C/min for 15 minutes until 250 °C where it was held until 39 minutes. The gas headspace was analysed using an Agilent 8860 gas chromatography (columns: Porapak Q, 2.74 m × 2 mm and 5 Å molecular sieves, 1.83 m × 2 mm, temperature: 60 °C, 2 min, ramped to 150 °C over 4.5 min) equipped with a thermal conductivity detector (TCD) with Ar (30 mL/min) as the carrier gas. Calibration was carried out with certified calibration mixtures of H_2 and CO, and CO_2. Yields were quantified by first determining gas concentrations from GC-TCD peak areas using external calibration, then converting to moles using the ideal gas law based on the reactor headspace volume (mL) and reaction conditions. Due to the large excess of CO_2 and H_2, the estimation of conversion with high accuracy was not possible under our settings.

Single crystals were obtained by vapour diffusion of toluene/hexane. SCXRD was performed and data were obtained by Crystallography Service, School of Chemistry, University of St Andrews. Data were solved using SHELXT[4] direct methods and refined by full-matrix least squares against F2 using SHELXL.[5] Crystallographic data are summarised in Table S1. Further experimental and refinement details can be found in the corresponding CIF files. The supplementary data for the crystallographic data can be found at CCDC 2553626.

2. General Procedures

2.1. General procedure for WGSR optimisation

In a glovebox, the desired complex (0.01 mmol), KO*t*Bu (0.04 mmol) and solvent (1 mL) were added to a 250 mL J-Young flask. The flask was sealed, removed from the glovebox and connected to a Schlenk line. Under Ar, H_2O (1 mL) was added to the reaction mixture before the flask was charged with an overpressure of 0.5 bar CO under 1 bar Ar such that the final pressure was (2:1 Ar:CO) and sealed. The reaction mixture was then stirred for 18 h at 150 °C before being allowed to cool. The gas headspace was then taken through a syringe and analysed by GC-TCD.

2.2. General procedure for H-flask reactions

Complex **1** (0.01 mmol) and KO*t*Bu (0.04 mmol) were added to one side (A) of an H-flask before the flask was vacuum cycled with Ar 3 times. Under Ar, xylene (1 mL) and H_2O (1 mL) were added to "side A", while a 30 wt% aqueous solution of ethanolamine (2 mL) was added to "side B". The reaction mixture was frozen under liquid N_2 and the atmosphere removed under vacuum. The flask was then sealed, allowed to warm to RT and then charged with 1 bar CO and resealed. "Side A" was stirred for 18 h at 150 °C and "side B" was stirred at RT. The flask was then allowed to cool, and the gas headspace was then taken through a syringe and analysed by GC-TCD. The experimental setup has also been shown below.

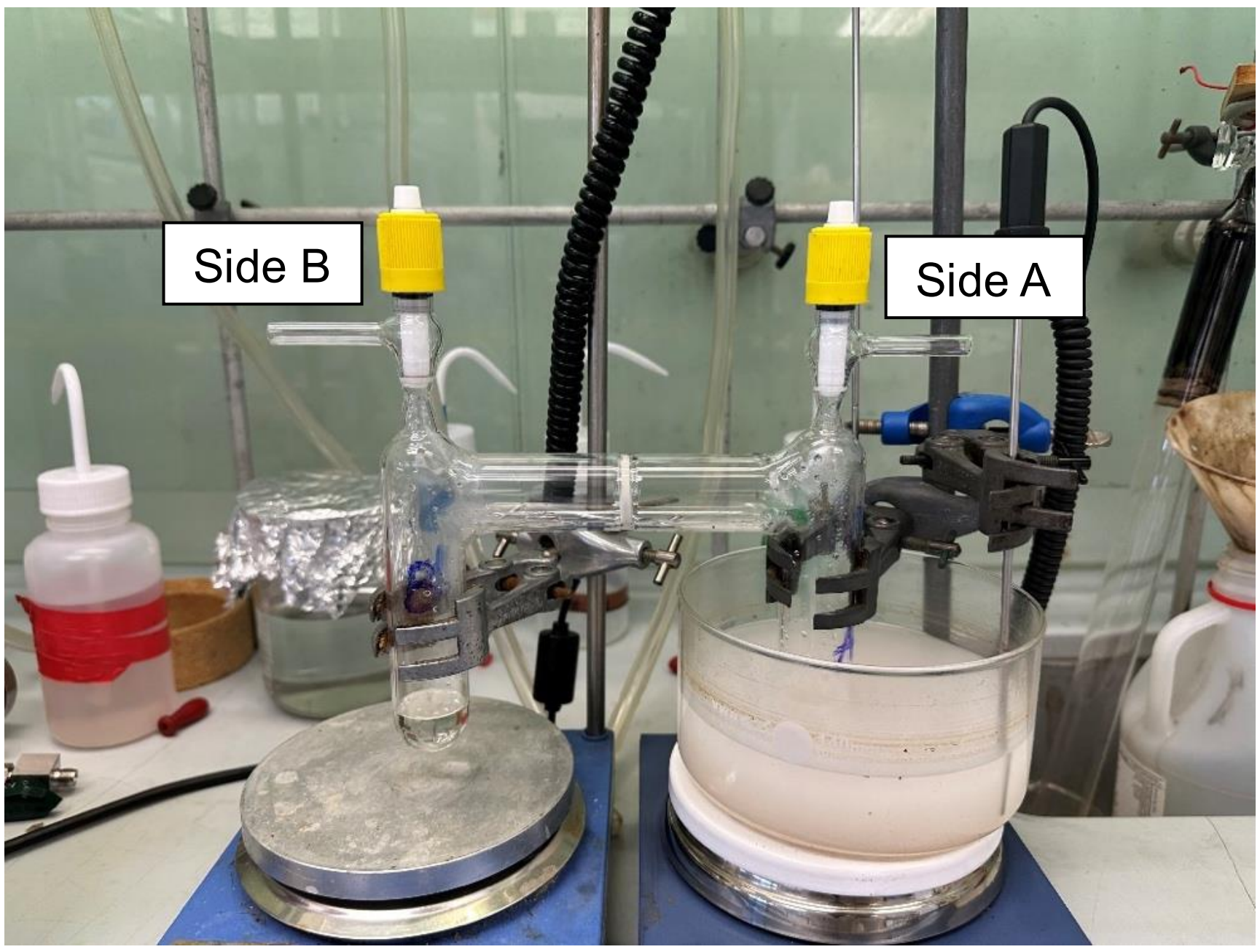

Figure S1: Image of H-flask reaction setup.

2.3. General procedure for transfer hydrogenation

In a glovebox, the complex **1** (0.01 mmol), KO*t*Bu (0.04 mmol), xylene (1 mL) and desired ketone were added to a 100 mL J-Young flask. The flask was sealed, removed from the glovebox and connected to a Schlenk line. Under Ar, H_2O (1 mL) was added to the reaction mixture before the flask was charged with an overpressure of 0.5 bar CO under 1 bar Ar such that the final pressure was (2:1 Ar:CO) and sealed. The reaction mixture was then stirred for 18 h at 150 °C before being allowed to cool. The reaction mixture was analysed by ^{1}H NMR spectroscopy and GC-MS.

2.4. General procedure for catalyst recycling studies

In a glovebox, the complex **1** (0.01 mmol), KO*t*Bu (0.04 mmol), and xylene (1 mL) were added to a 250 mL J-Young flask. The flask was sealed, removed from the glovebox and connected to a Schlenk line. Under Ar, H_2O (1 mL) was added to the reaction mixture before the flask was frozen under liquid N_2 and the atmosphere removed under vacuum. The flask was then sealed, allowed to warm to RT and then charged with 1 bar CO and resealed. The reaction mixture was then stirred for 18 h at 150 °C before being allowed to cool. The gas headspace was then taken through a syringe and analysed by GC-TCD. The flask was then frozen under liquid N_2, and the headspace replaced with CO. This process was repeated after each cycle.

2.5. General procedure for NMR scale reactions

In a glovebox, complex **1** (0.02 mmol) and d_8-toluene (~0.5 mL) were added to a J-Young NMR tube and characterised by NMR spectroscopy. On a Schlenk line, an aqueous solution of KO*t*Bu (0.05 mmol) was added to the NMR tube and heated (110 °C), and the sample was analysed by NMR spectroscopy at t = 1.5 h and 18 h. The J-Young NMR tube was then frozen under liquid N_2, and the atmosphere removed under vacuum and the tube sealed. The tube was then allowed to warm to RT and charged with CO (1 bar) before being heated (110 °C) and the mixture analysed at t = 1.5, 2.5, 18 h by NMR spectroscopy. The resultant headspace of the NMR tube was then acquired through a syringe and analysed by GC-TCD.

3. GC-TCD data

3.1. GC-TCD data for WGSR optimisation

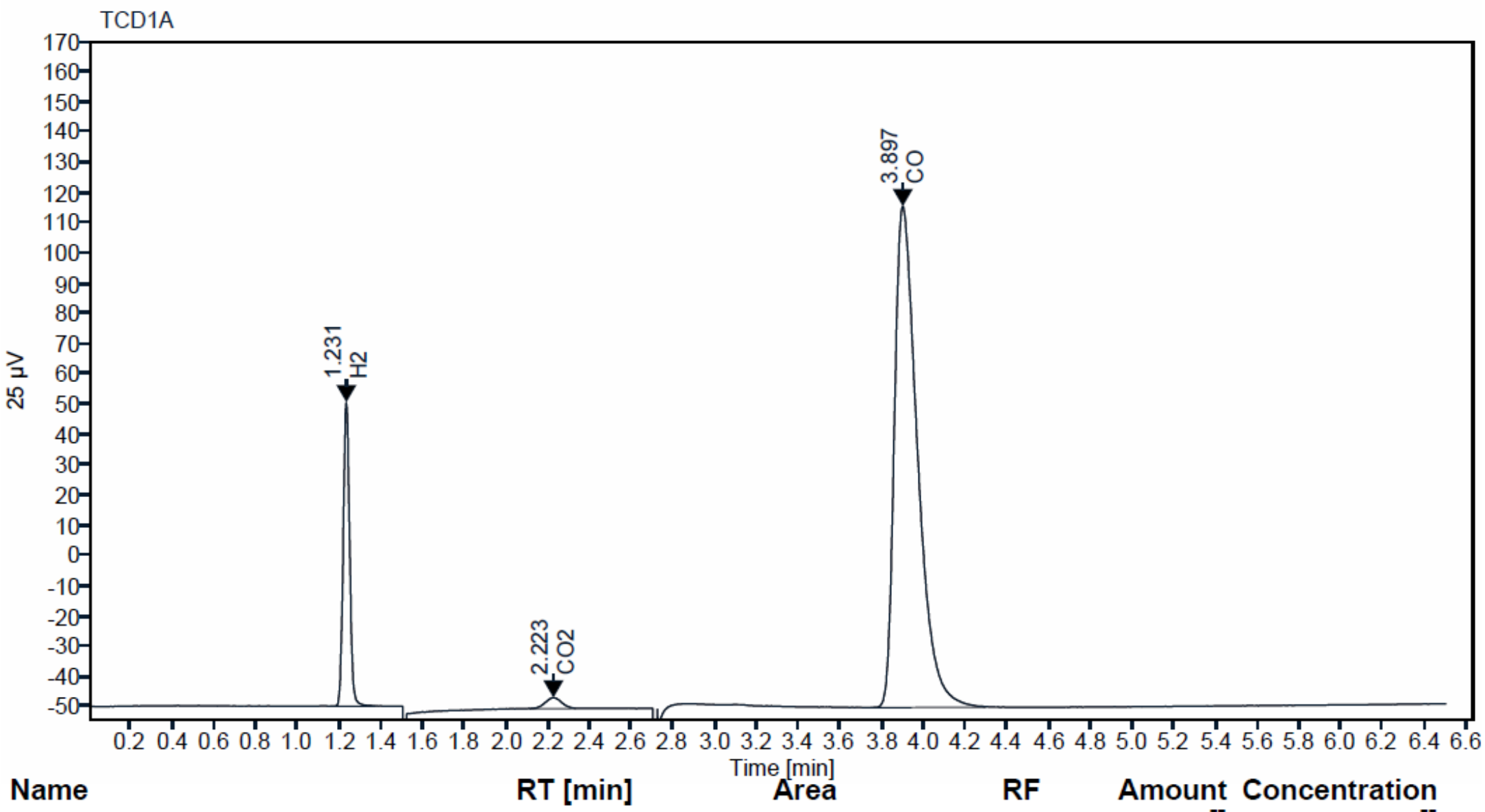

Name	RT [min]	Area	RF	Amount []	Concentration []
H2	1.231	215.0999	455.26827	0.472	0.4725
CO2	2.223	20.1737	20.1737	46.05372	0.438
CO	3.897	1273.0754	38.67660	32.916	32.9159

Figure S2: Gas chromatograph for Table 1, entry 1.

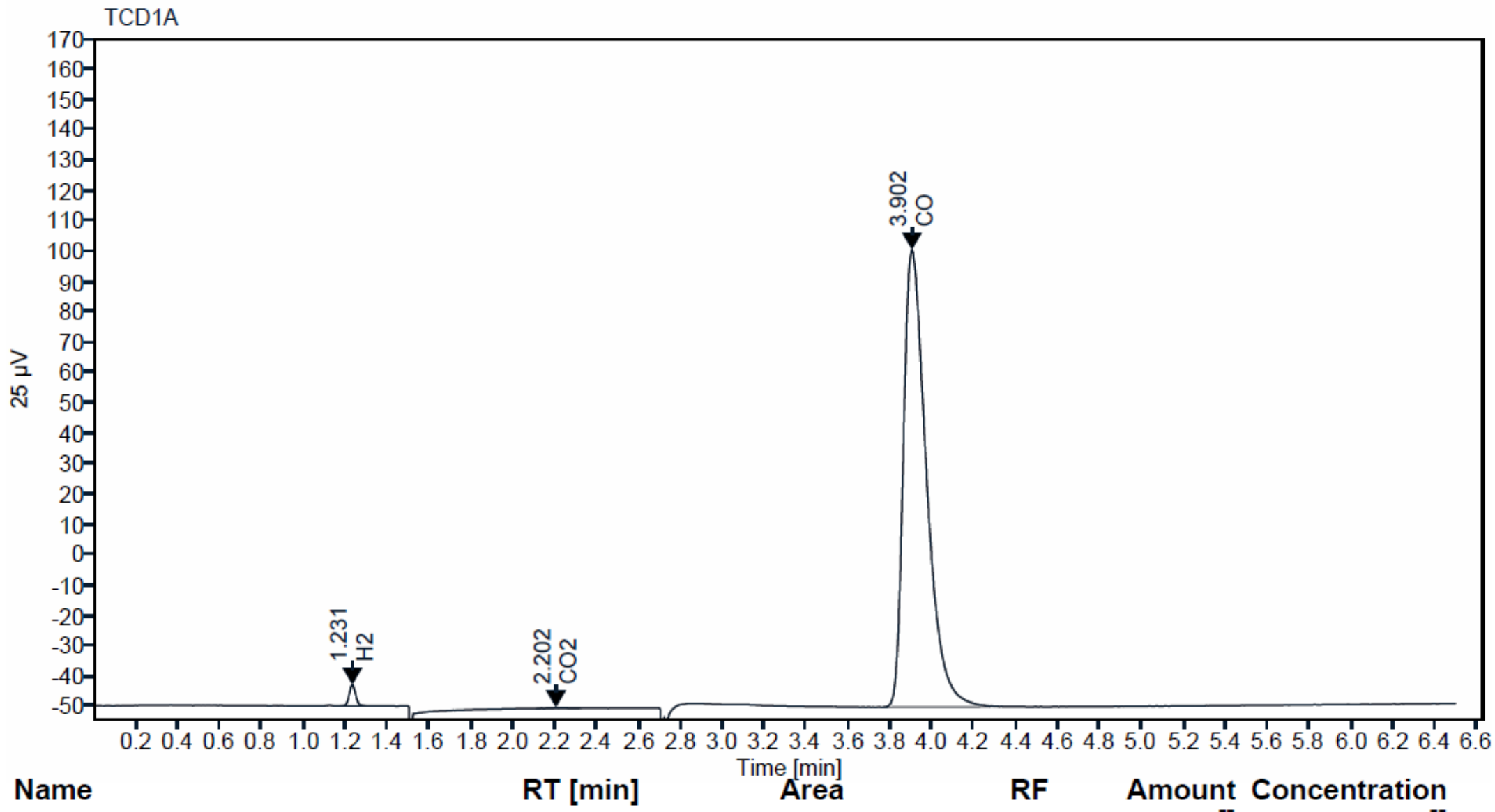

Name	RT [min]	Area	RF	Amount []	Concentration []
H2	1.231	14.8808	455.26827	0.033	0.0327
CO2	2.202	1.0095	46.05372	0.022	0.2192
CO	3.902	1149.7903	38.67660	29.728	29.7283

Figure S3: Gas chromatograph for Table 1, entry 2.

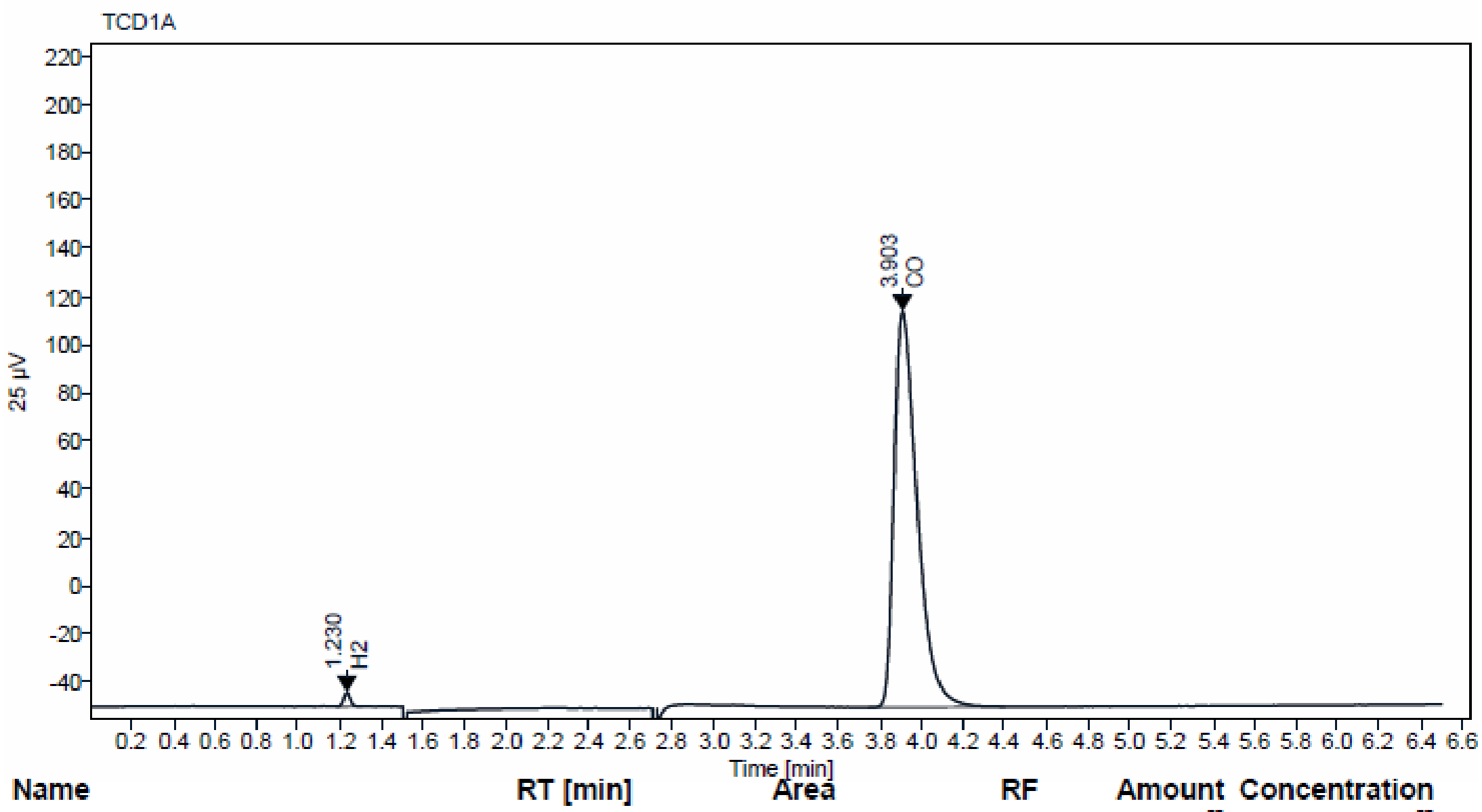

Name	RT [min]	Area	RF	Amount []	Concentration []
H2	1.230	12.5156	455.26827	0.027	0.0275
CO	3.903	1265.8724	38.67660	32.730	32.7297

Figure S4: Gas chromatograph for Table 1, entry 3.

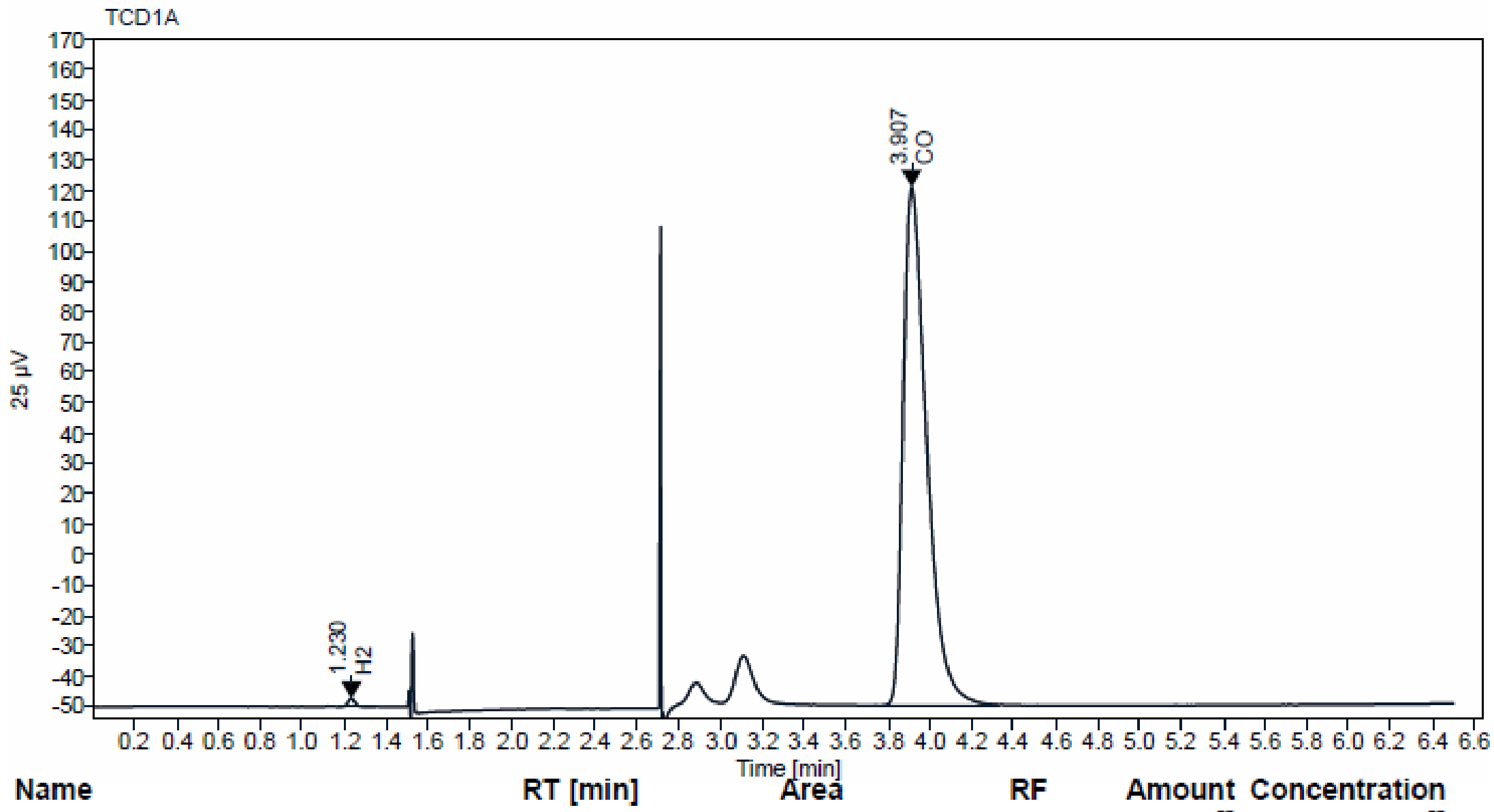

Name	RT [min]	Area	RF	Amount []	Concentration []
H2	1.230	5.9362	455.26827	0.013	0.0130
CO	3.907	1335.9387	38.67660	34.541	34.5413

Figure S5: Gas chromatograph for Table 1, entry 4 (peaks at 2.9 and 3.3 min are due to trace air induced during gas sampling).

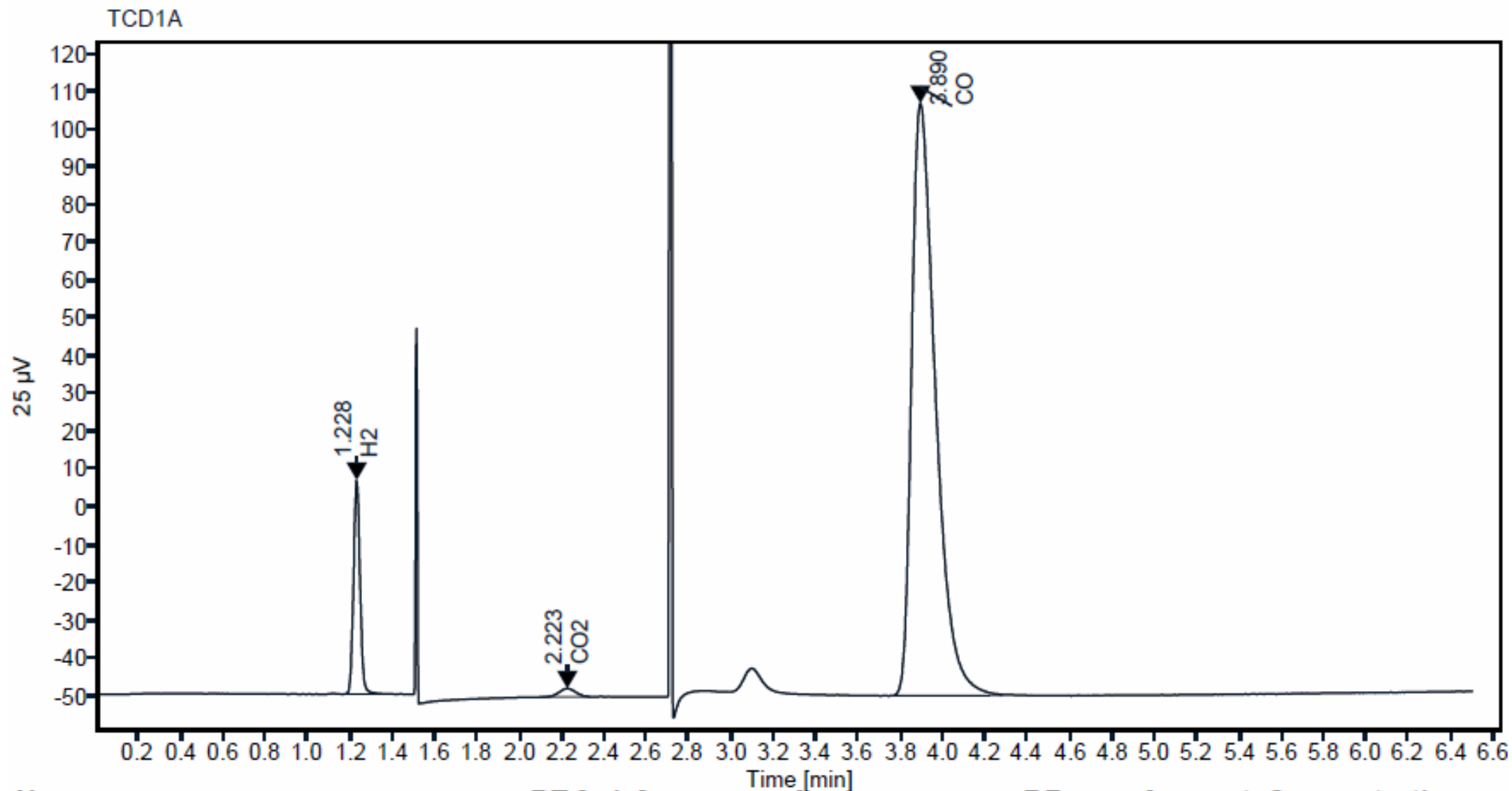

Name	RT [min]	Area	RF	Amount []	Concentration []
H2	1.228	122.2347	455.26827	0.268	0.2685
CO2	2.223	12.2398	46.05372	0.266	2.6577
CO	3.890	1227.9398	38.67660	31.749	31.7489

Figure S6 : Gas chromatograph for Table 1, entry 5 (peaks at 2.9 and 3.3 min are due to trace air induced during gas sampling).

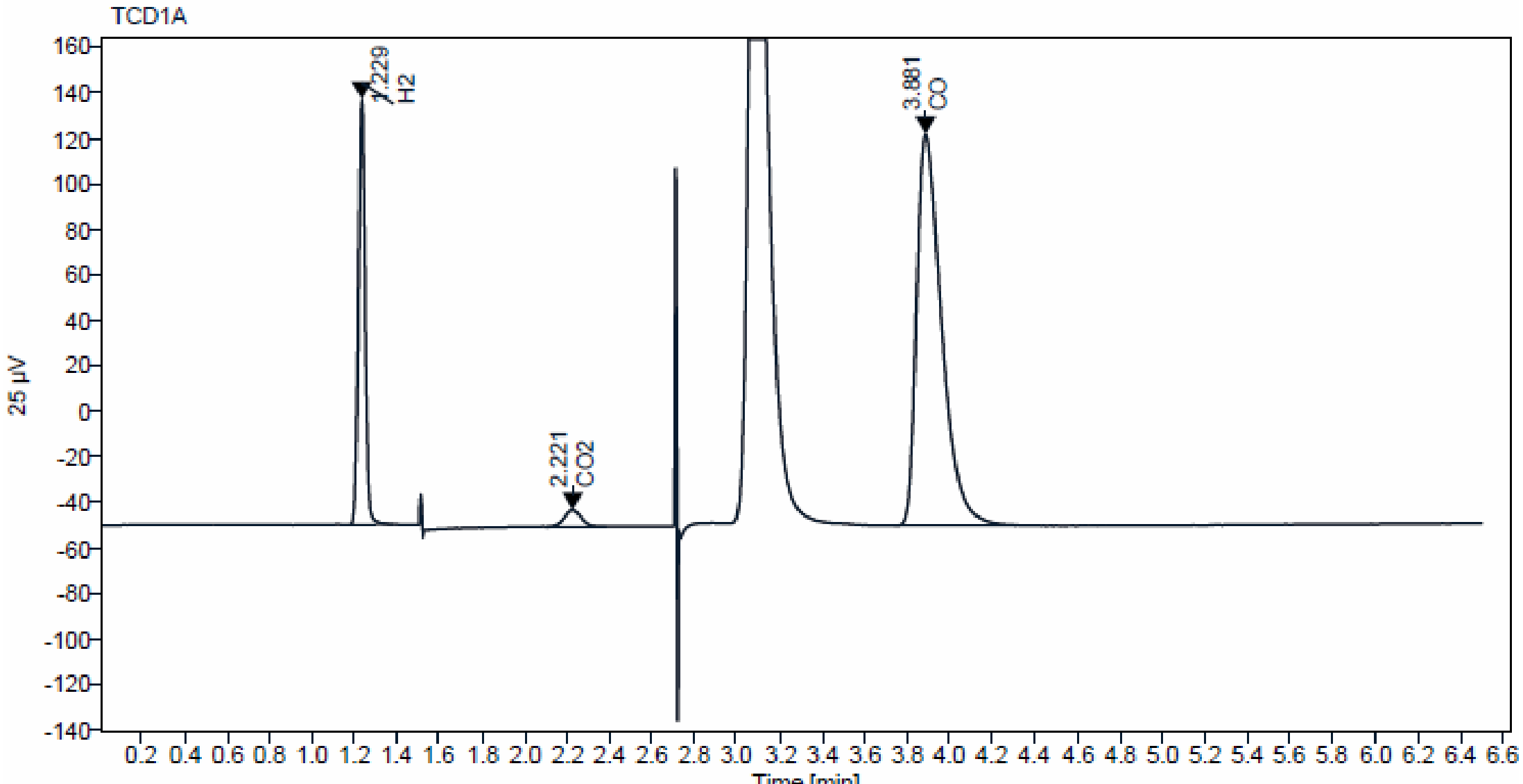

Name	RT [min]	Area	RF	Amount []	Concentration []
H2	1.229	409.0737	455.26827	0.899	0.8985
CO2	2.221	42.5914	46.05372	0.925	9.2482
CO	3.881	1403.4358	38.67660	36.286	36.2864

Figure S7: Gas chromatograph for Table 1, entry 6 (peaks at 2.9 and 3.3 min are due to trace air induced during gas sampling).

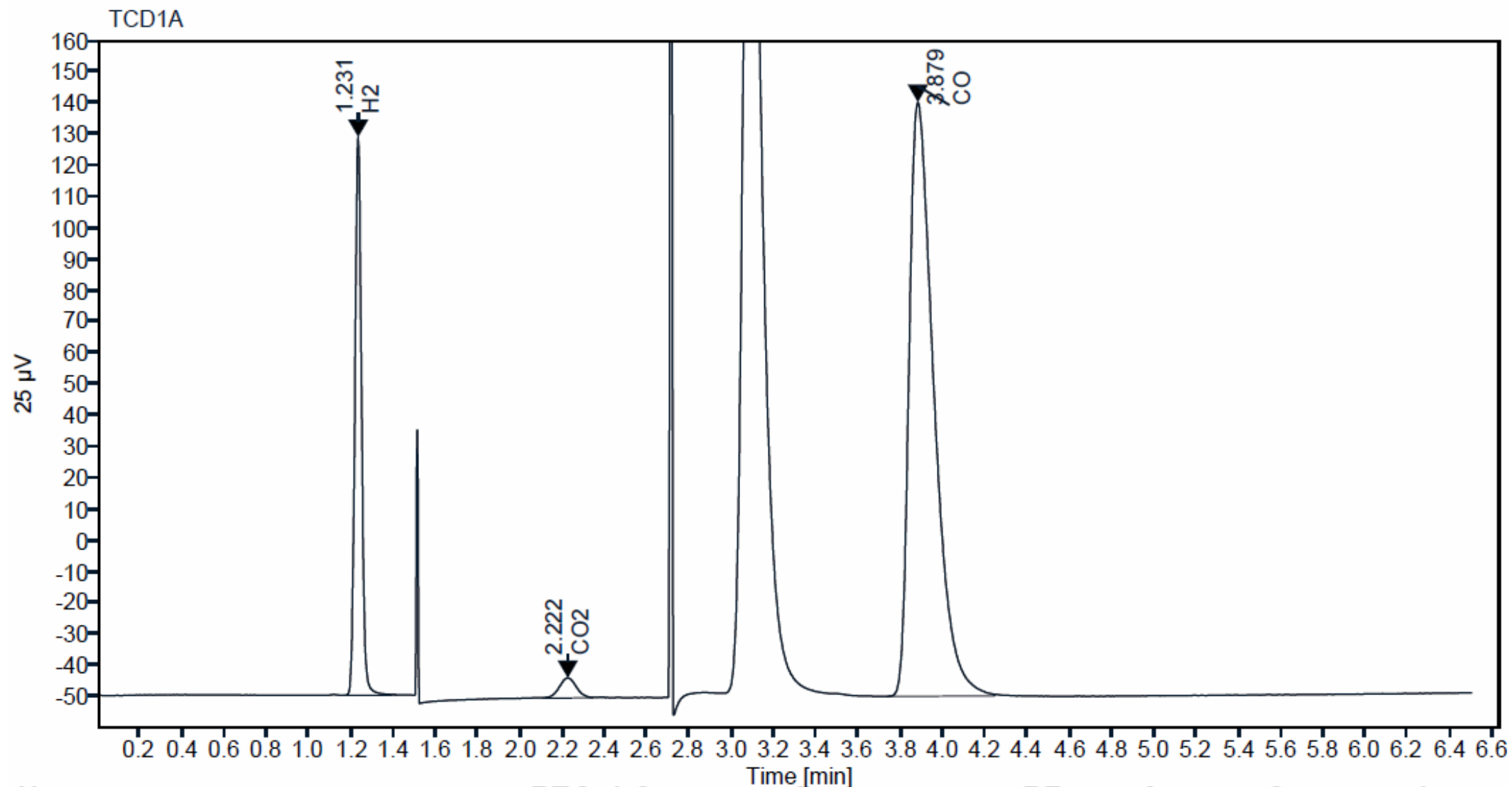

Name	RT [min]	Area	RF	Amount []	Concentration []
H2	1.231	391.8497	455.26827	0.861	0.8607
CO2	2.222	36.0041	46.05372	0.782	7.8178
CO	3.879	1552.3589	38.67660	40.137	40.1369

Figure S8: Gas chromatograph for Table 1, entry 7 (peak 3.3 min is due to reaction being set up under N_2 opposed to Ar).

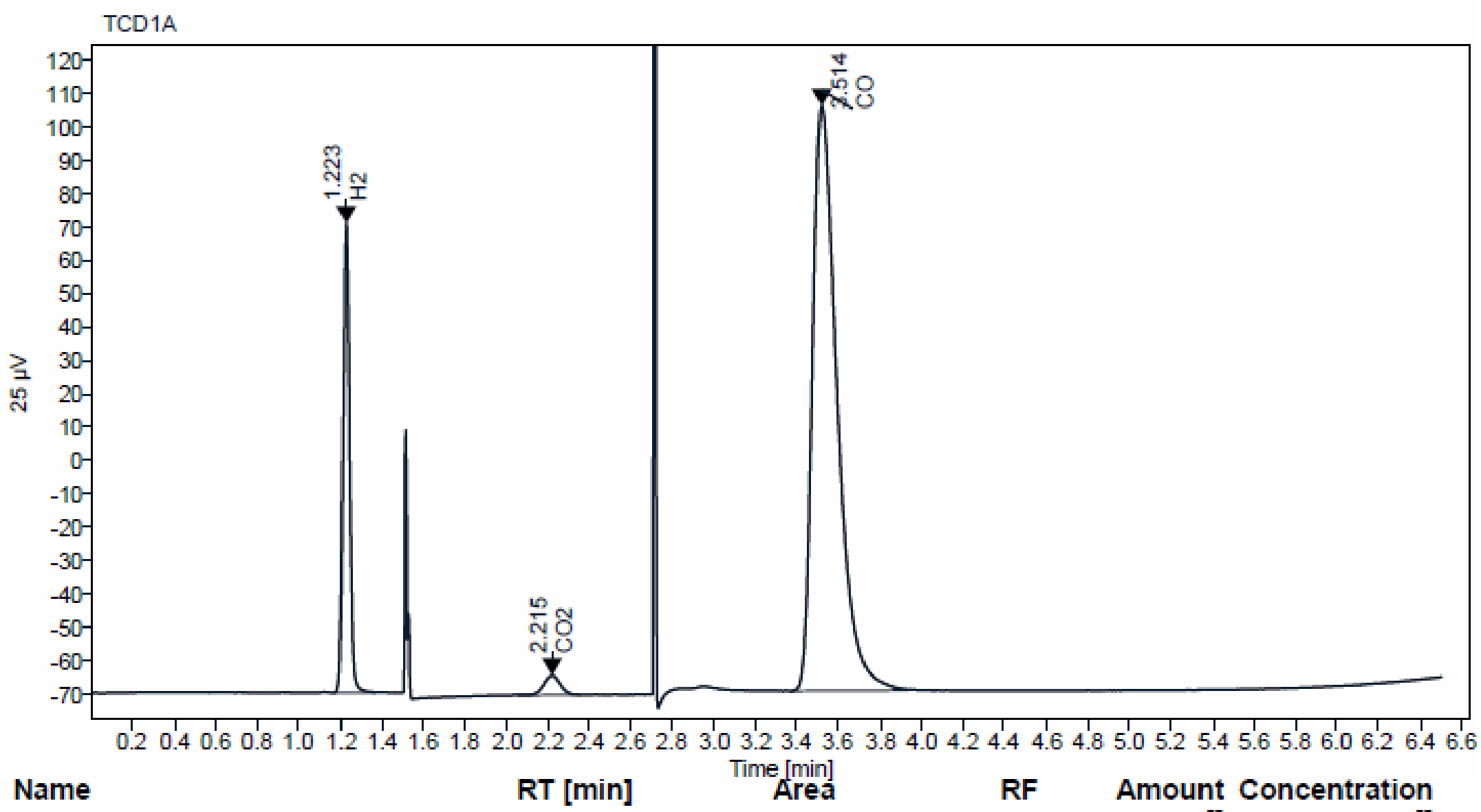

Name	RT [min]	Area	RF	Amount []	Concentration []
H2	1.223	304.3429	455.26827	0.668	0.6685
CO2	2.215	32.5078	32.5078	46.05372	0.706
CO	3.514	1436.0484	38.67660	37.130	37.1296

Figure S9: Gas chromatograph for Table 1, entry 8.

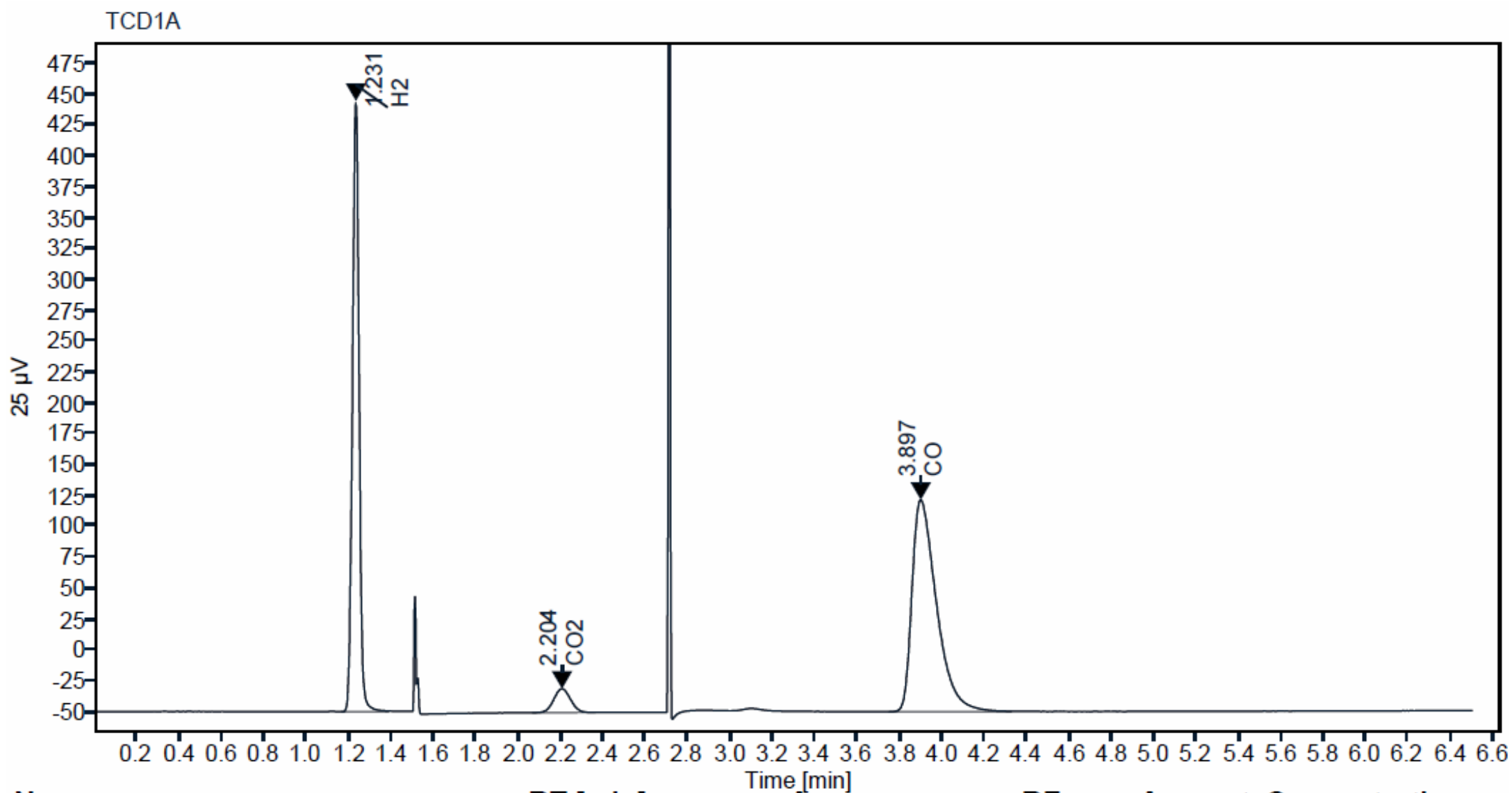

Name	RT [min]	Area	RF	Amount []	Concentration []
H2	1.231	1078.0077	455.26827	2.368	2.3679
CO2	2.204	106.5477	46.05372	2.314	23.1355
CO	3.897	1377.1070	38.67660	35.606	35.6057

Figure S10: Gas chromatograph for Table 1, entry 9.

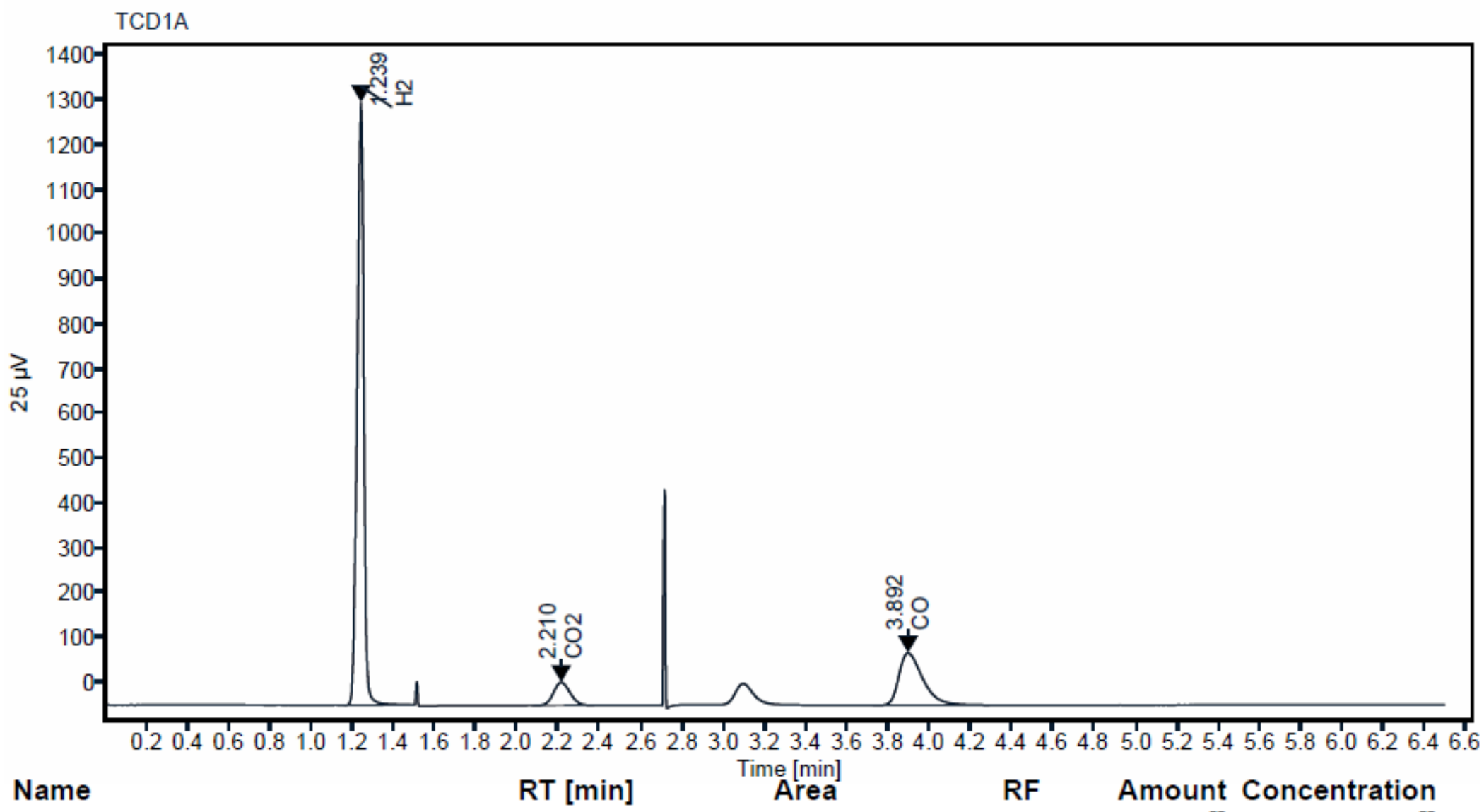

Name	RT [min]	Area	RF	Amount []	Concentration []
H2	1.239	3019.8755	455.26827	6.633	6.6332
CO2	2.210	288.0486	46.05372	6.255	62.5462
CO	3.892	937.2745	38.67660	24.234	24.2336

Figure S11: Gas chromatograph for Table 1, entry 10 (peaks at 2.9 and 3.3 min are due to trace air induced during gas sampling).

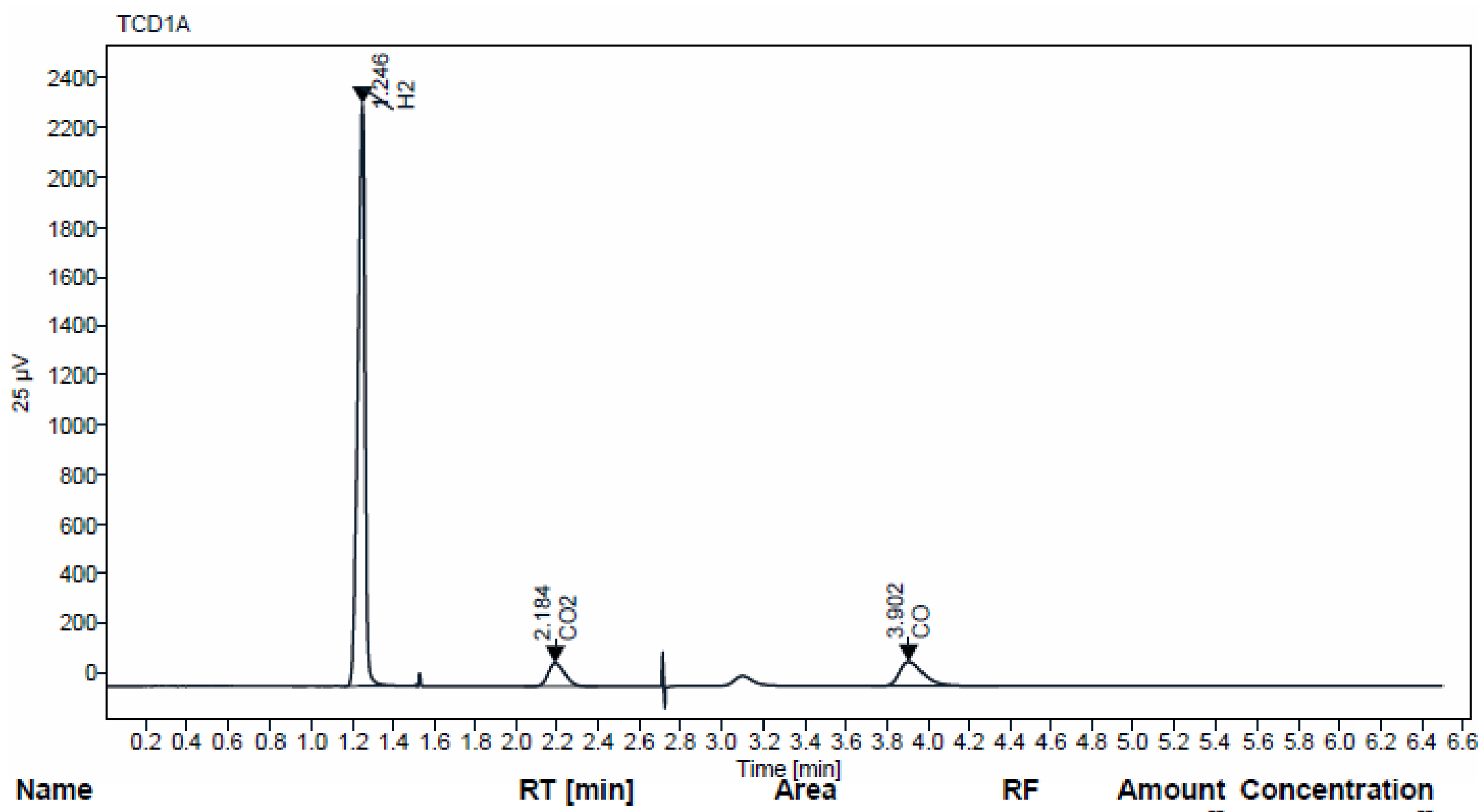

Name	RT [min]	Area	RF	Amount []	Concentration []
H2	1.246	5484.7189	455.26827	12.047	12.0472
CO2	2.184	538.0073	46.05372	11.682	116.8217
CO	3.902	809.8818	38.67660	20.940	20.9398

Figure S12: Gas chromatograph for Table 1, entry 11 (peaks at 2.9 and 3.3 min are due to trace air induced during gas sampling).

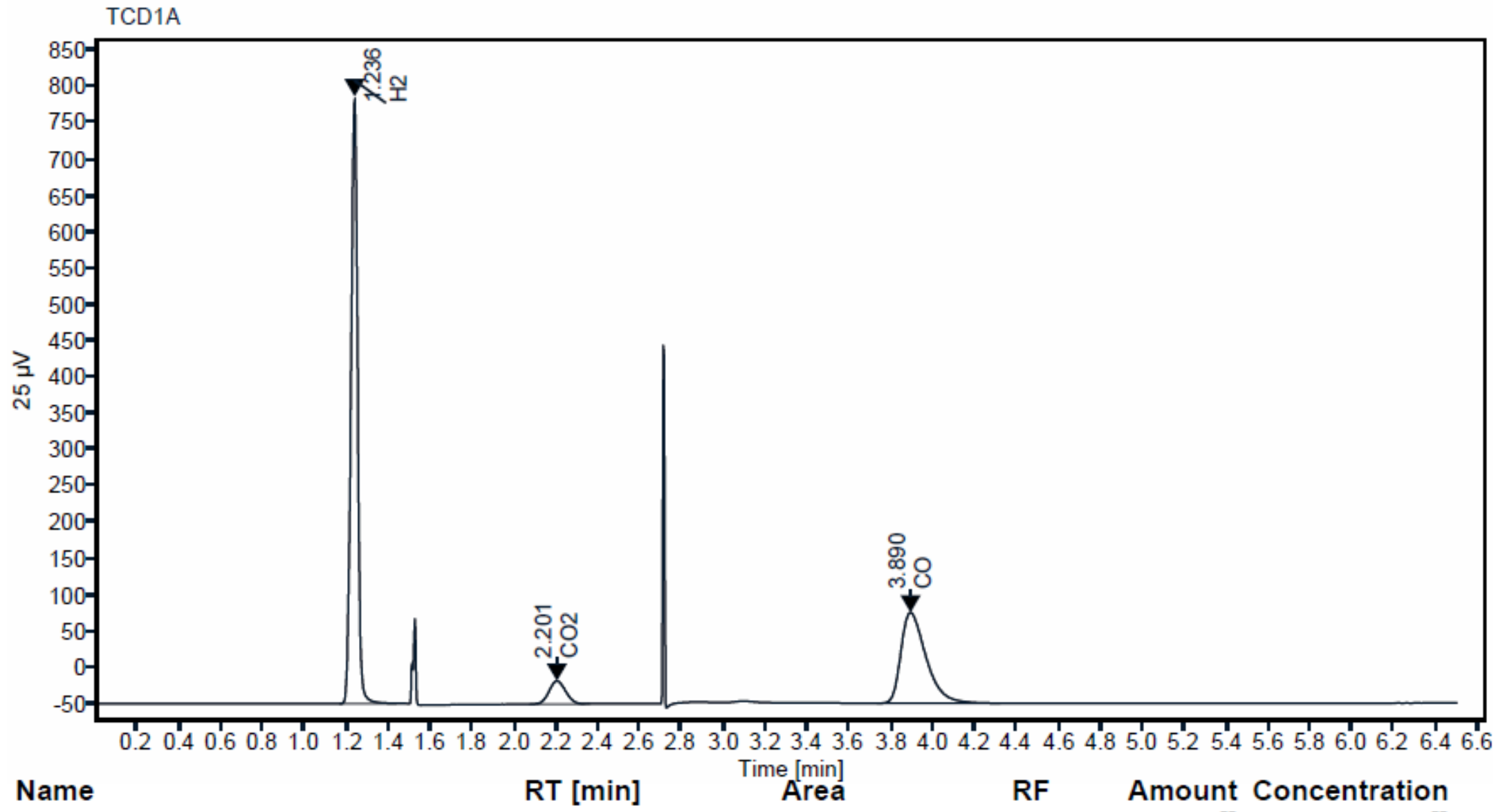

Name	RT [min]	Area	RF	Amount []	Concentration []
H2	1.236	1836.3829	455.26827	4.034	4.0336
CO2	2.201	175.8675	46.05372	3.819	38.1875
CO	3.890	1044.6281	38.67660	27.009	27.0093

Figure S13: Gas chromatograph for Table 1, entry 12.

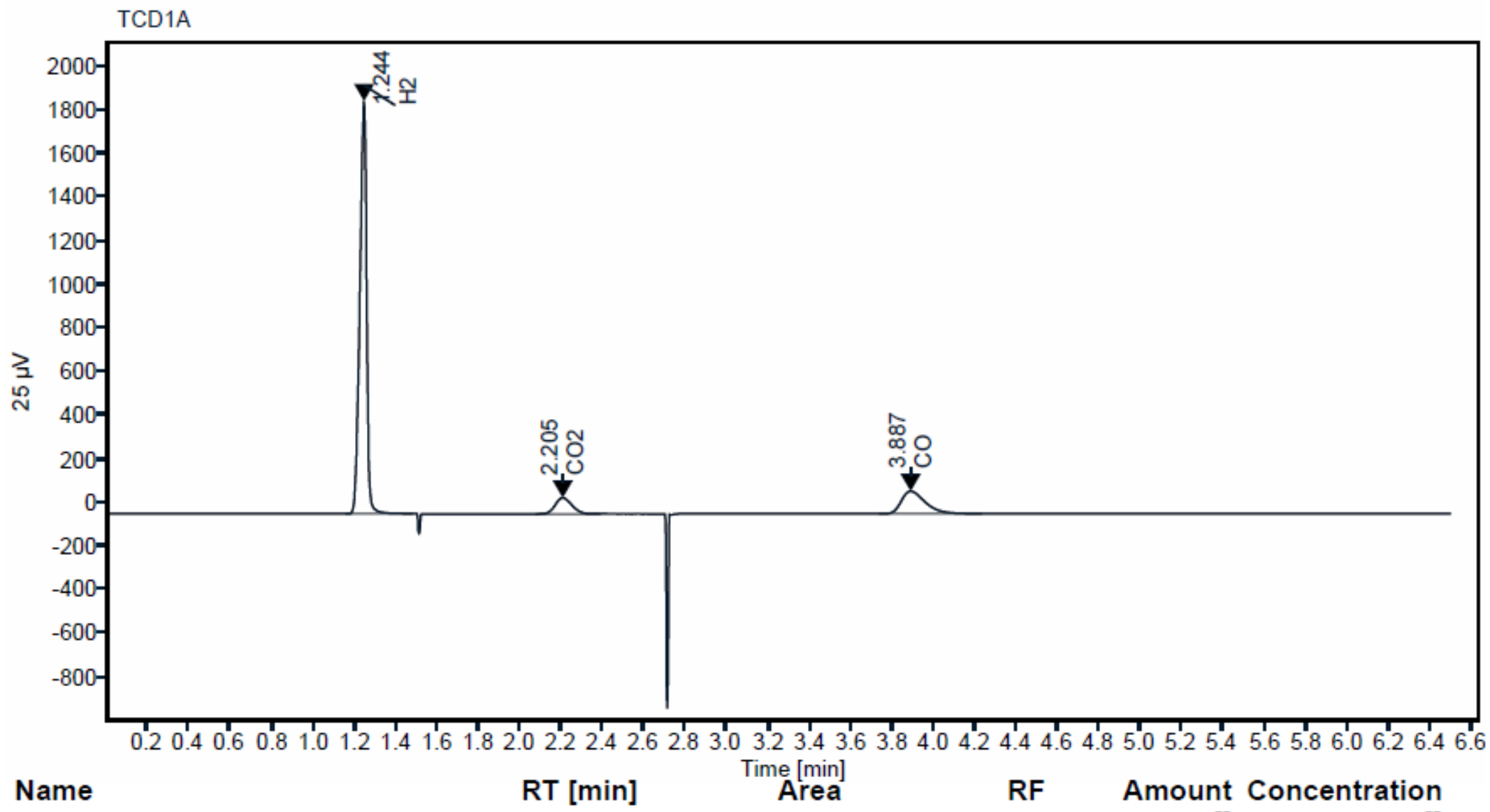

Name	RT [min]	Area	RF	Amount []	Concentration []
H2	1.244	4330.4035	455.26827	9.512	9.5118
CO2	2.205	411.6803	46.05372	8.939	89.3913
CO	3.887	839.9914	38.67660	21.718	21.7183

Figure S14: Gas chromatograph for Table 1, entry 13.

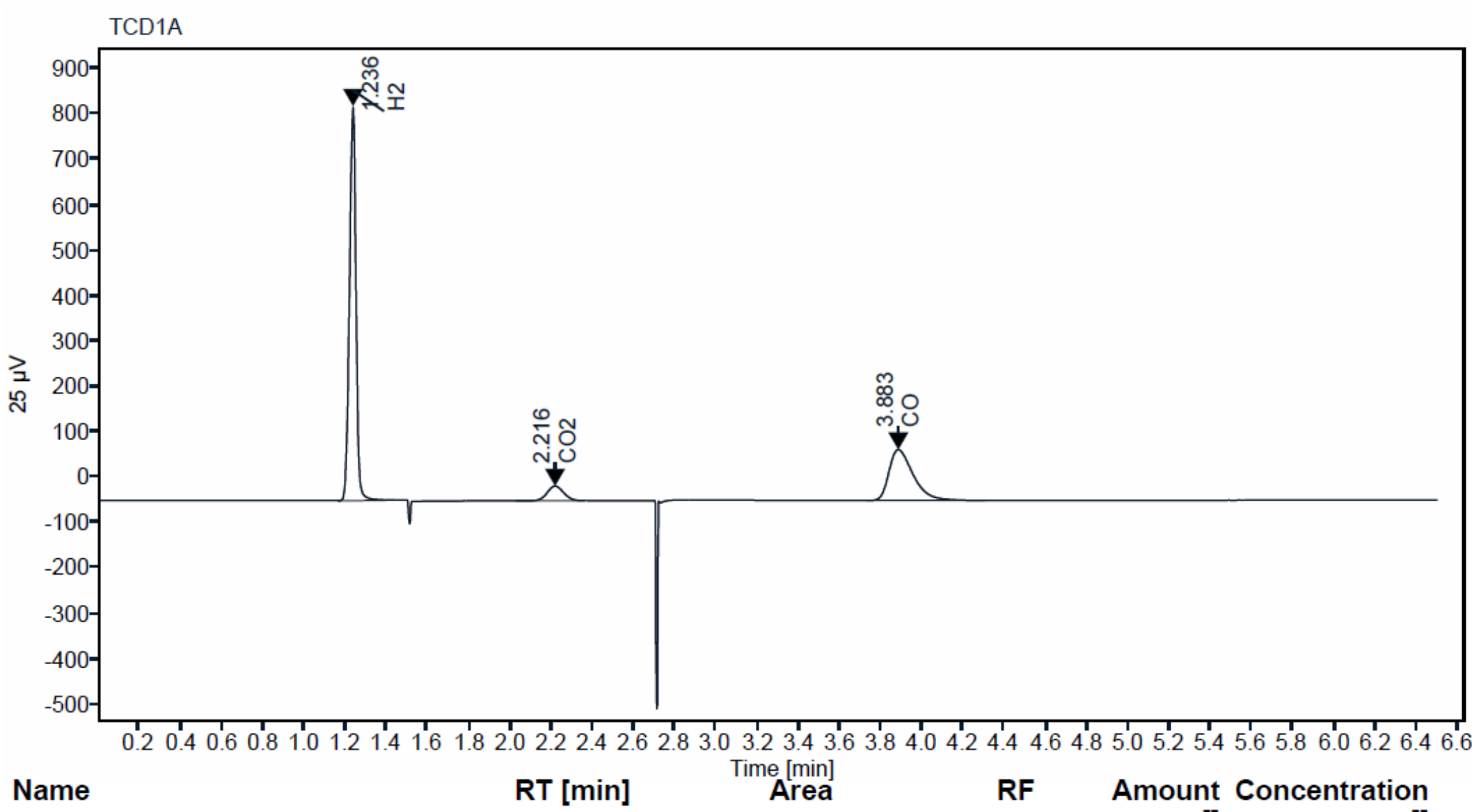

Name	RT [min]	Area	RF	Amount []	Concentration []
H2	1.236	1919.9870	455.26827	4.217	4.2173
CO2	2.216	178.3862	46.05372	3.873	38.7344
CO	3.883	911.9276	38.67660	23.578	23.5783

Figure S15: Gas chromatograph for Table 1, entry 14.

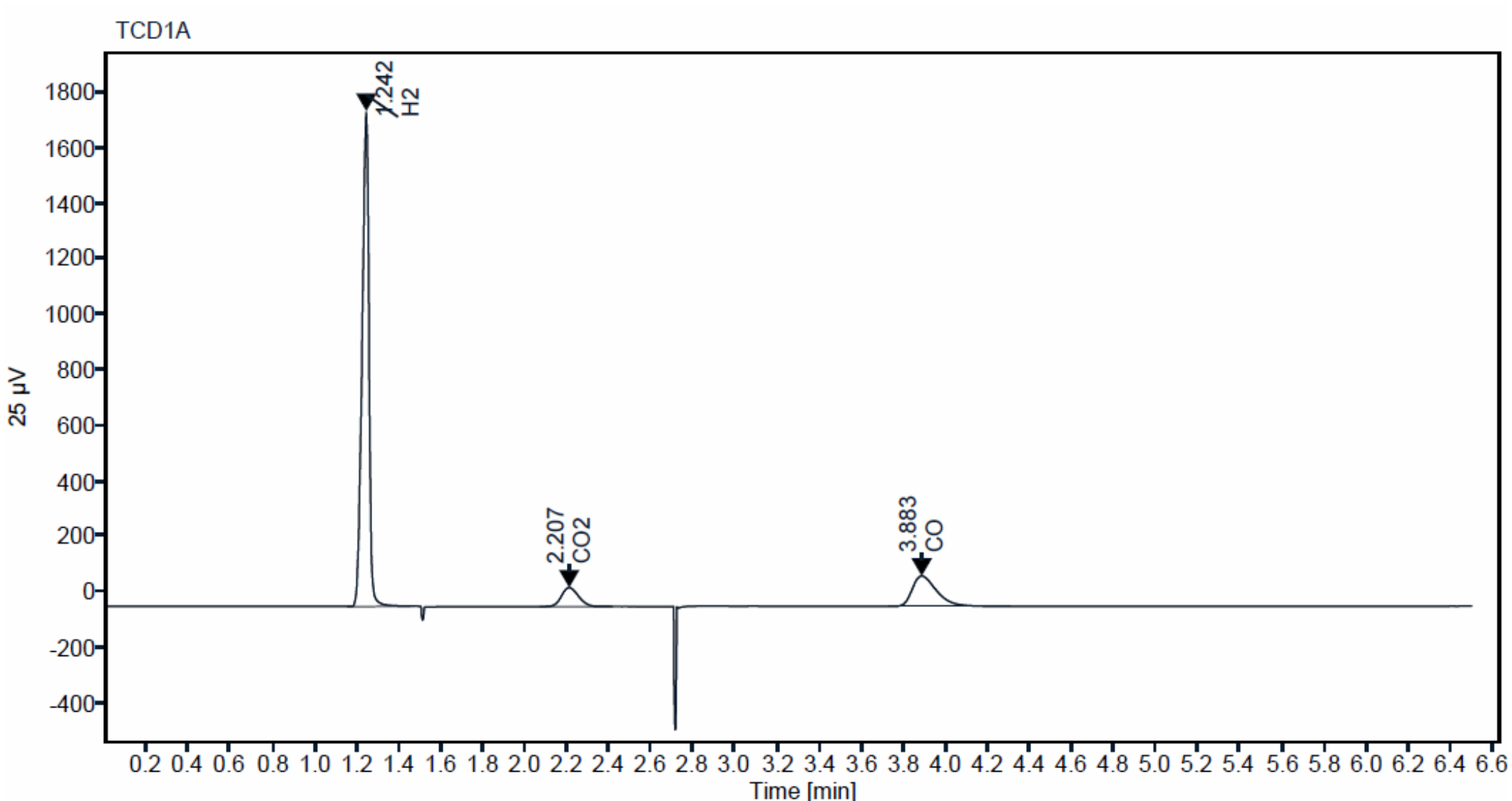

Name	RT [min]	Area	RF	Amount []	Concentration []
H2	1.242	4071.4005	455.26827	8.943	8.9429
CO2	2.207	384.0283	46.05372	8.339	83.3870
CO	3.883	892.2648	38.67660	23.070	23.0699

Figure S16: Gas chromatograph for Table 1, entry 15.

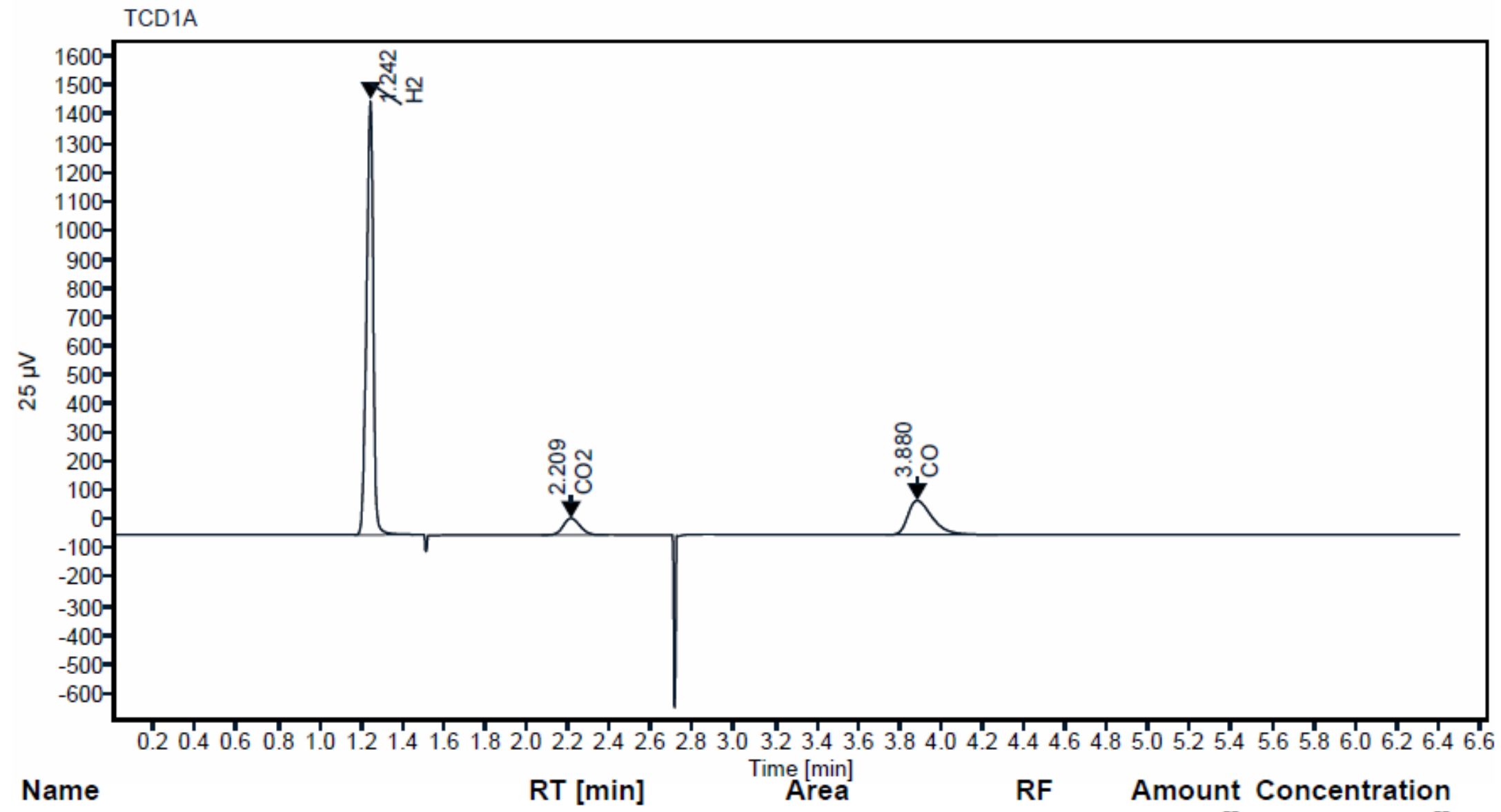

Name	RT [min]	Area	RF	Amount []	Concentration []
H2	1.242	3415.2438	455.26827	7.502	7.5016
CO2	2.209	320.1178	46.05372	6.951	69.5096
CO	3.880	966.7475	38.67660	24.996	24.9957

Figure S17: Gas chromatograph for Table 1, entry 16.

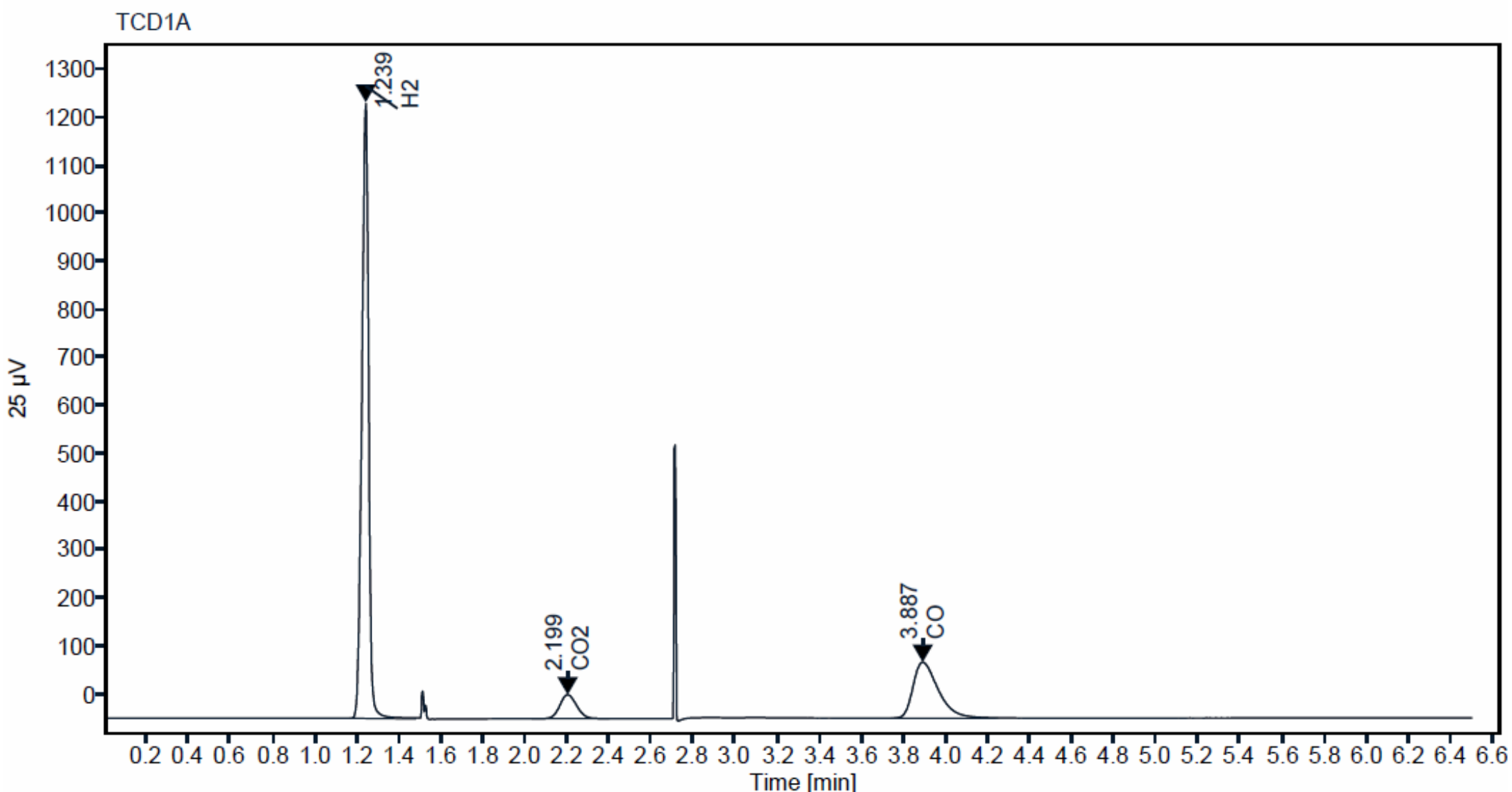

Name	RT [min]	Area	RF	Amount []	Concentration []
H2	1.239	2891.5252	455.26827	6.351	6.3513
CO2	2.199	277.1659	46.05372	6.018	60.1832
CO	3.887	968.3473	38.67660	25.037	25.0370

Figure S18: Gas chromatograph for Table 1, entry 17.

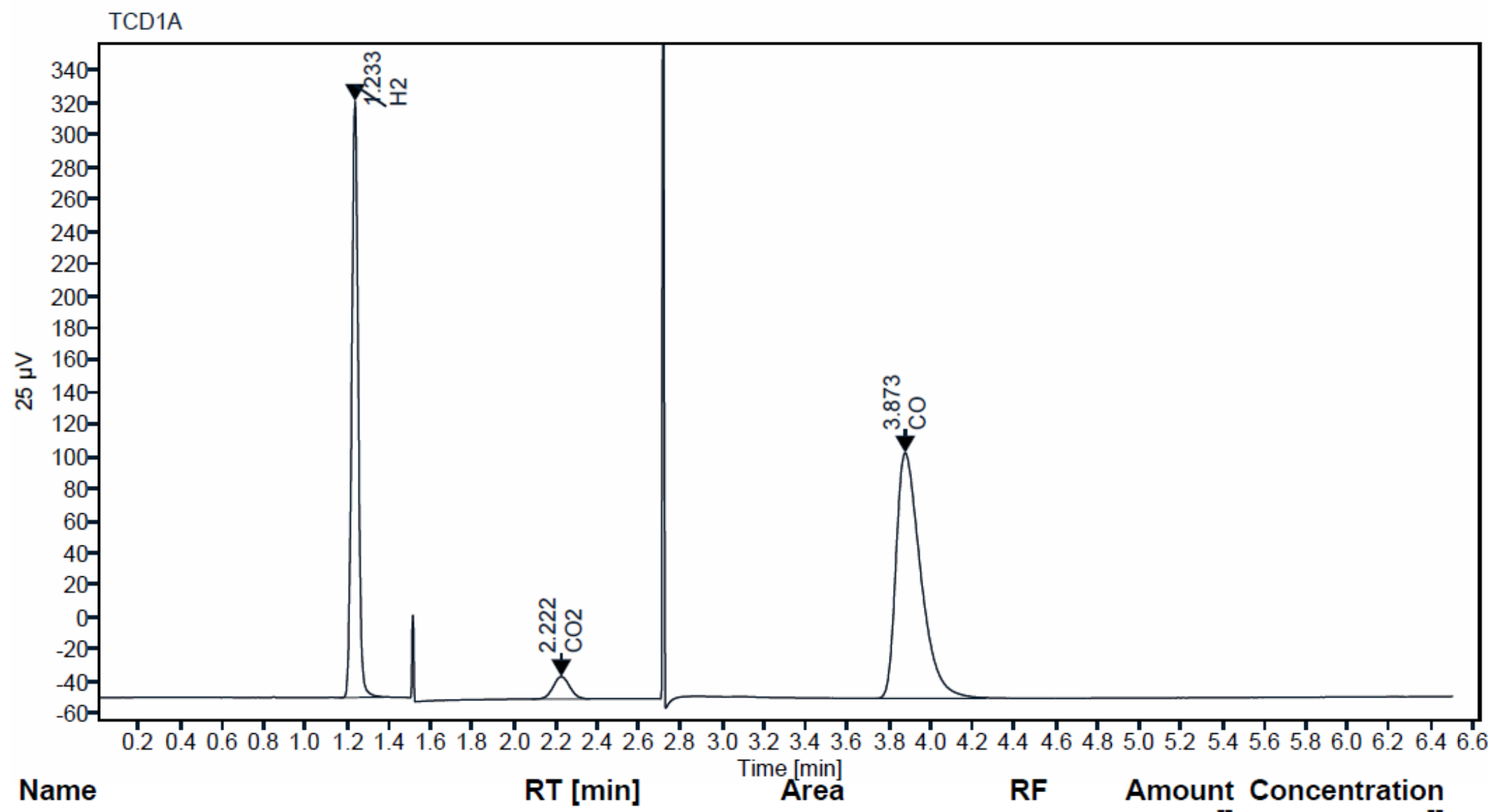

Name	RT [min]	Area	RF	Amount []	Concentration []
H2	1.233	805.2041	455.26827	1.769	1.7686
CO2	2.222	77.4948	46.05372	1.683	16.8271
CO	3.873	1256.6567	38.67660	32.491	32.4914

Figure S19: Gas chromatograph for Table 1, entry 18.

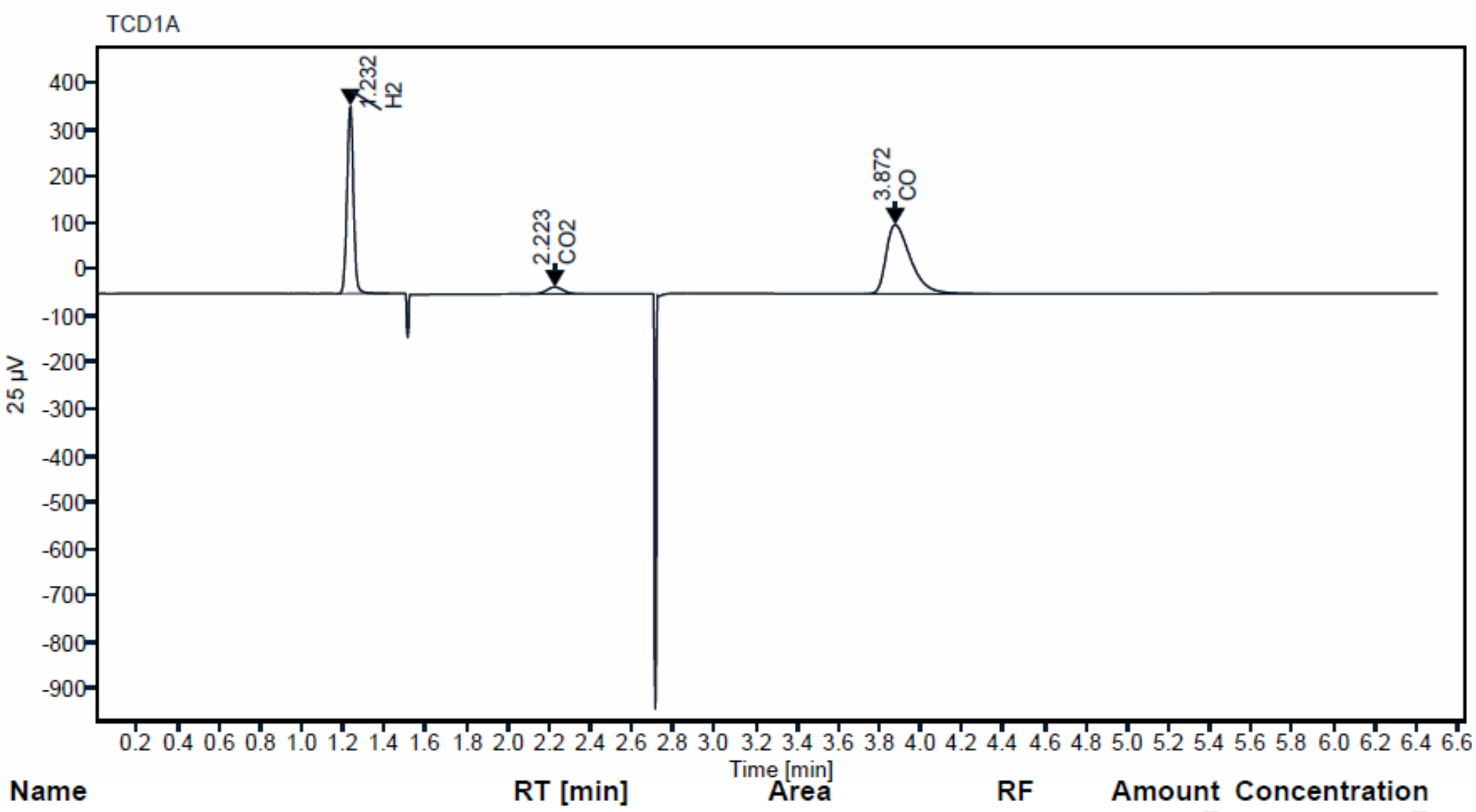

Name	RT [min]	Area	RF	Amount []	Concentration []
H2	1.232	877.5702	455.26827	1.928	1.9276
CO2	2.223	83.5929	46.05372	1.815	18.1512
CO	3.872	1221.1666	38.67660	31.574	31.5738

Figure S20: Gas chromatograph for Table 1, entry 19.

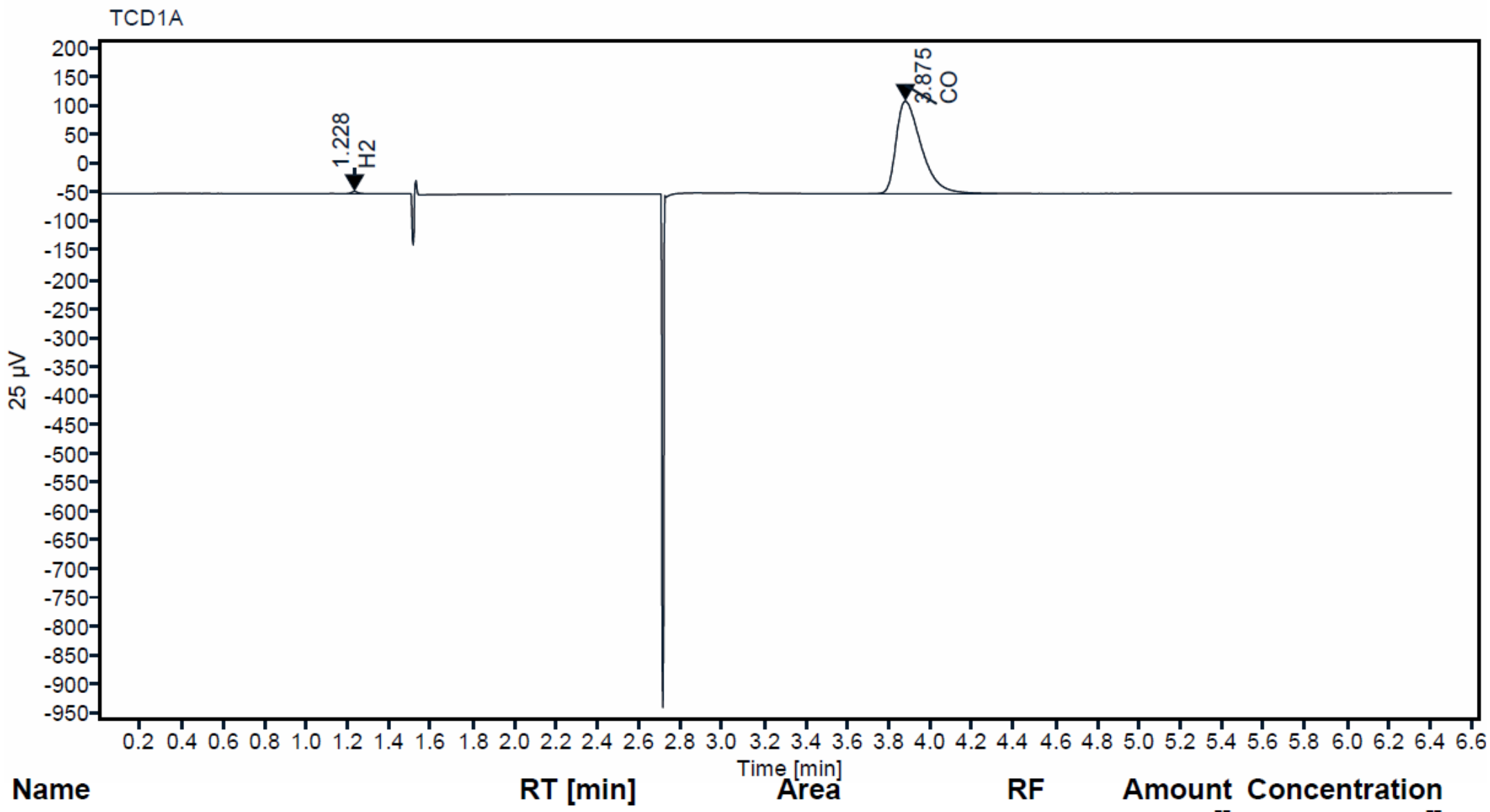

Name	RT [min]	Area	RF	Amount []	Concentration []
H2	1.228	7.7224	455.26827	0.017	0.0170
CO	3.875	1349.3248	38.67660	34.887	34.8874

Figure S21: Gas chromatograph for Table 1, entry 20.

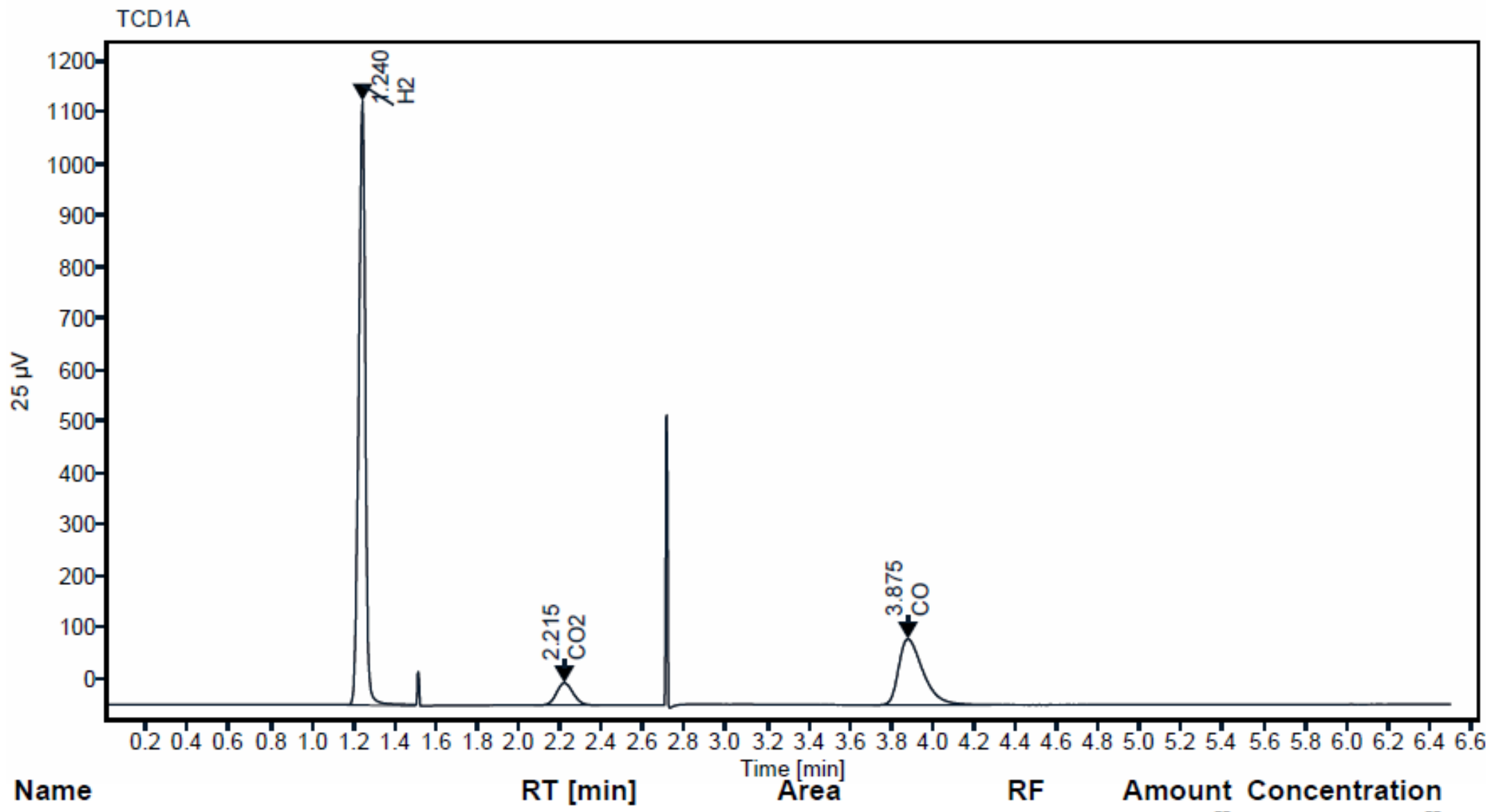

Name	RT [min]	Area	RF	Amount []	Concentration []
H2	1.240	2649.3799	455.26827	5.819	5.8194
CO2	2.215	239.4626	46.05372	5.200	51.9964
CO	3.875	1047.9919	38.67660	27.096	27.0963

Figure S22: Gas chromatograph for Table 1, entry 21.

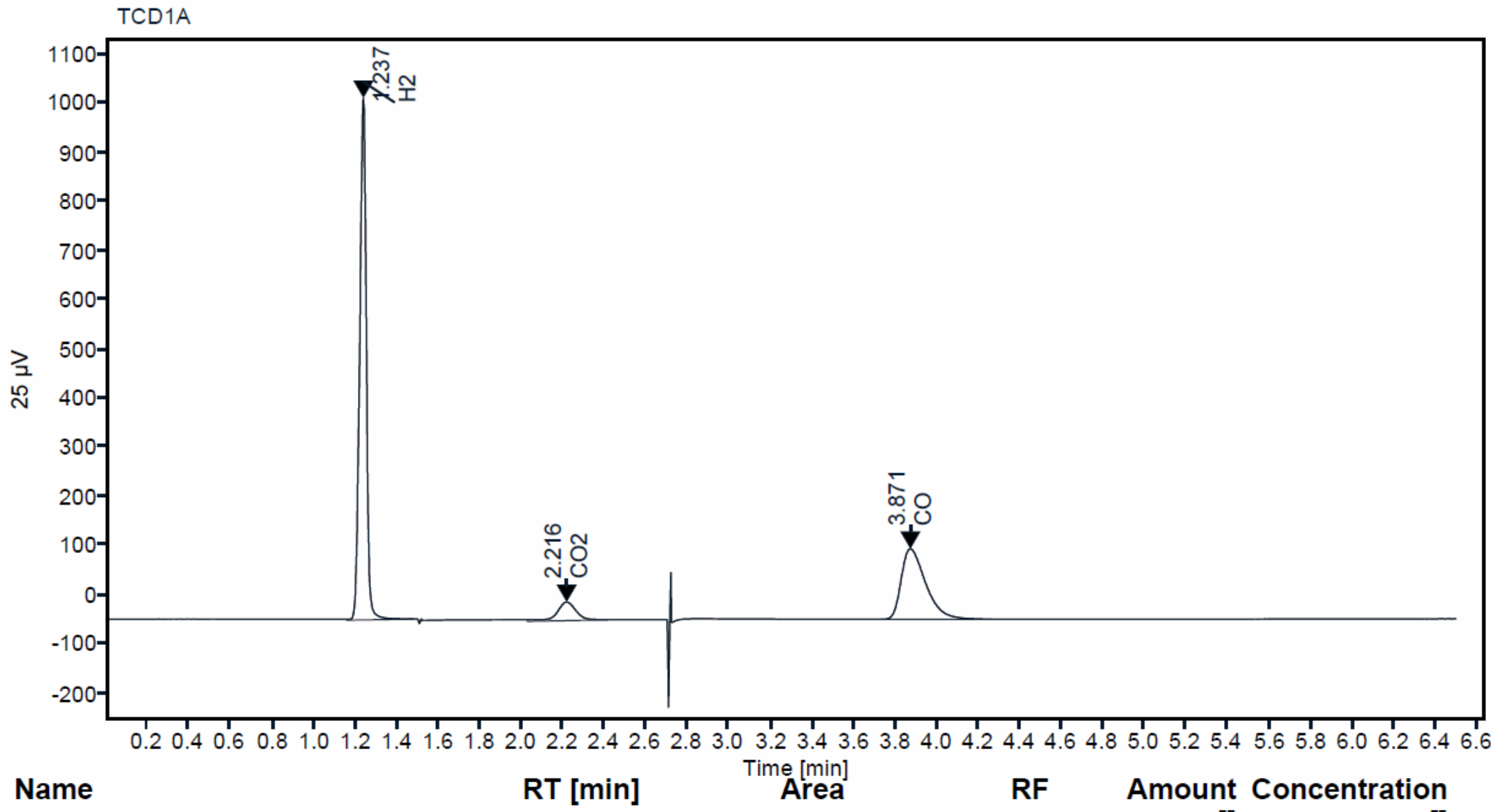

Name	RT [min]	Area	RF	Amount []	Concentration []
H2	1.237	2371.3270	455.26827	5.209	5.2086
CO2	2.216	247.7572	46.05372	5.380	53.7974
CO	3.871	1178.7312	38.67660	30.477	30.4766

Figure S23: Gas chromatograph for Table 1, entry 22.

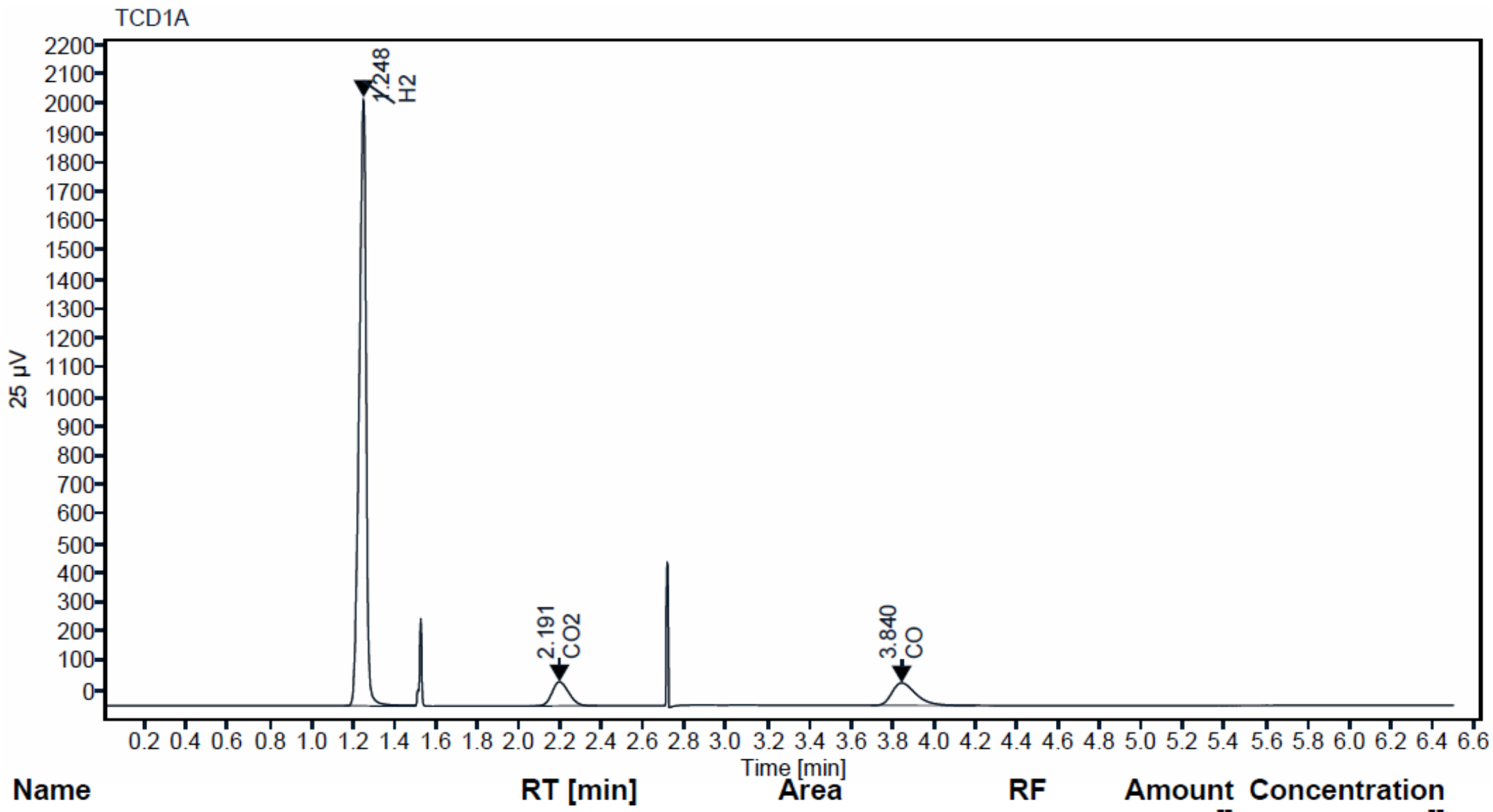

Name	RT [min]	Area	RF	Amount []	Concentration []
H2	1.248	4809.4827	455.26827	10.564	10.5641
CO2	2.191	456.1222	46.05372	9.904	99.0413
CO	3.840	623.2504	38.67660	16.114	16.1144

Figure S24: Gas chromatograph for Table 1, entry 23.

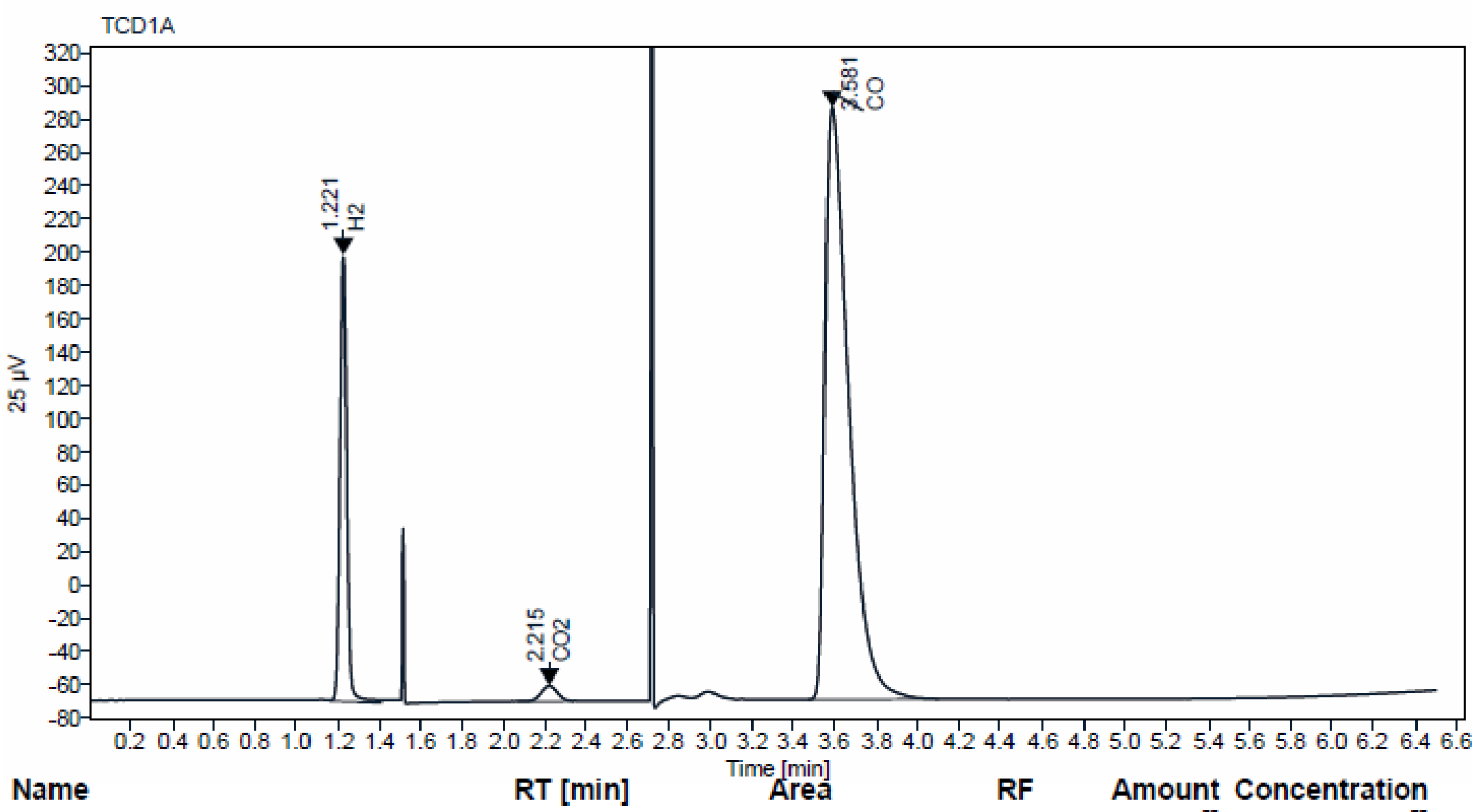

Name	RT [min]	Area	RF	Amount []	Concentration []
H2	1.221	596.5997	455.26827	1.310	1.3104
CO2	2.215	54.2238	46.05372	1.177	11.7740
CO	3.581	2856.6945	38.67660	73.861	73.8610

Figure S25: Gas chromatograph for Table 1, entry 24 (peaks at 2.9 and 3.3 min are due to trace air induced during gas sampling).

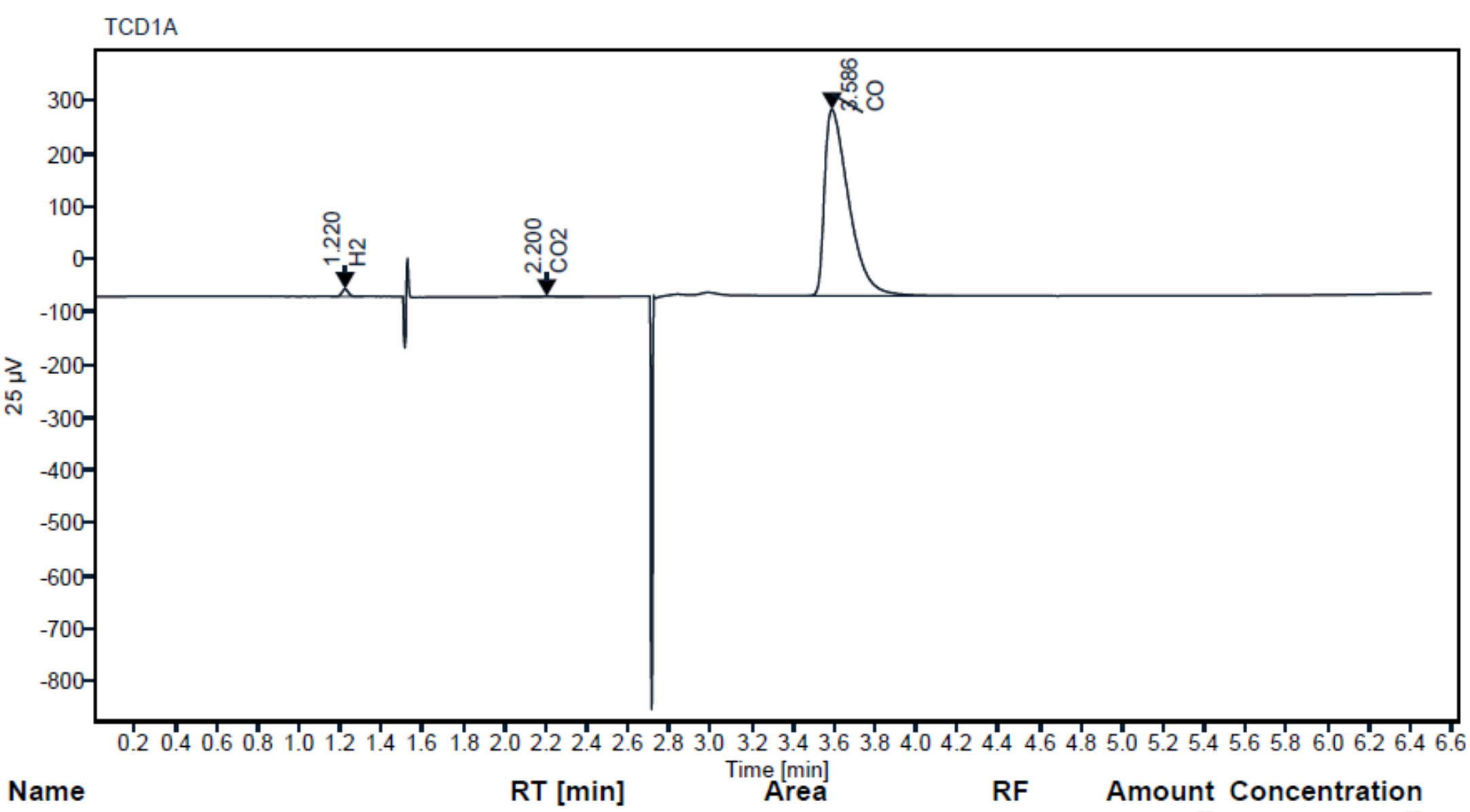

Name	RT [min]	Area	RF	Amount []	Concentration []
H2	1.220	30.6688	455.26827	0.067	0.0674
CO2	2.200	3.5008	46.05372	0.076	0.7601
CO	3.586	2881.1488	38.67660	74.493	74.4933

Figure S26: Gas chromatograph for Table 1, entry 25.

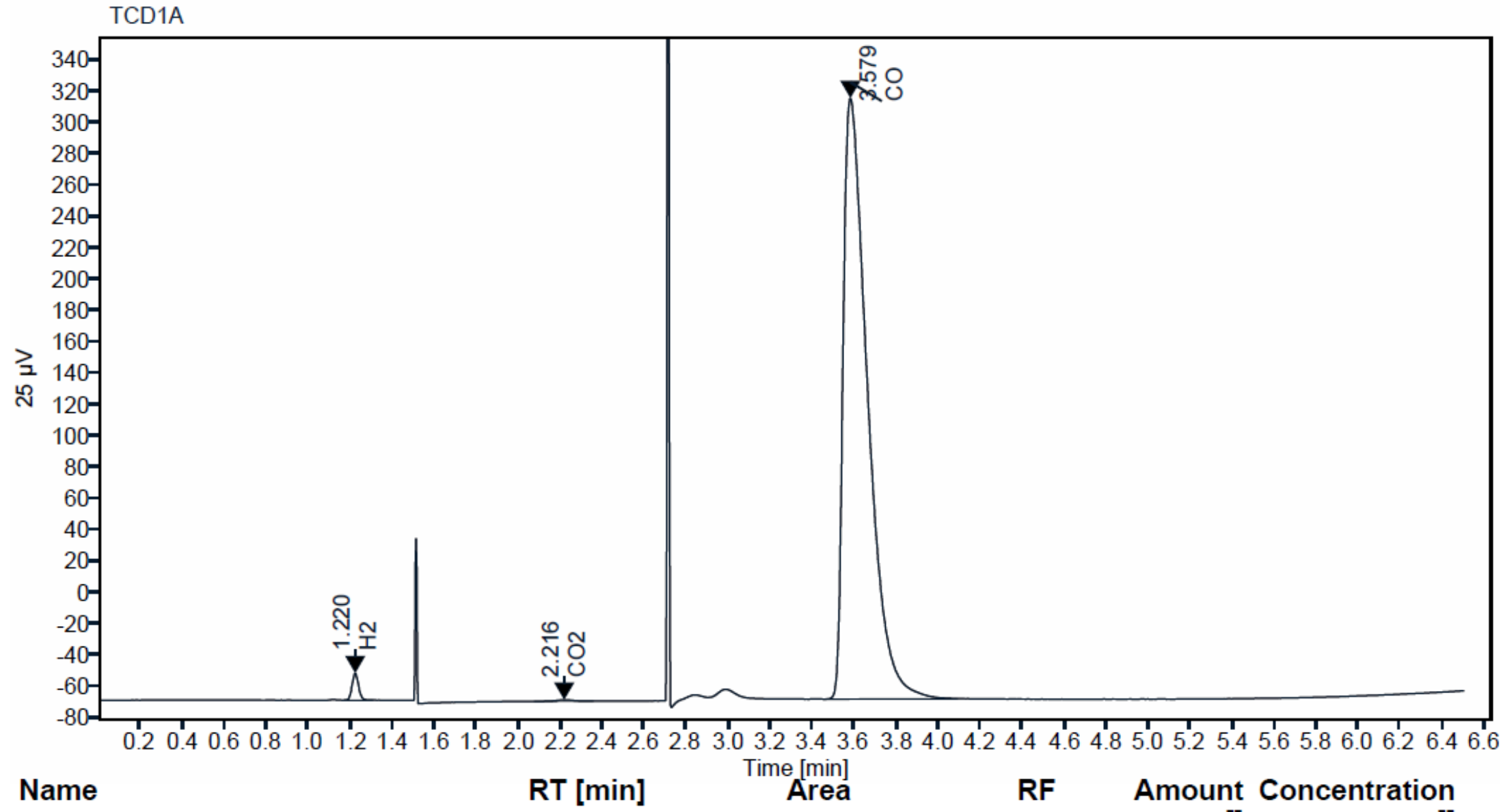

Name	RT [min]	Area	RF	Amount []	Concentration []
H2	1.220	37.1732	455.26827	0.082	0.0817
CO2	2.216	4.5883	46.05372	0.100	0.9963
CO	3.579	3098.4272	38.67660	80.111	80.1112

Figure S27: Gas chromatograph for Table 1, entry 26 (peaks at 2.9 and 3.3 min are due to trace air induced during gas sampling).

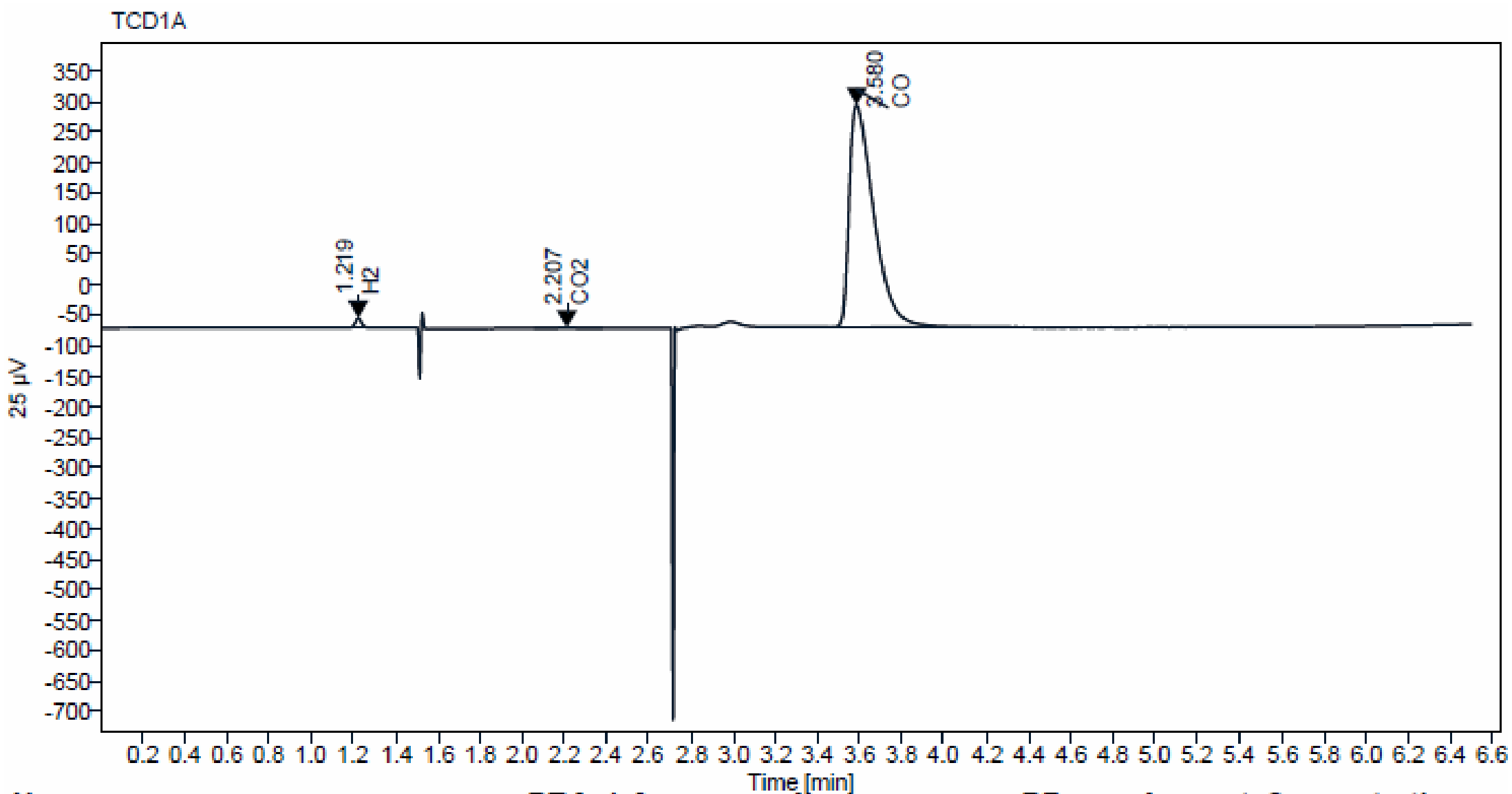

Name	RT [min]	Area	RF	Amount []	Concentration []
H2	1.219	34.1918	455.26827	0.075	0.0751
CO2	2.207	6.0950	46.05372	0.132	1.3235
CO	3.580	2967.4890	38.67660	76.726	76.7257

Figure S28: Gas chromatograph for Table 1, entry 27 (peaks at 2.9 and 3.3 min are due to trace air induced during gas sampling).

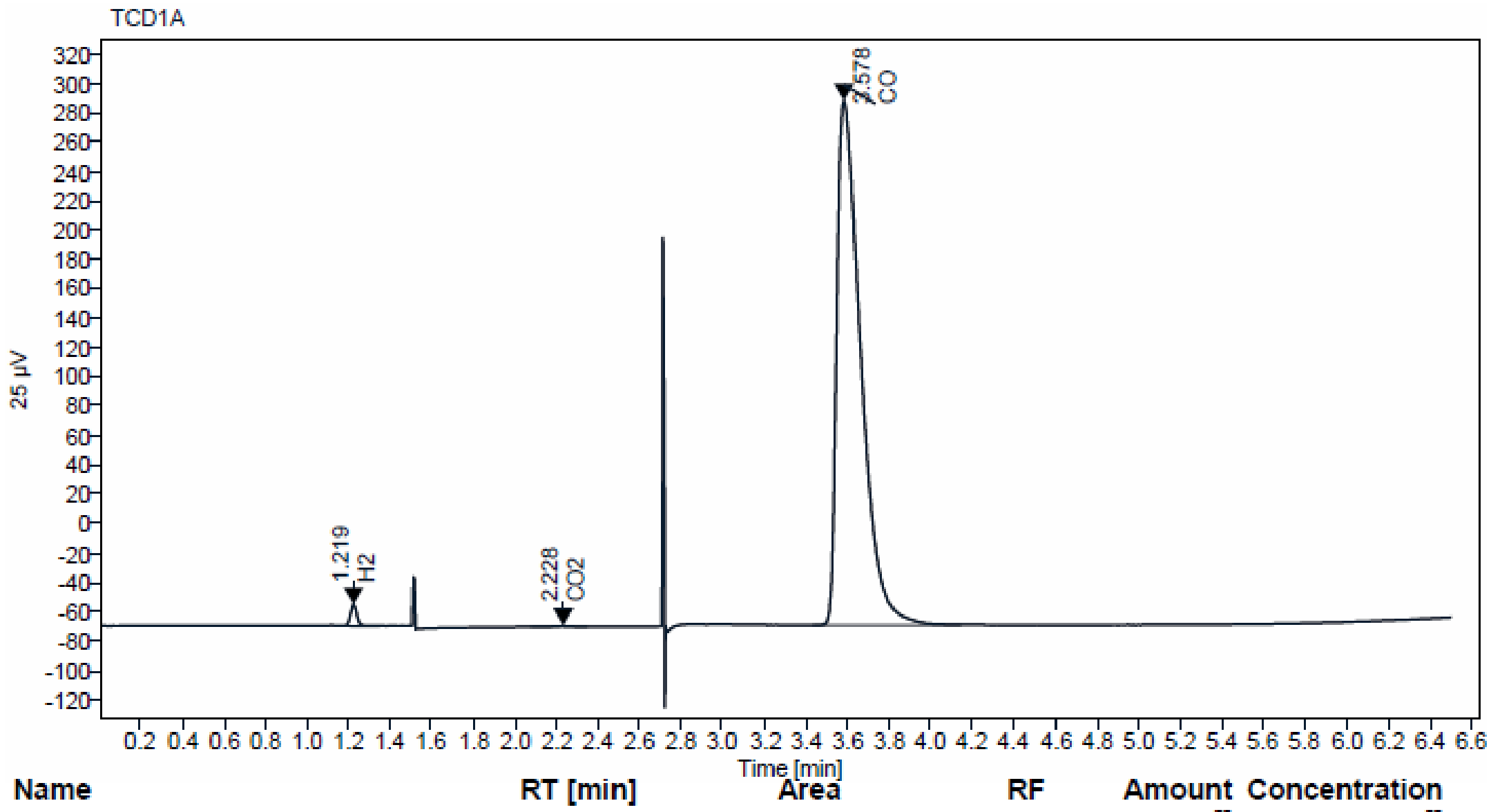

Name	RT [min]	Area	RF	Amount []	Concentration []
H2	1.219	31.2639	455.26827	0.069	0.0687
CO2	2.228	3.8127	46.05372	0.083	0.8279
CO	3.578	2839.6426	38.67660	73.420	73.4202

Figure S29: Gas chromatograph for Table 1, entry 28.

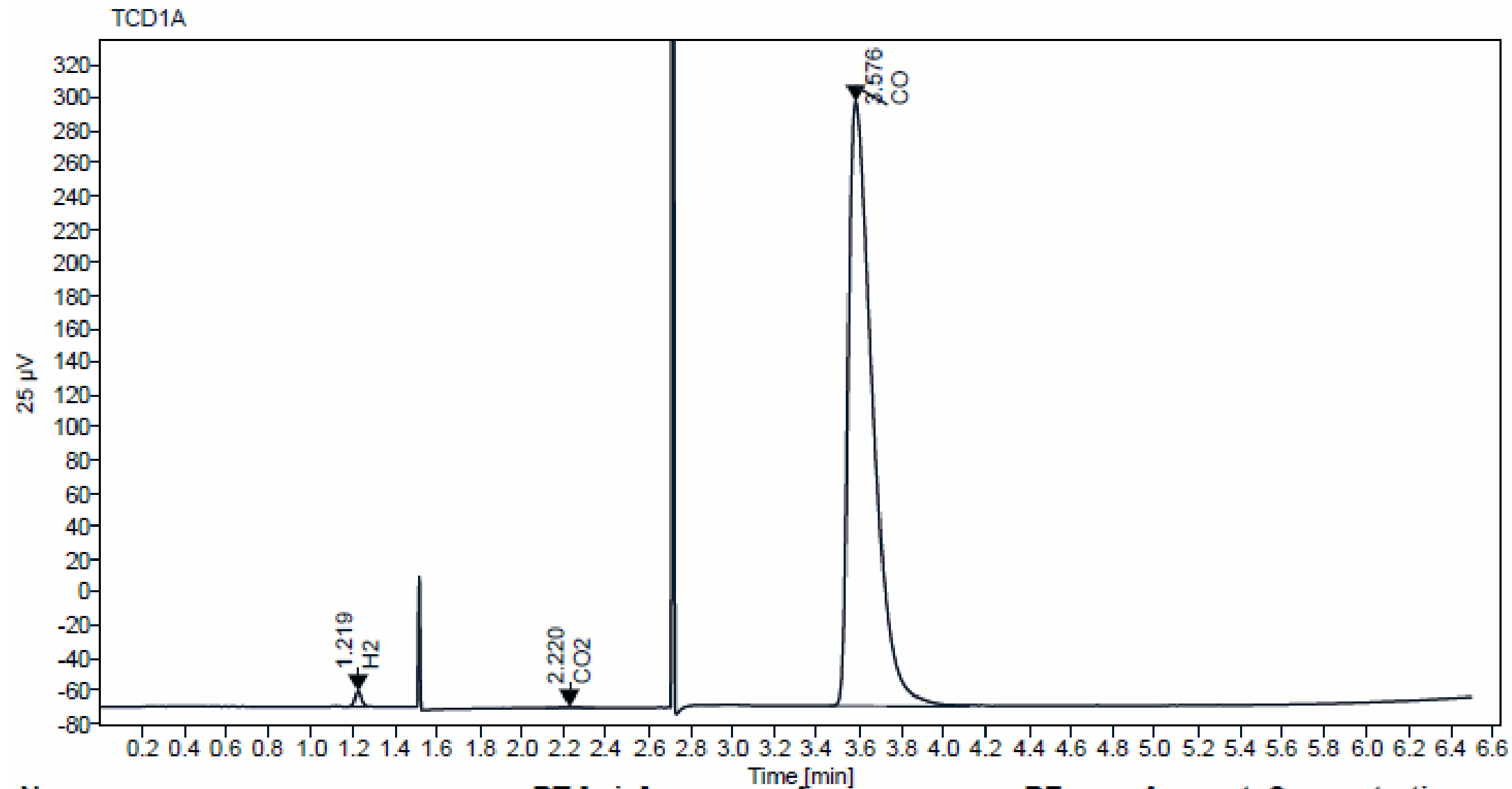

Name	RT [min]	Area	RF	Amount []	Concentration []
H2	1.219	19.0149	455.26827	0.042	0.0418
CO2	2.220	2.7805	46.05372	0.060	0.6038
CO	3.576	2922.9028	38.67660	75.573	75.5729

Figure S30: Gas chromatograph for Table 1, entry 29.

3.2. GC-TCD data for H-flask reactions

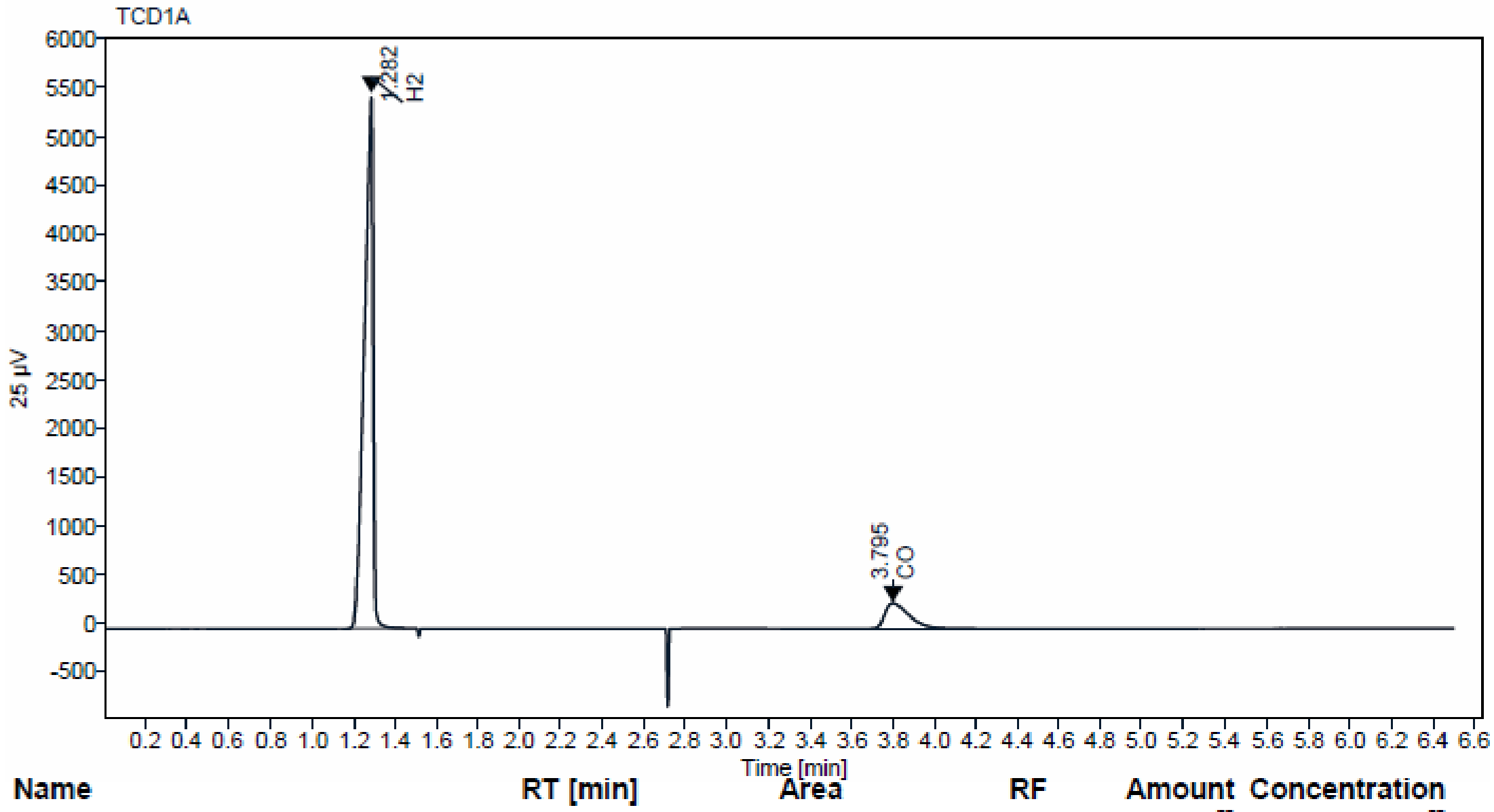

Name	RT [min]	Area	RF	Amount []	Concentration []
H2	1.282	16110.8897	455.26827	35.388	35.3877
CO	3.795	2170.9503	38.67660	56.131	56.1308

Figure S31: Gas chromatograph for Table 2, entry 1.

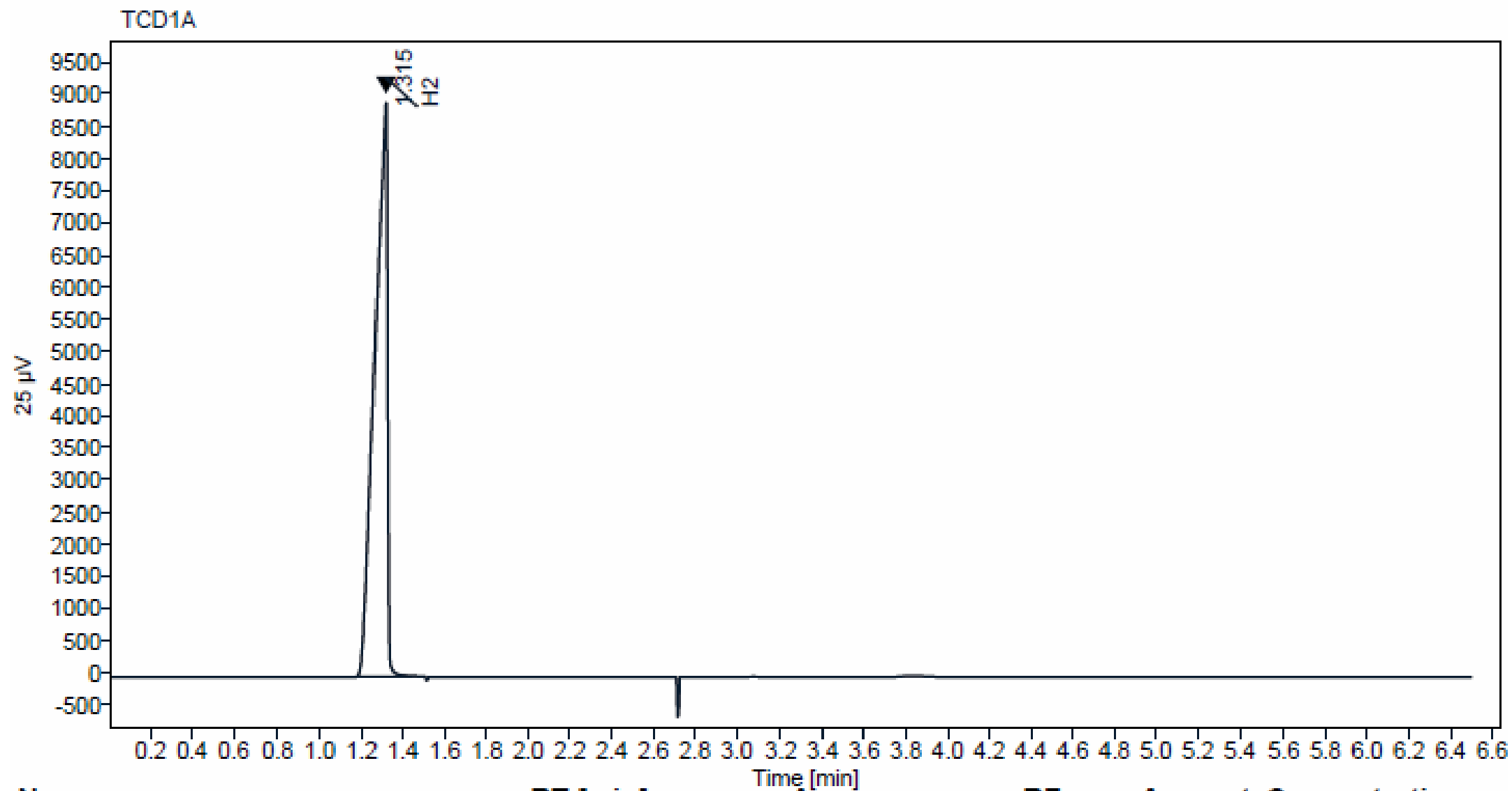

Name	RT [min]	Area	RF	Amount []	Concentration []
H2	1.315	36993.1352	455.26827	81.256	81.2557

Figure S32: Gas chromatograph for Table 2, entry 2.

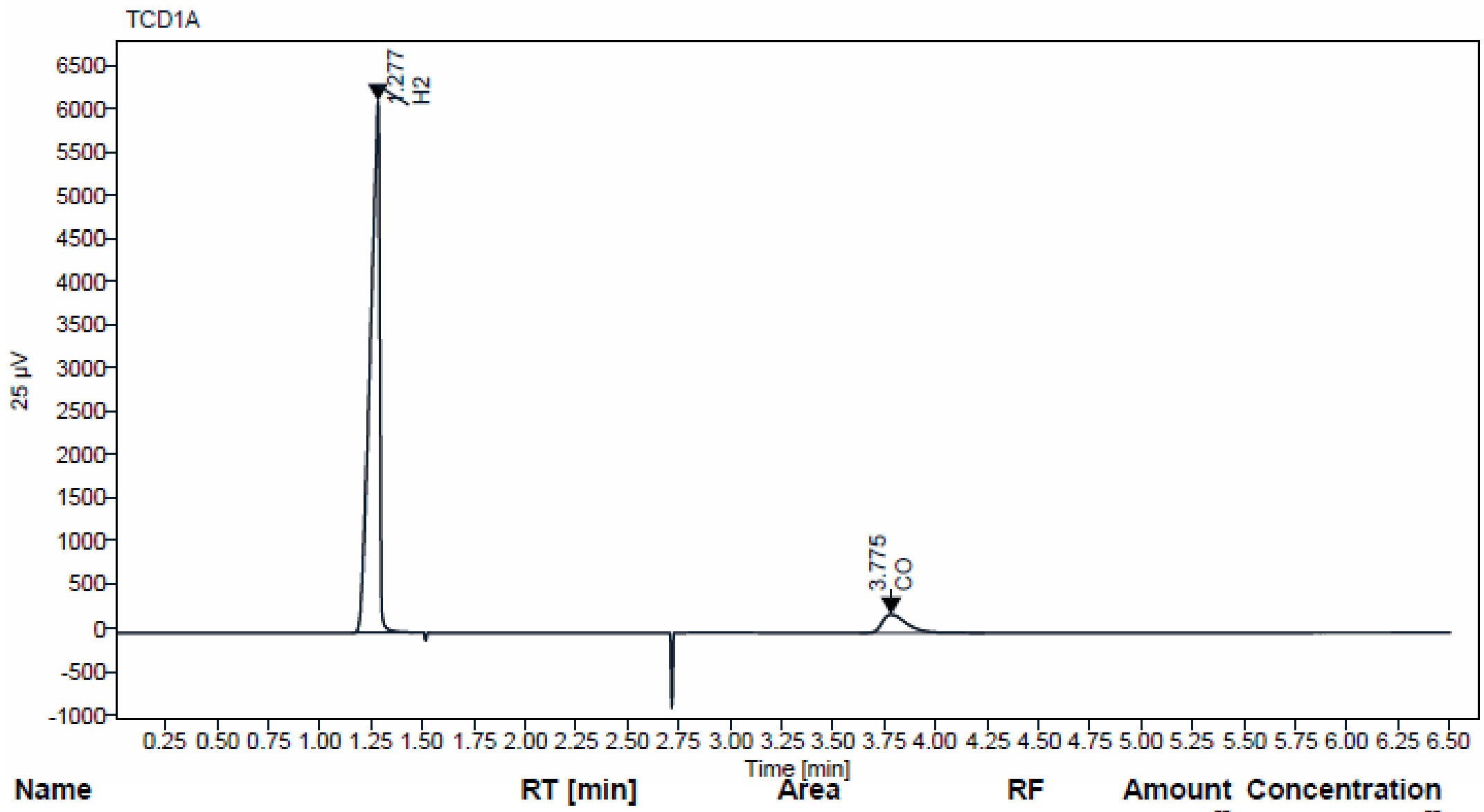

Name	RT [min]	Area	RF	Amount []	Concentration []
H2	1.277	19302.1869	455.26827	42.397	42.3974
CO	3.775	1767.1325	38.67660	45.690	45.6900

Figure S33: Gas chromatograph for Table 2, entry 3.

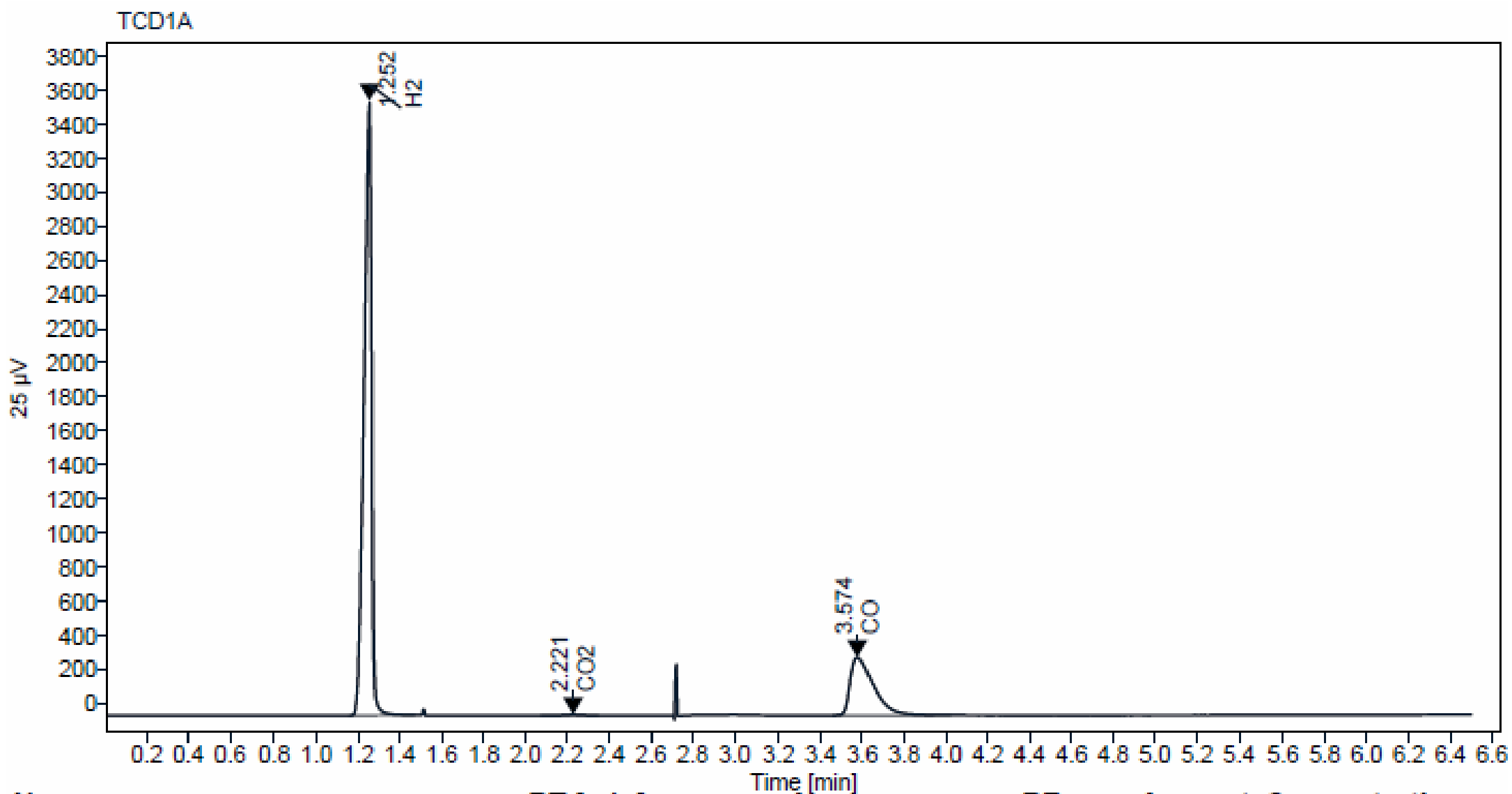

Name	RT [min]	Area	RF	Amount []	Concentration []
H2	1.252	9066.8096	455.26827	19.915	19.9153
CO2	2.221	26.2833			
CO	3.574	2648.8958	38.67660	68.488	68.4883

Figure S34: Gas chromatograph for Table 2, entry 4.

3.3. GC-TCD data for catalyst recycling studies

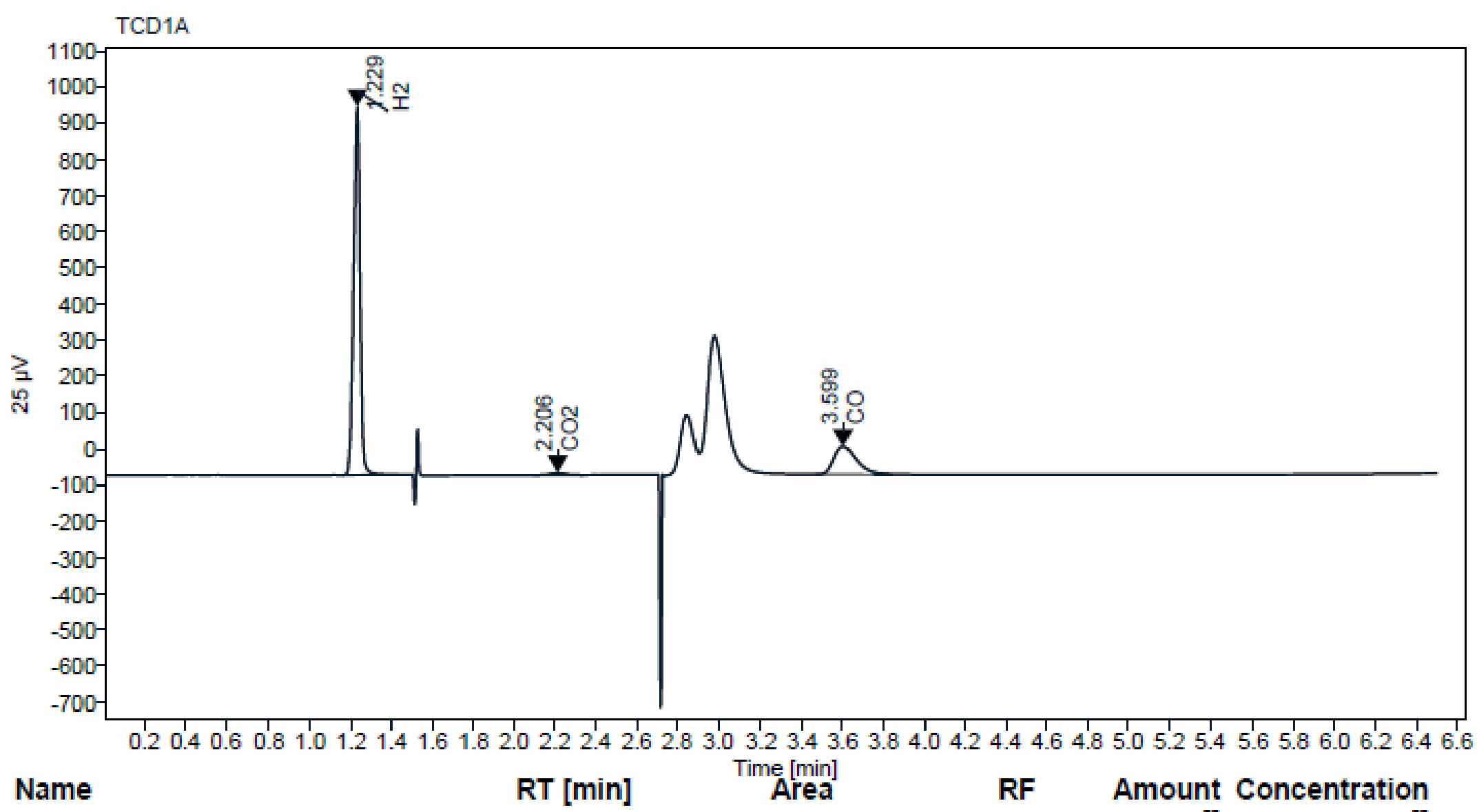

Name	RT [min]	Area	RF	Amount []	Concentration []
H2	1.229	2279.7955	455.26827	5.008	5.0076
CO2	2.206	34.3135	34.3135	46.05372	0.745
CO	3.599	588.3432	38.67660	15.212	15.2119

Figure S35: Gas chromatograph for Table 4, cycle 1 (peaks at 2.9 and 3.3 min are due to trace air induced during gas sampling).

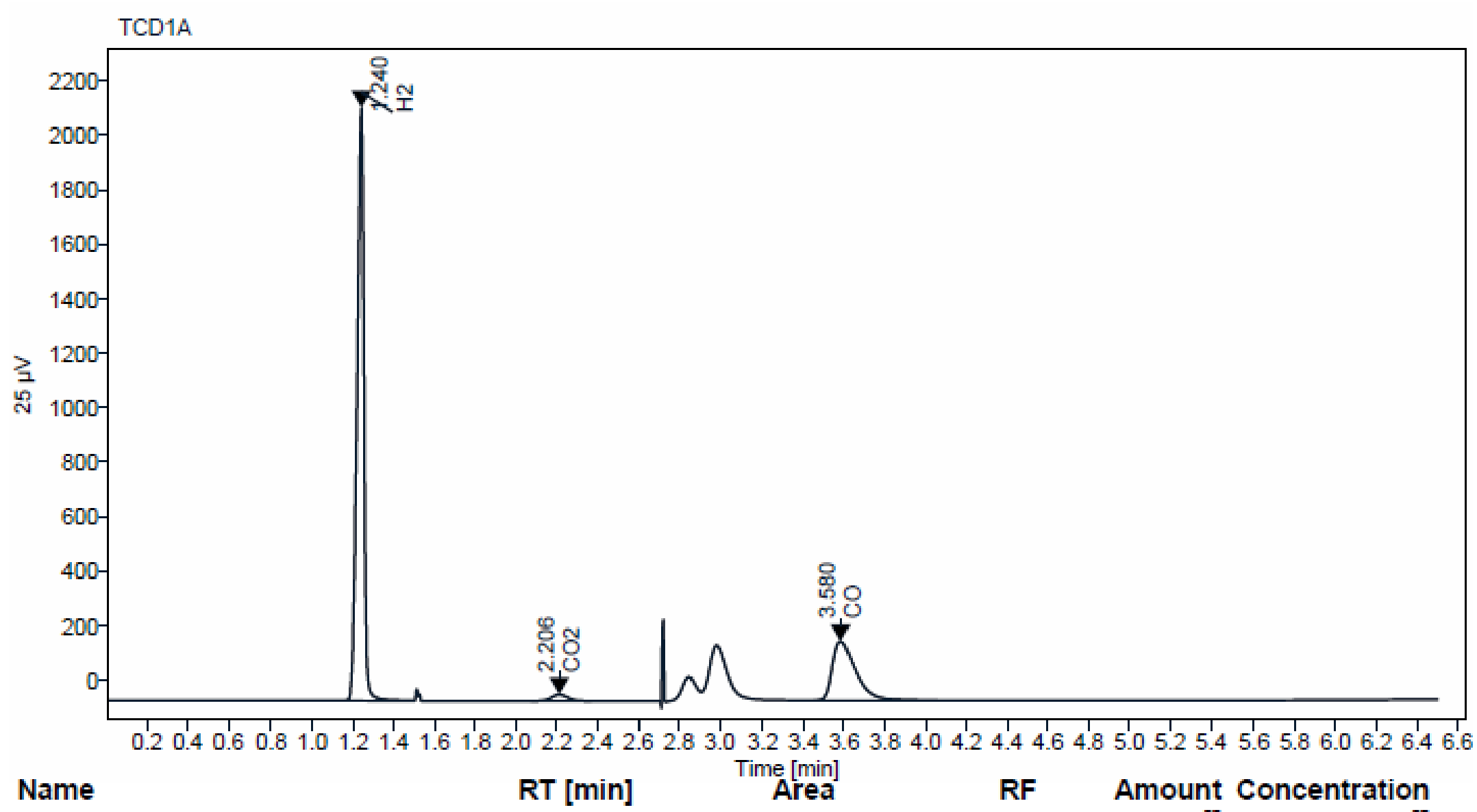

Name	RT [min]	Area	RF	Amount []	Concentration []
H2	1.240	5095.6360	455.26827	11.193	11.1926
CO2	2.206	126.4455	126.4455	46.05372	2.746
CO	3.580	1685.9504	38.67660	43.591	43.5910

Figure S36: Gas chromatograph for Table 4, cycle 2 (peaks at 2.9 and 3.3 min are due to trace air induced during gas sampling).

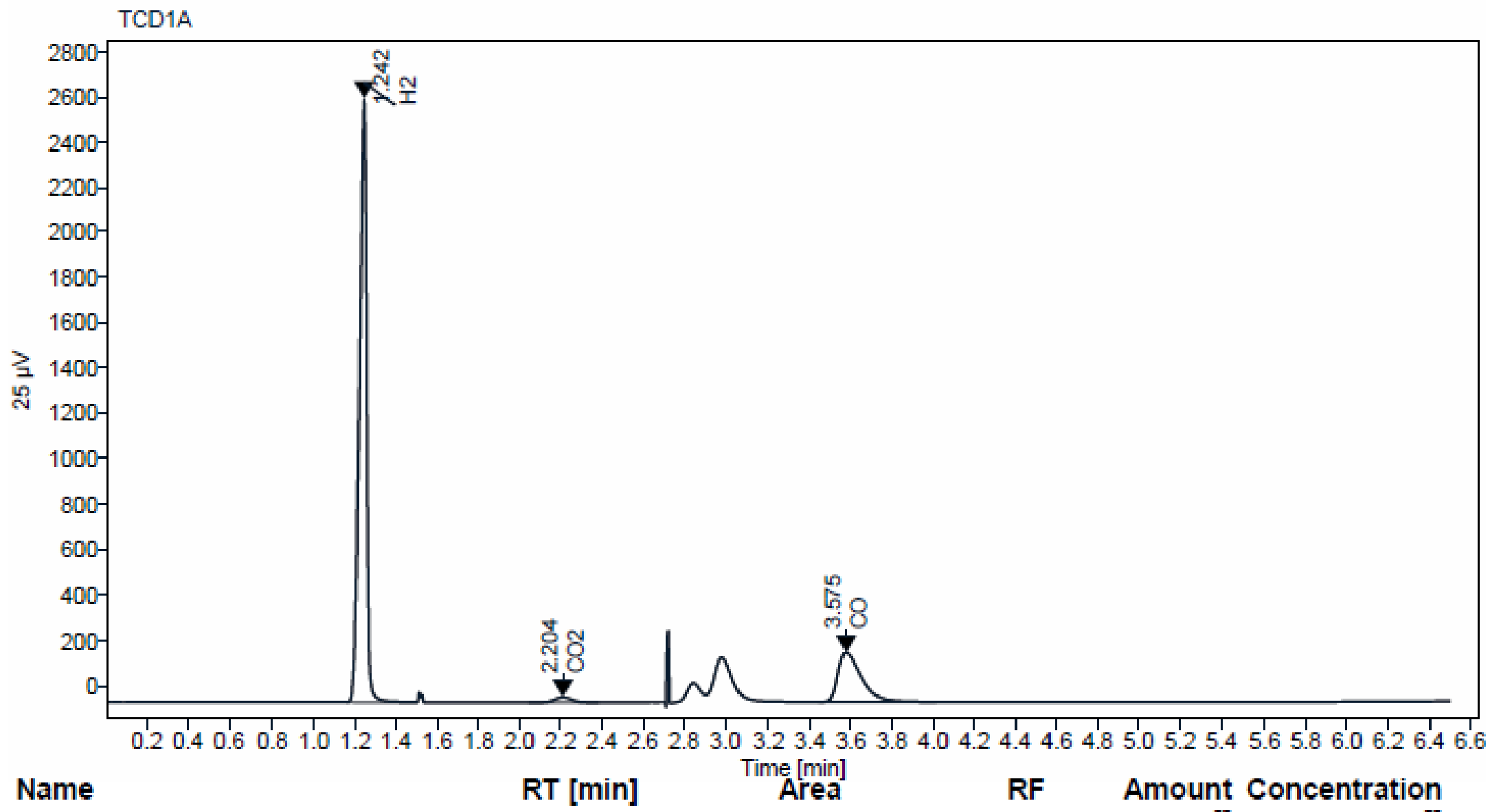

Name	RT [min]	Area	RF	Amount []	Concentration []
H2	1.242	6388.2752	455.26827	14.032	14.0319
CO2	2.204	119.5060	119.5060	46.05372	2.595
CO	3.575	1682.7910	38.67660	43.509	43.5093

Figure S37: Gas chromatograph for Table 4, cycle 3 (peaks at 2.9 and 3.3 min are due to trace air induced during gas sampling).

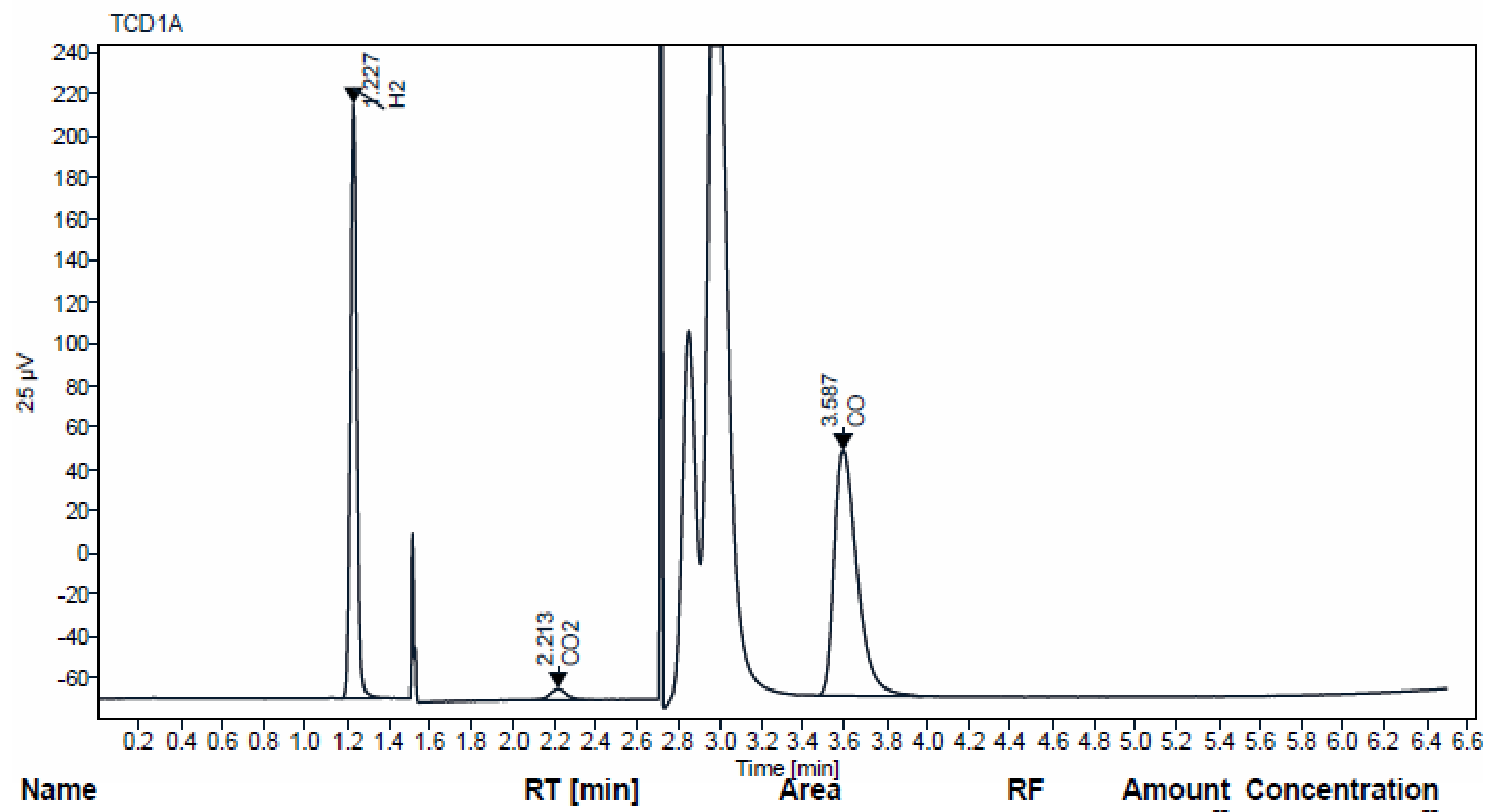

Name	RT [min]	Area	RF	Amount []	Concentration []
H2	1.227	628.4054	455.26827	1.380	1.3803
CO2	2.213	28.7318	28.7318	46.05372	0.624
CO	3.587	893.8805	38.67660	23.112	23.1117

Figure S38: Gas chromatograph for Table 4, cycle 4 (peaks at 2.9 and 3.3 min are due to trace air induced during gas sampling).

3.4. GC-TCD data for NMR scale reactions

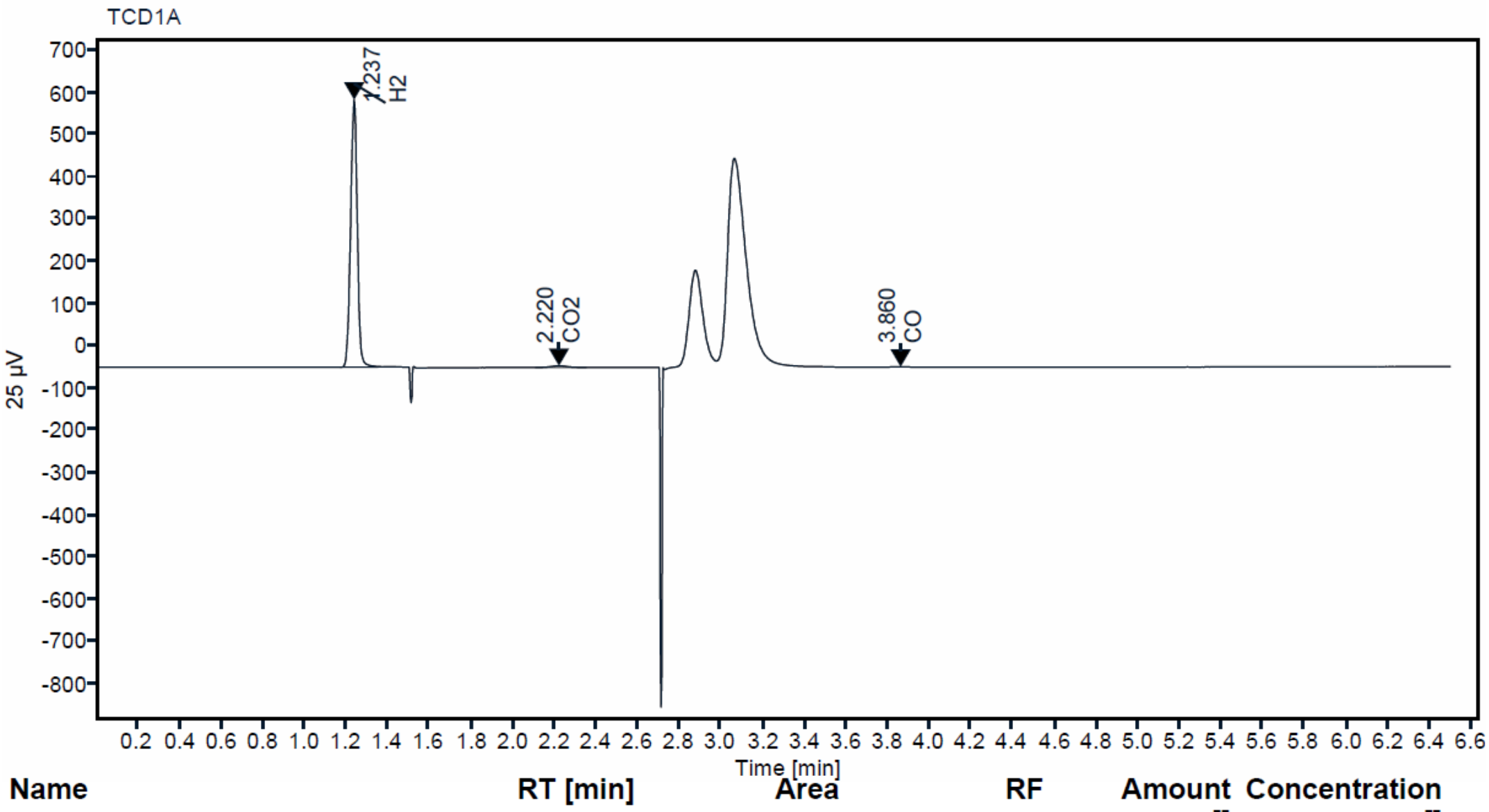

Name	RT [min]	Area	RF	Amount []	Concentration []
H2	1.237	1410.2870	455.26827	3.098	3.0977
CO2	2.220	21.3036	46.05372	0.463	4.6258
CO	3.860	5.7691	38.67660	0.149	0.1492

Figure S39: Gas chromatograph for NMR scale reaction (peaks at 2.9 and 3.3 min are due to trace air induced during gas sampling).

4. NMR data

4.1. Representative NMR data for WGS reactions

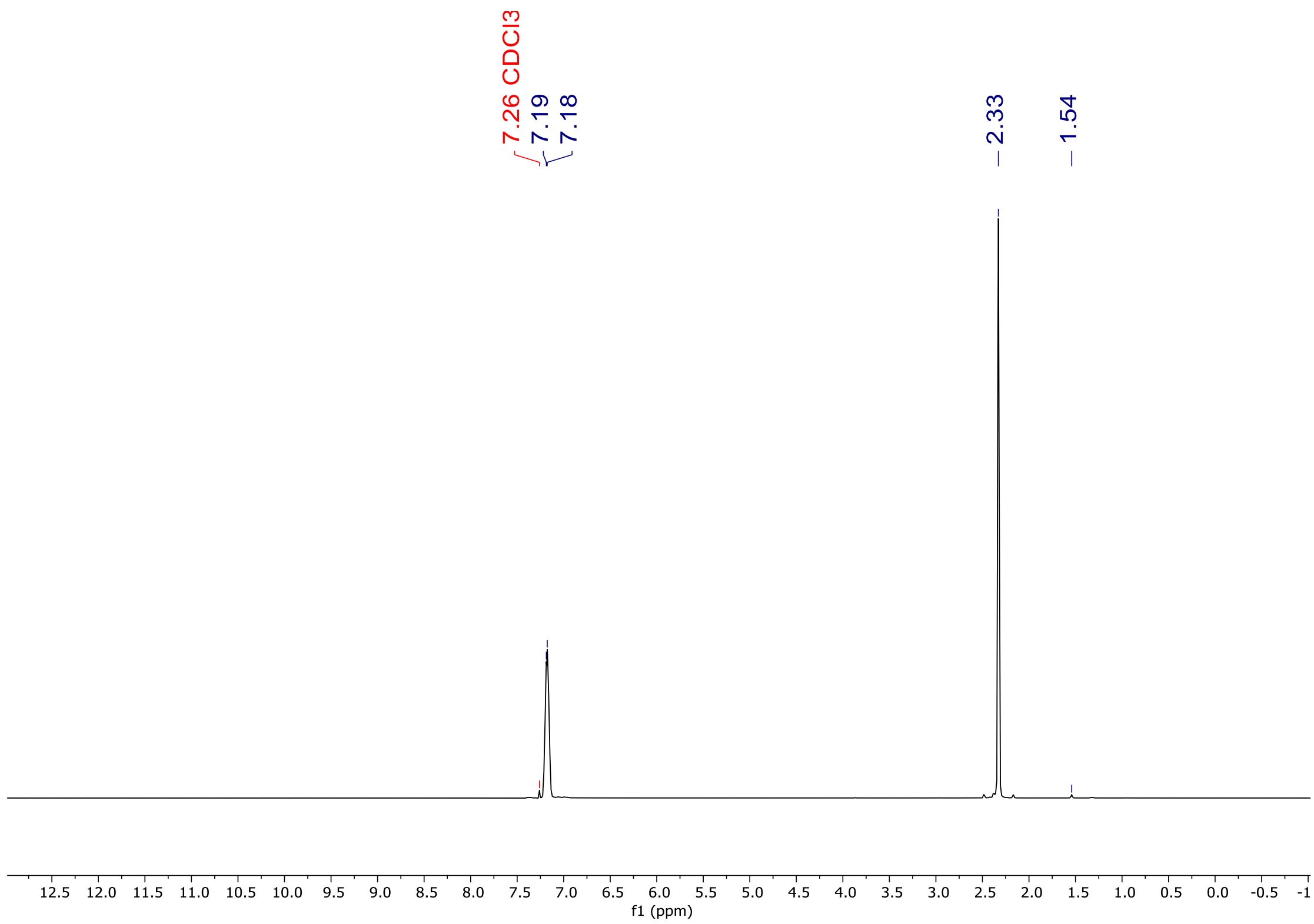

Figure S40: Representative ^{1}H NMR ($CDCl_3$, 400 MHz, 298 K) for Table 1 WGS optimisation studies.

4.2. Representative NMR data for aqueous side of H-flask reactions

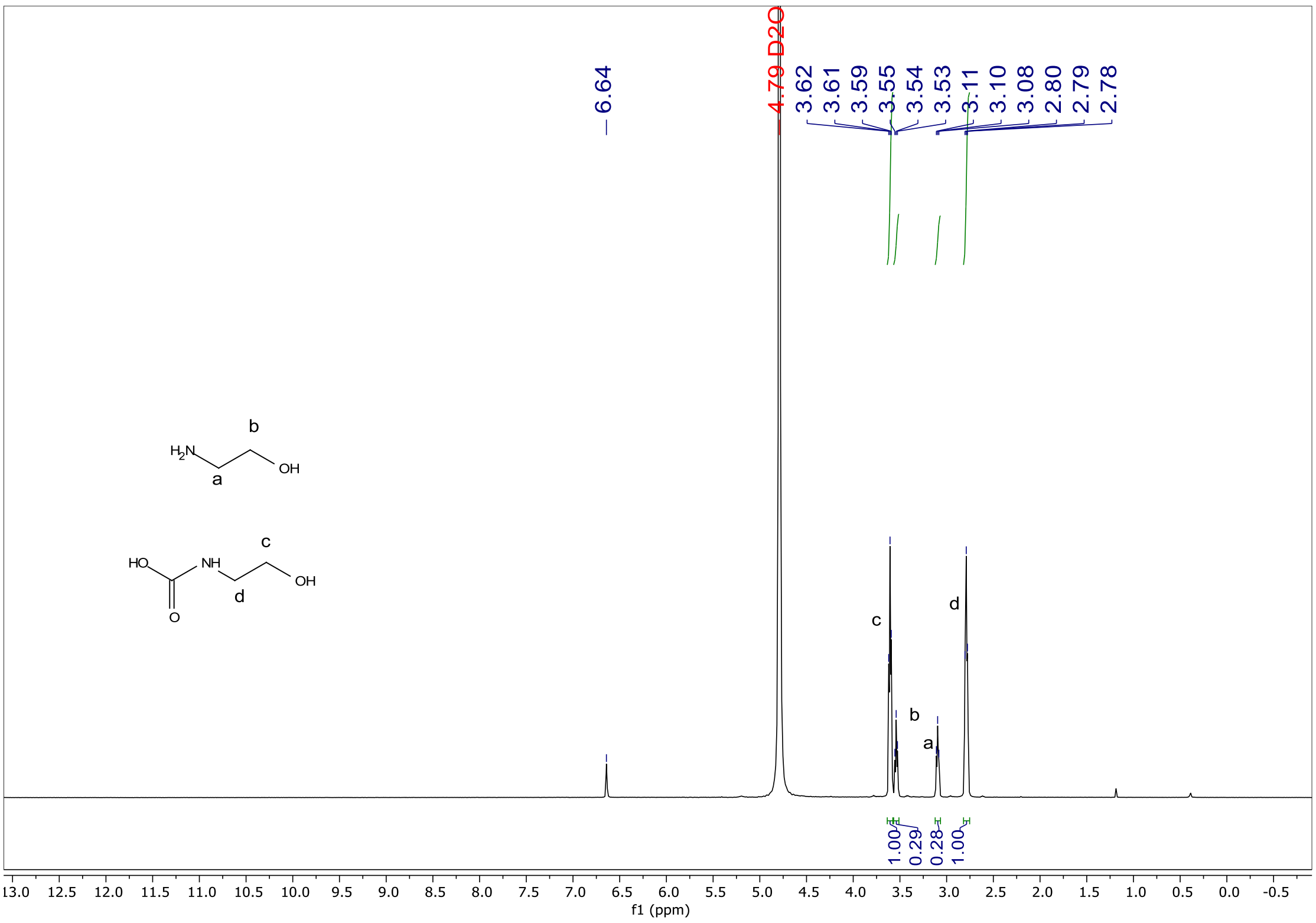

Figure S41: Representative ^{1}H NMR ($CDCl_3$, 400 MHz, 298 K) for aqueous phase (side B) for Table 2 H-flask reactions.

4.3. NMR data for transfer hydrogenation reactions

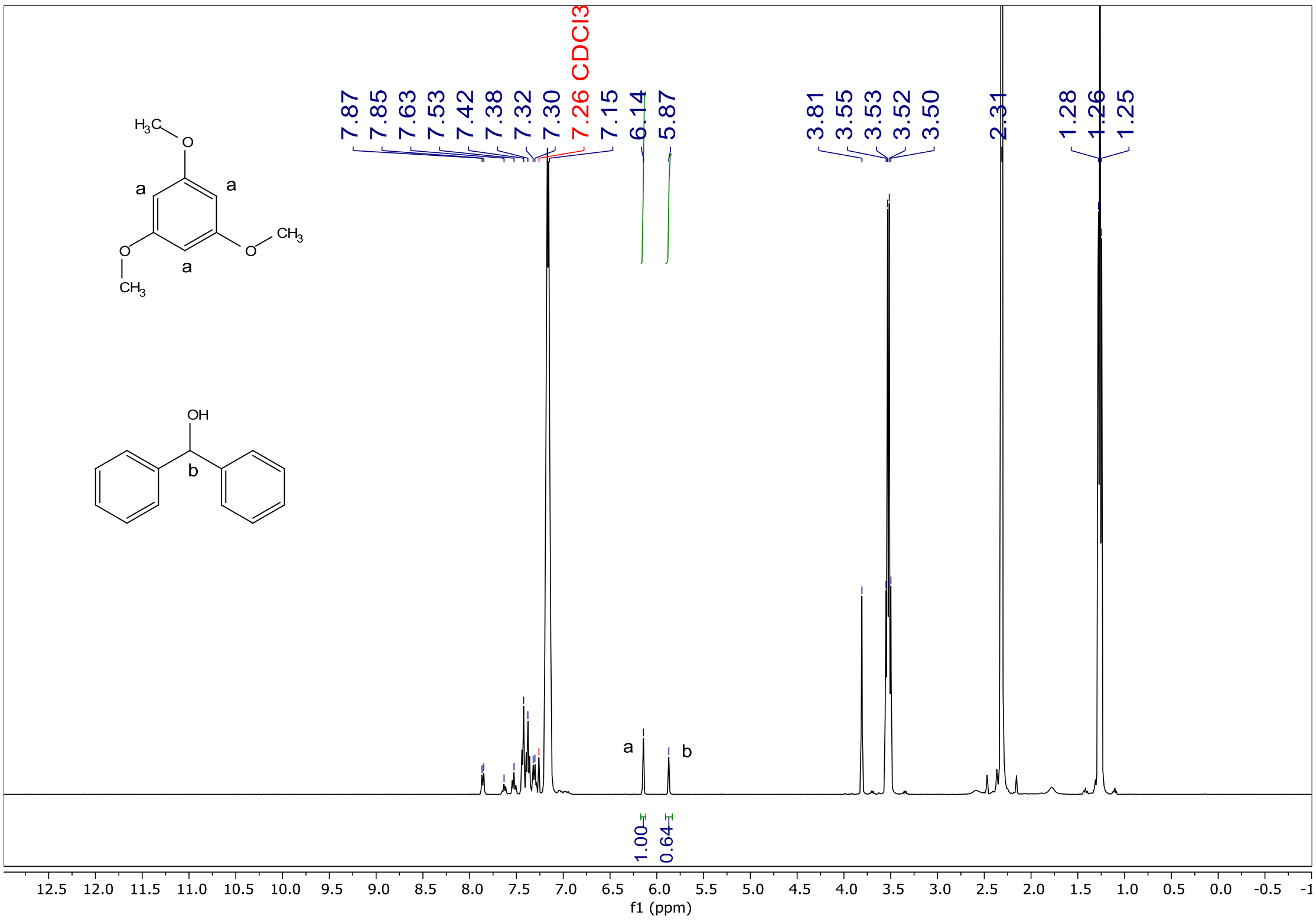

Figure S42: ^{1}H NMR spectrum ($CDCl_3$, 400 MHz, 298 K) for Table 3, entry 1.

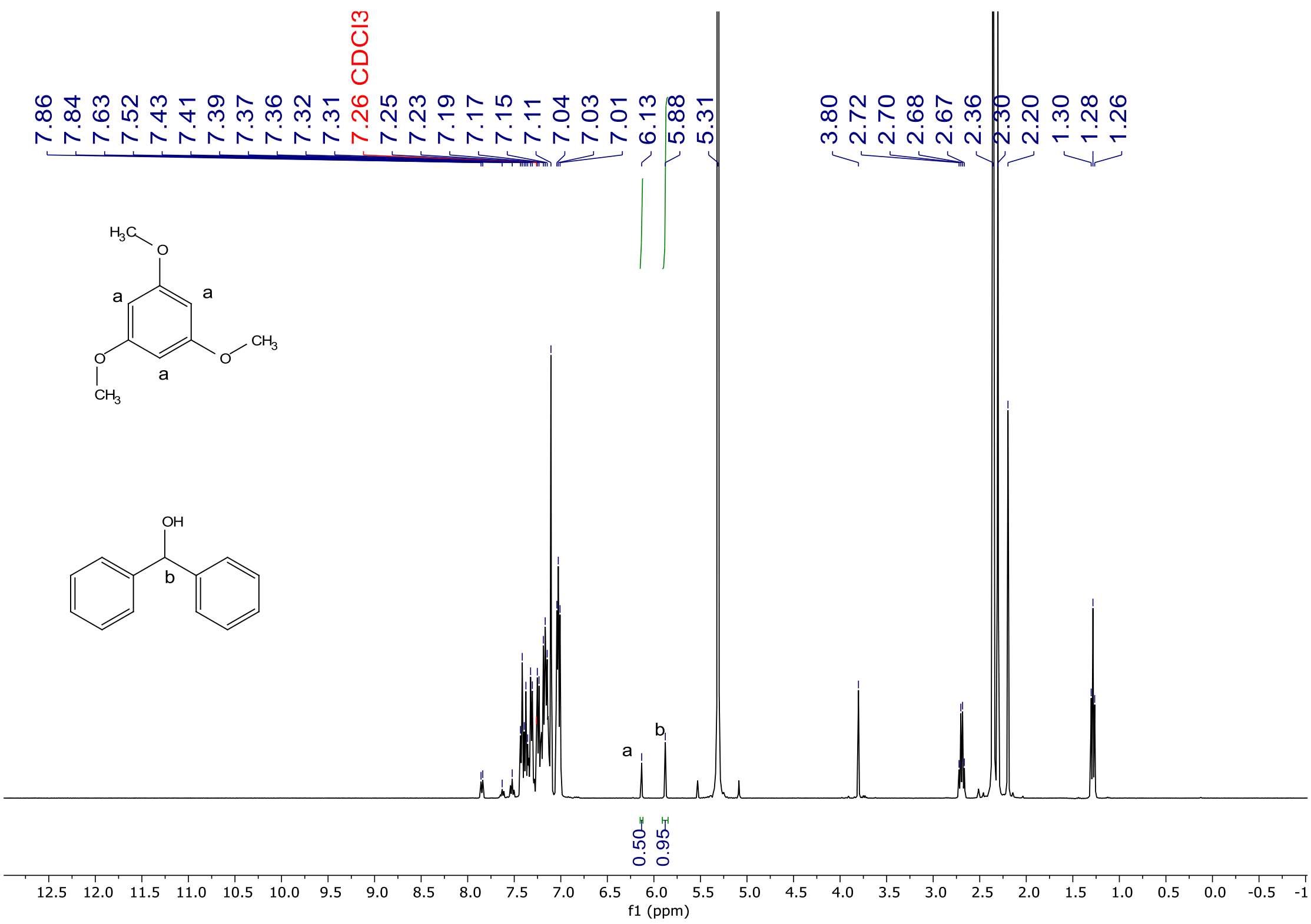

Figure S43: ^{1}H NMR spectrum ($CDCl_3$, 400 MHz, 298 K) for Table 3, entry 2.

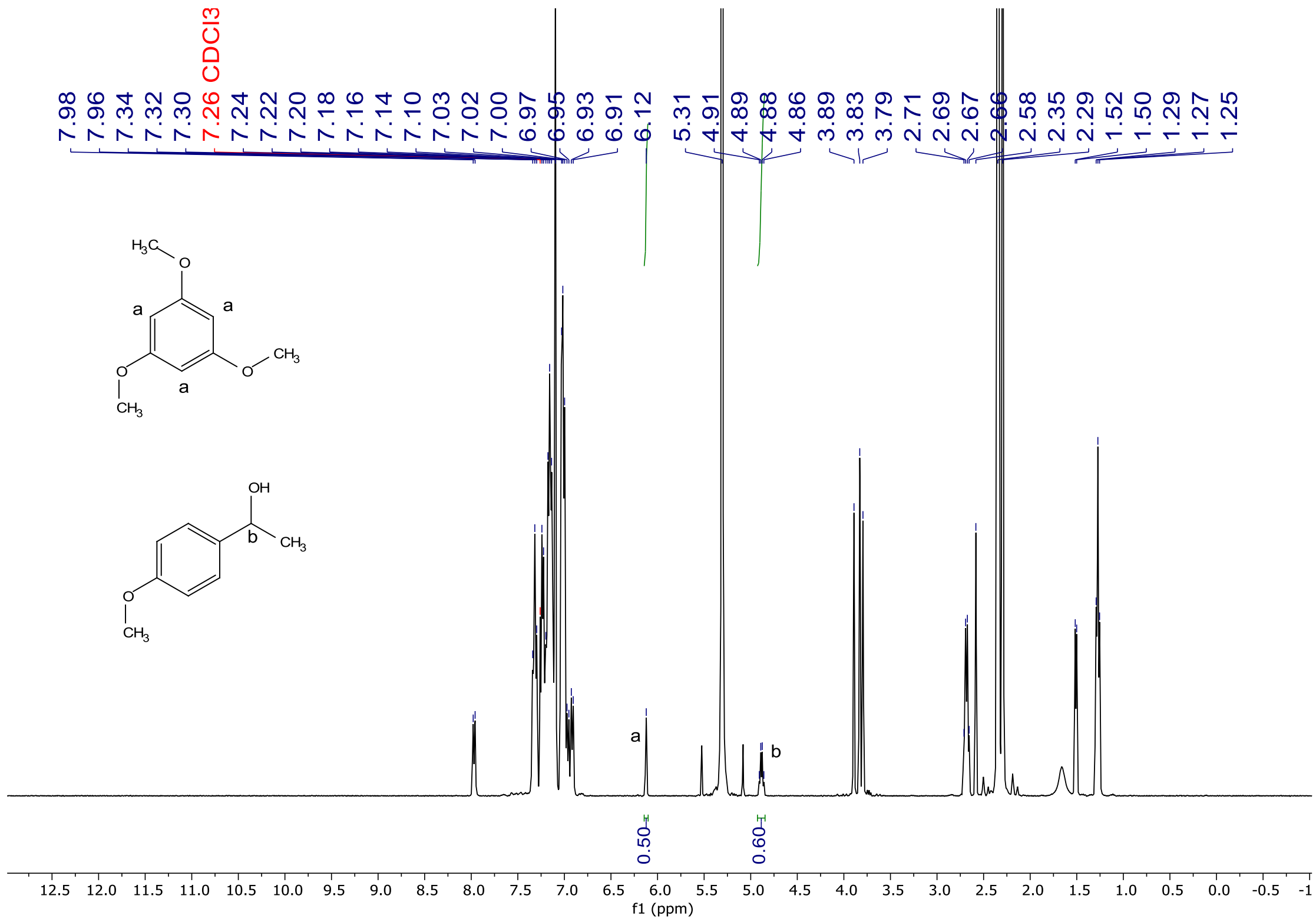

Figure S44: ^{1}H NMR spectrum ($CDCl_3$, 400 MHz, 298 K) for Table 3, entry 3.

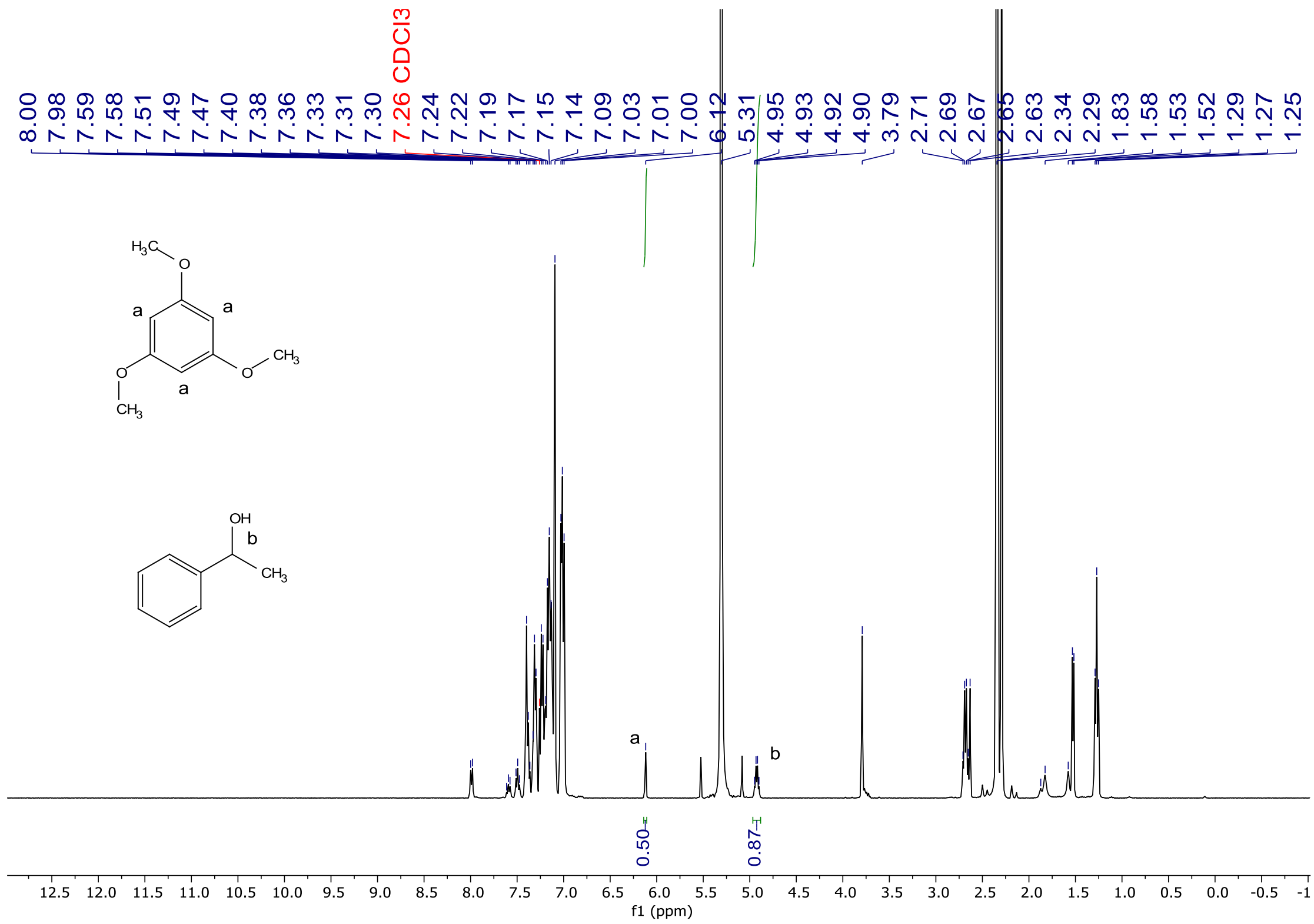

Figure S45: ^{1}H NMR spectrum ($CDCl_3$, 400 MHz, 298 K) for Table 3, entry 4.

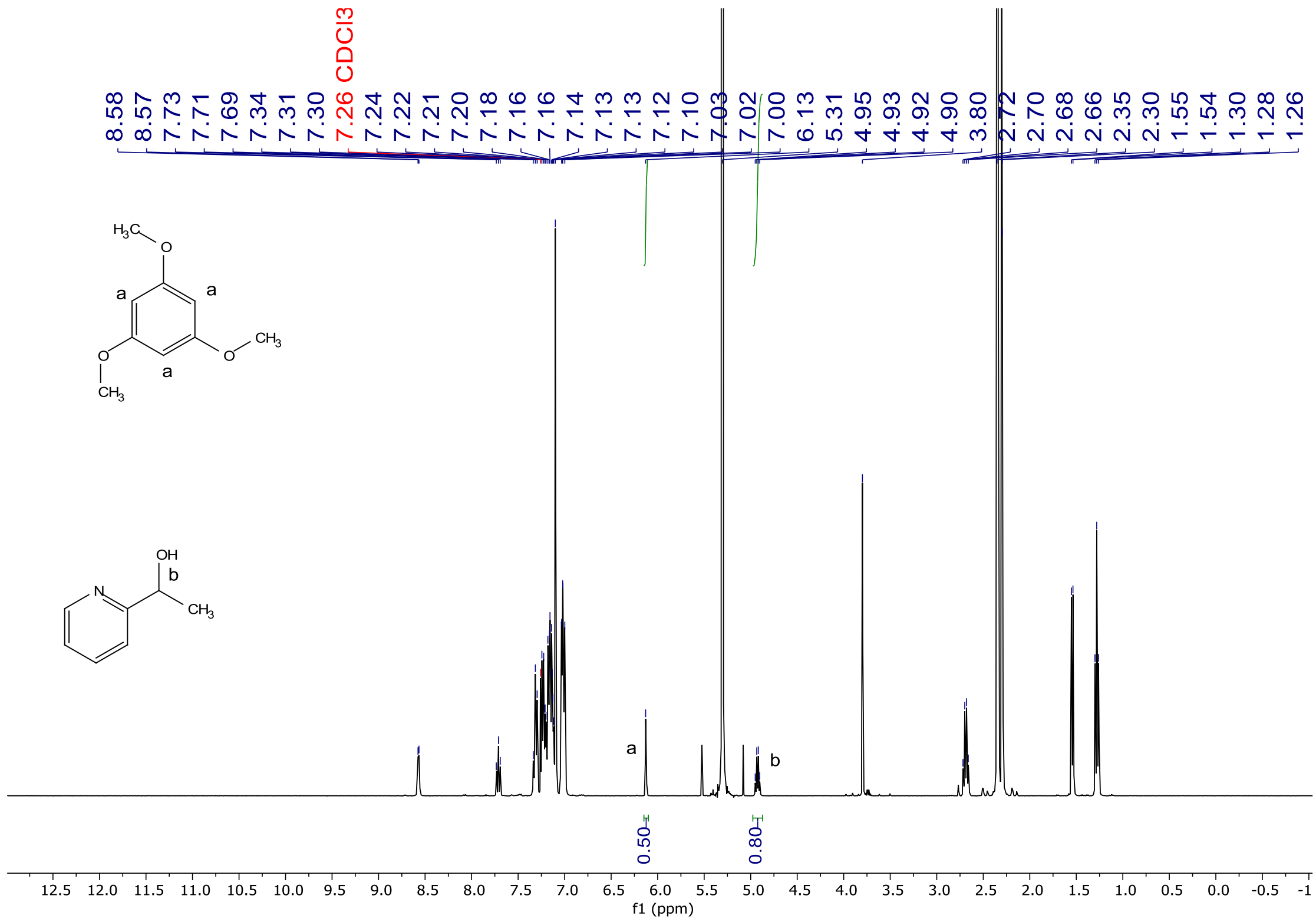

Figure S46: ^{1}H NMR spectrum ($CDCl_3$, 400 MHz, 298 K) for Table 3, entry 5.

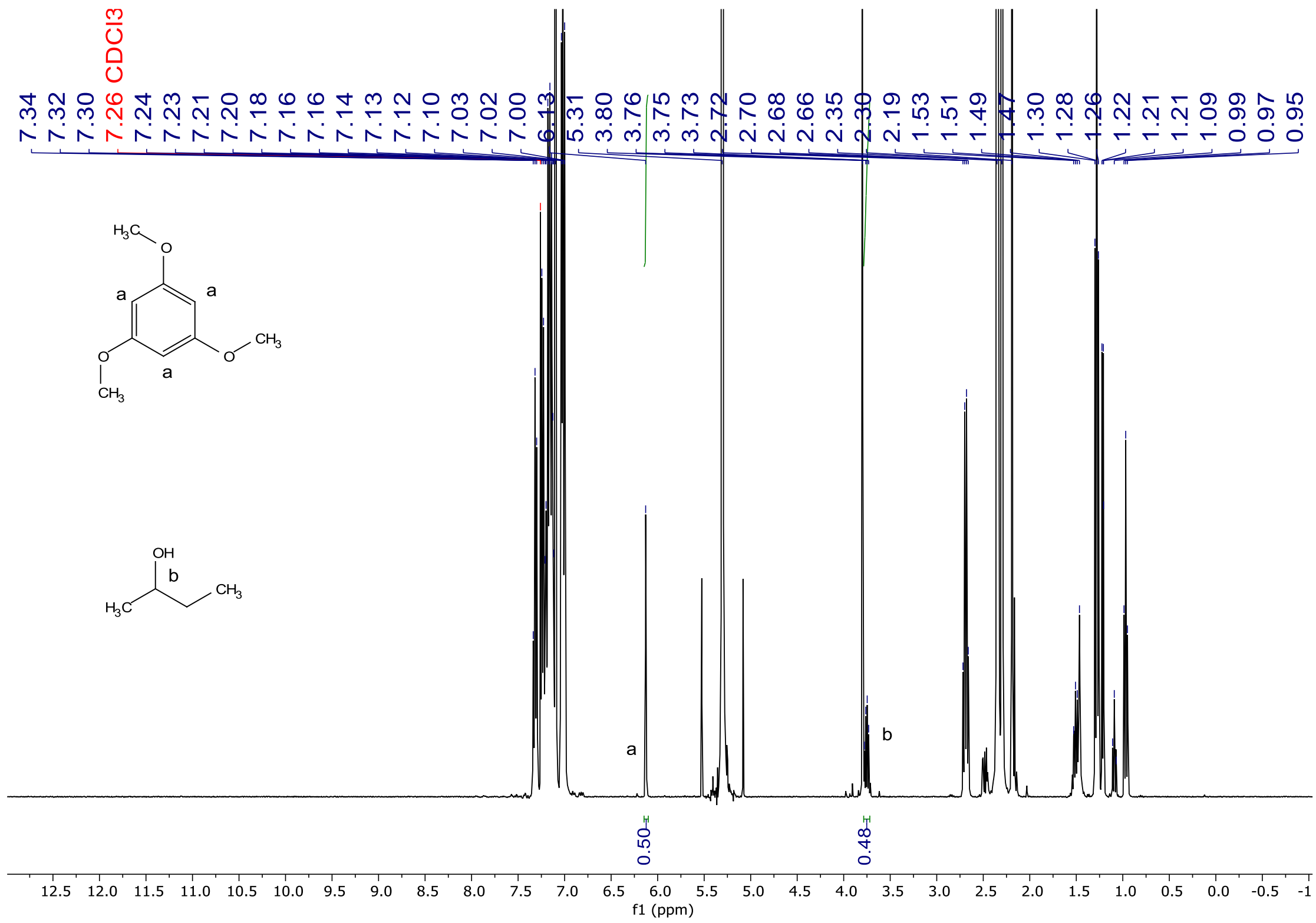

Figure S47: ^{1}H NMR spectrum ($CDCl_3$, 400 MHz, 298 K) for Table 3, entry 6.

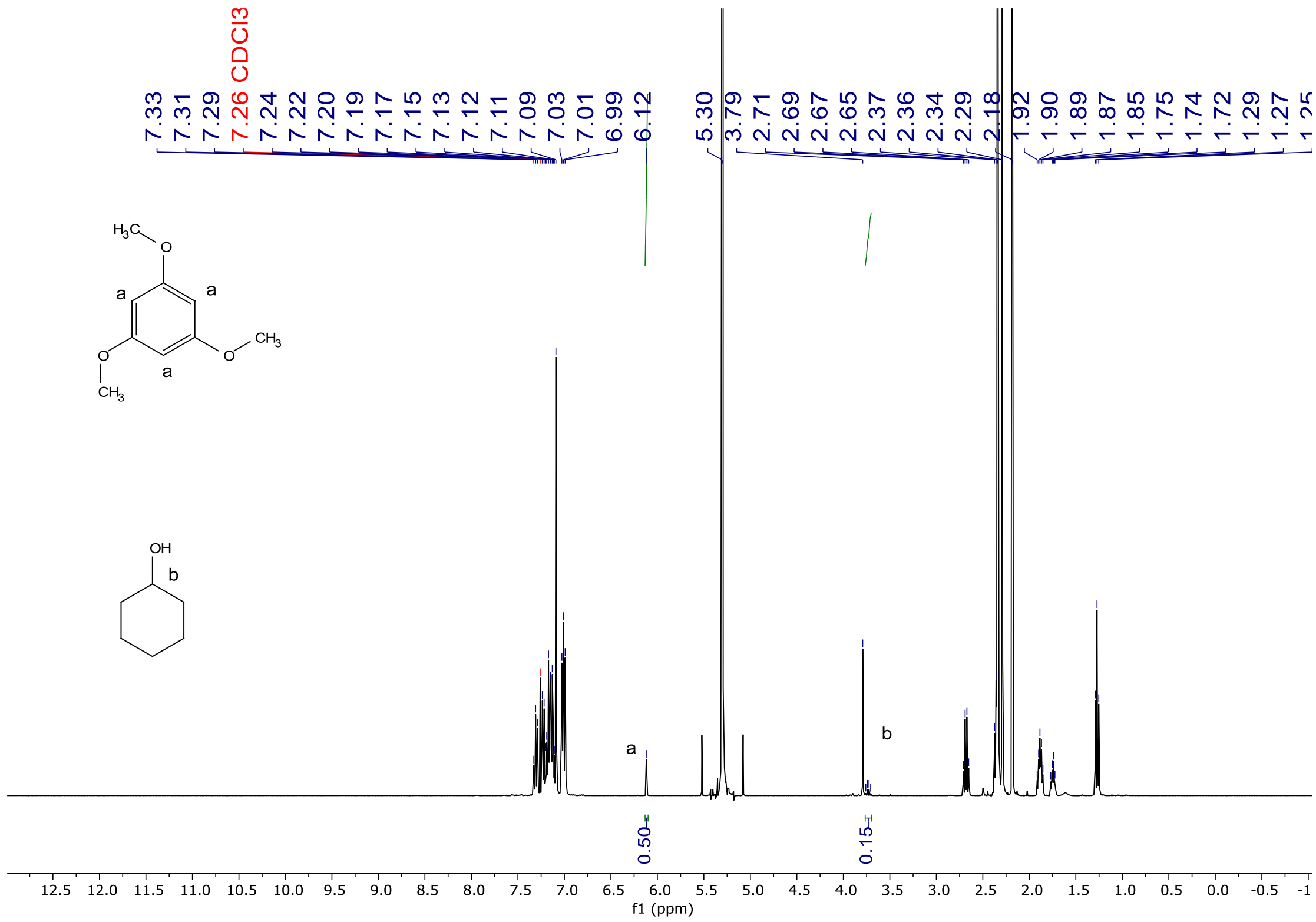

Figure S48: ^{1}H NMR spectrum ($CDCl_3$, 400 MHz, 298 K) for Table 3, entry 7.

4.4. NMR data for NMR scale reactions

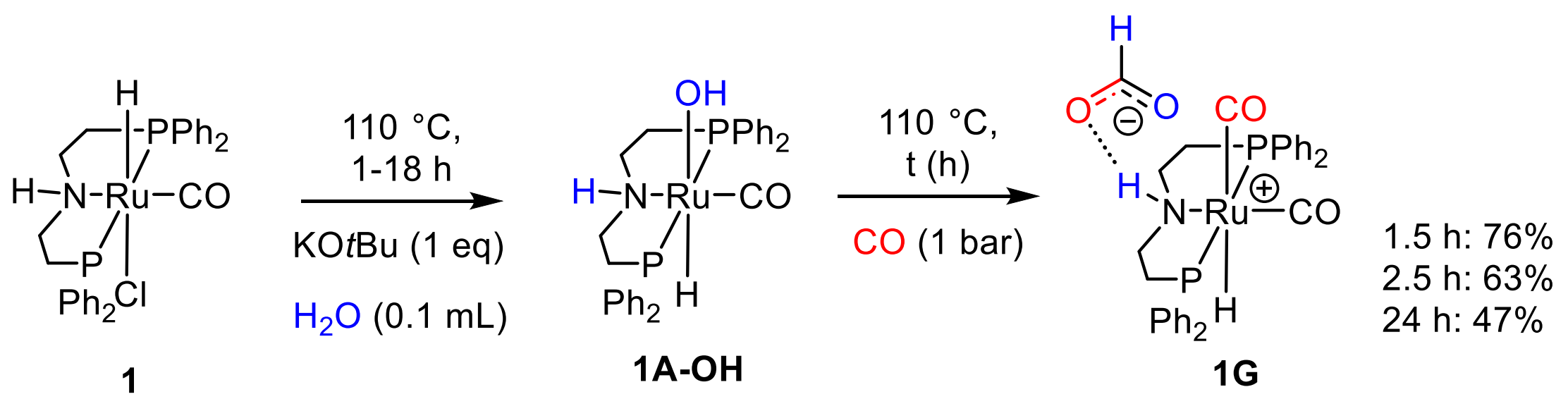

Figure S49: Behaviour of species **1** with H_2O and CO as monitored by ^{1}H and $^{31}P\{^{1}H\}$ NMR spectroscopy.

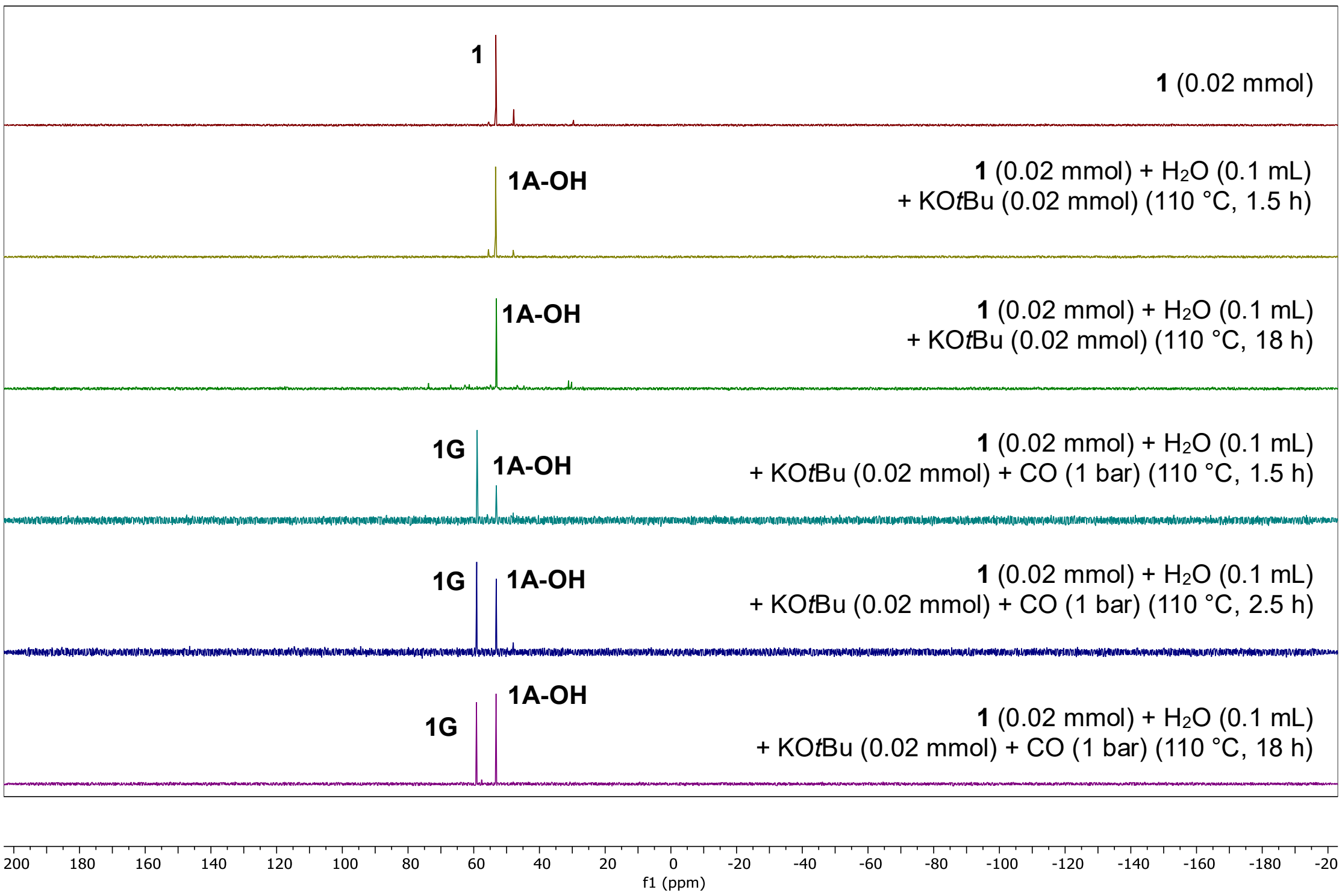

Figure S50: Overlayed $^{31}P\{^1H\}$ NMR spectra (d_8-toluene, 162 MHz, 298 K) corresponding to speciation of organometallic species observed during NMR scale experiments.

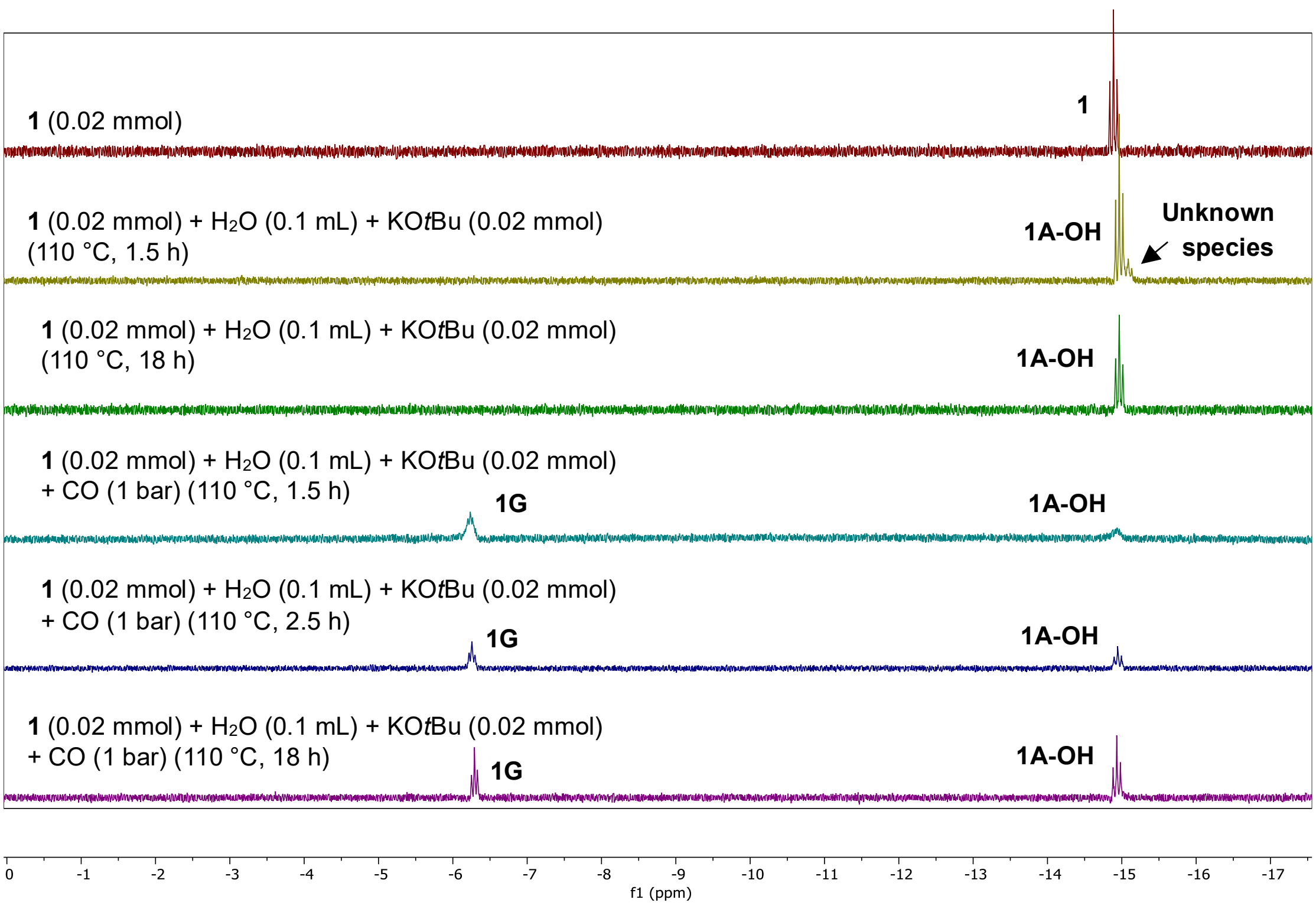

Figure S51: Overlayed hydride region of ^1H NMR spectra (d_8-toluene, 400 MHz, 298 K) corresponding to speciation of organometallic species observed during NMR scale experiments.

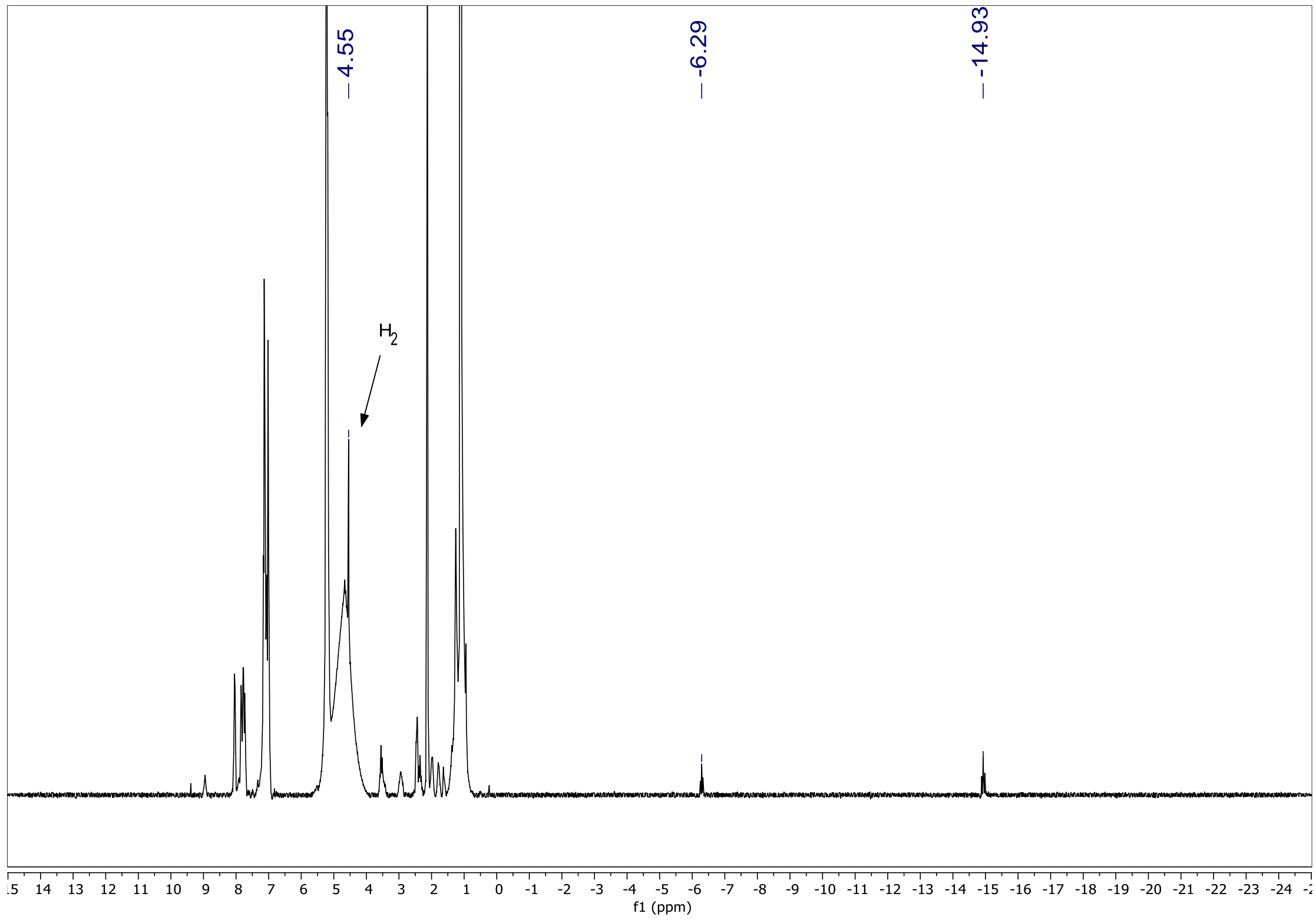

Figure S52: ^{1}H NMR spectrum (d_8-toluene, 400 MHz, 298 K) of **1** (0.02 mmol), KO*t*Bu (0.04 mmol), H_2O (0.1 mmol), CO (1 bar), 110 °C, 2.5 h.

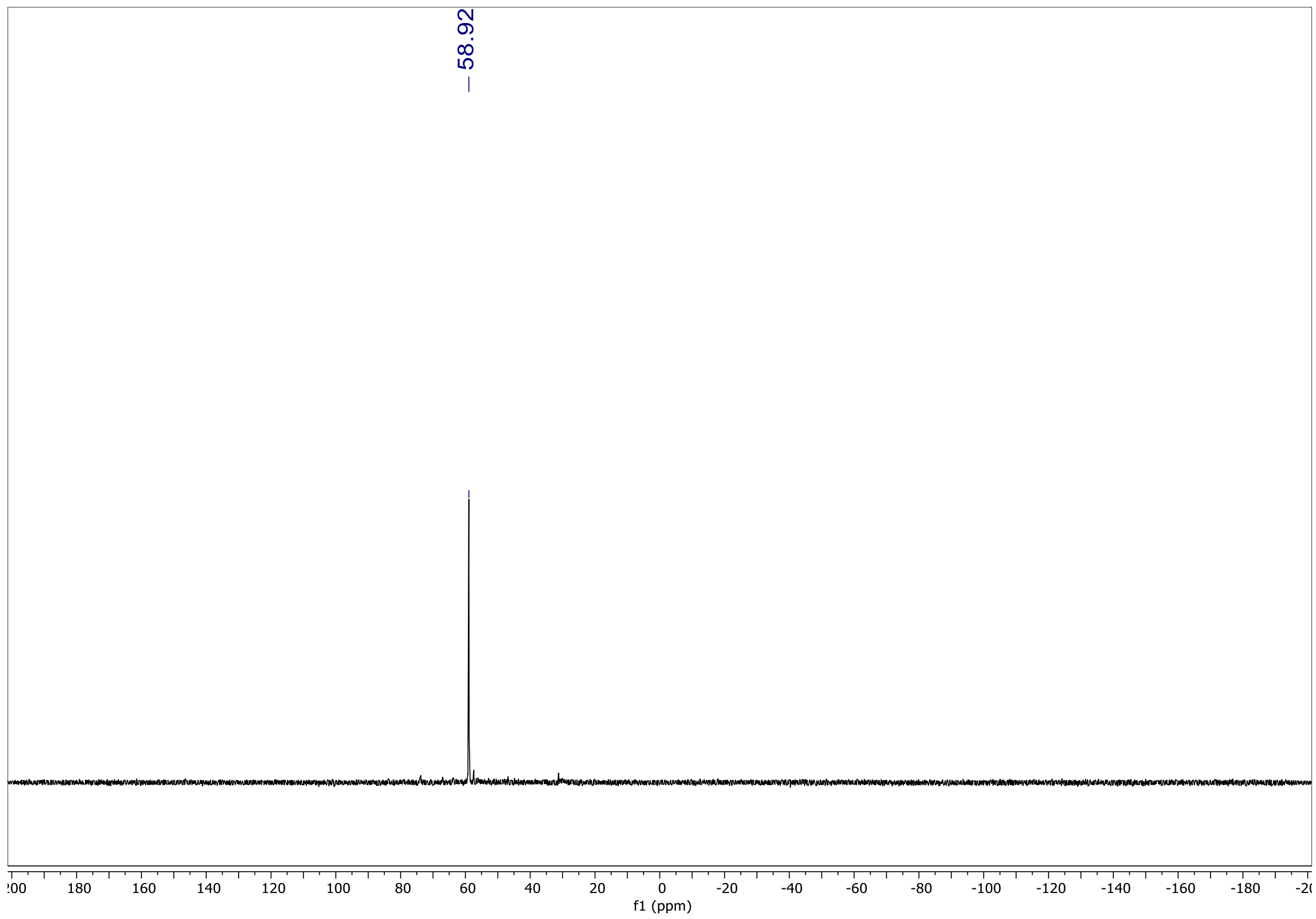

Figure S53: $^{31}P\{^{1}H\}$ NMR spectrum (d_8-toluene, 400 MHz, 298 K) of **1** (0.02 mmol), KO*t*Bu (0.04 mmol), H_2O (0.1 mmol), CO (1 bar), 110 °C, 0.5 h.

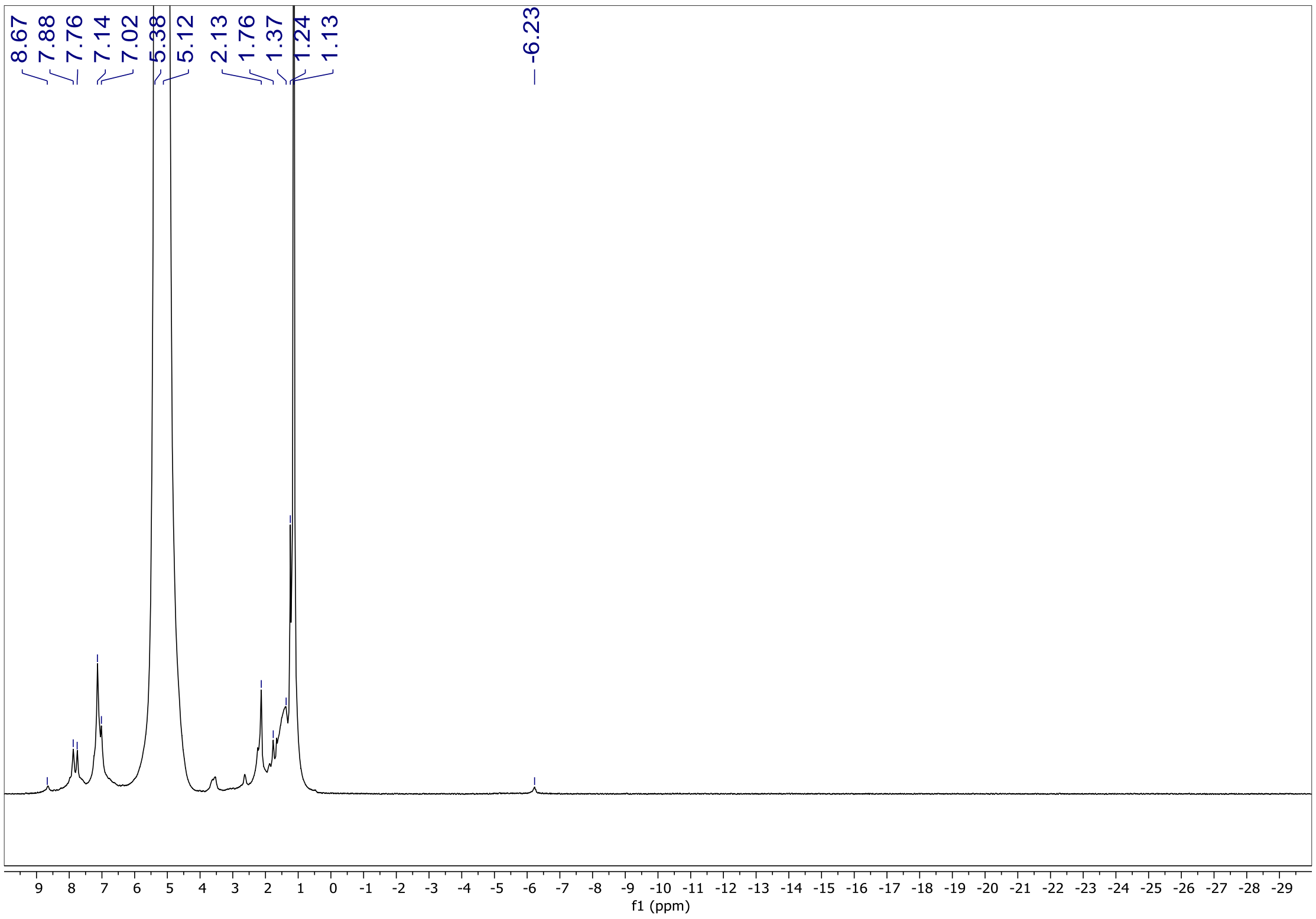

Figure S54: ^{1}H NMR spectrum (d_8-toluene, 162 MHz, 298 K) of **1** (0.02 mmol), KO*t*Bu (0.04 mmol), H_2O (0.1 mmol), CO (1 bar), 110 °C, 0.5 h.

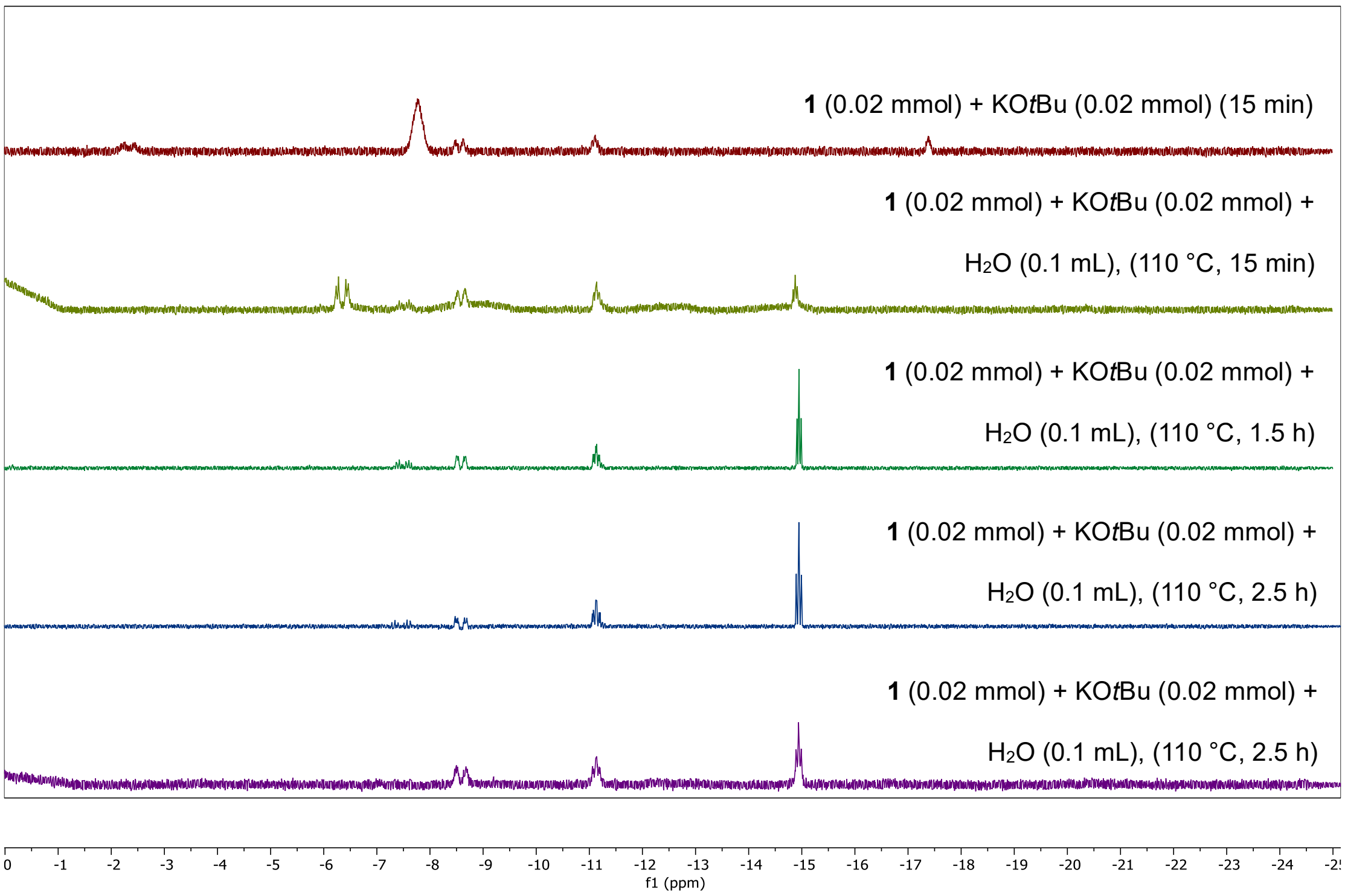

Figure S55: Overlayed hydride region of ^{1}H NMR spectra (d_8-toluene, 400 MHz, 298 K) corresponding to speciation of organometallic species observed during sequential addition of KO*t*Bu then H_2O to **1**.

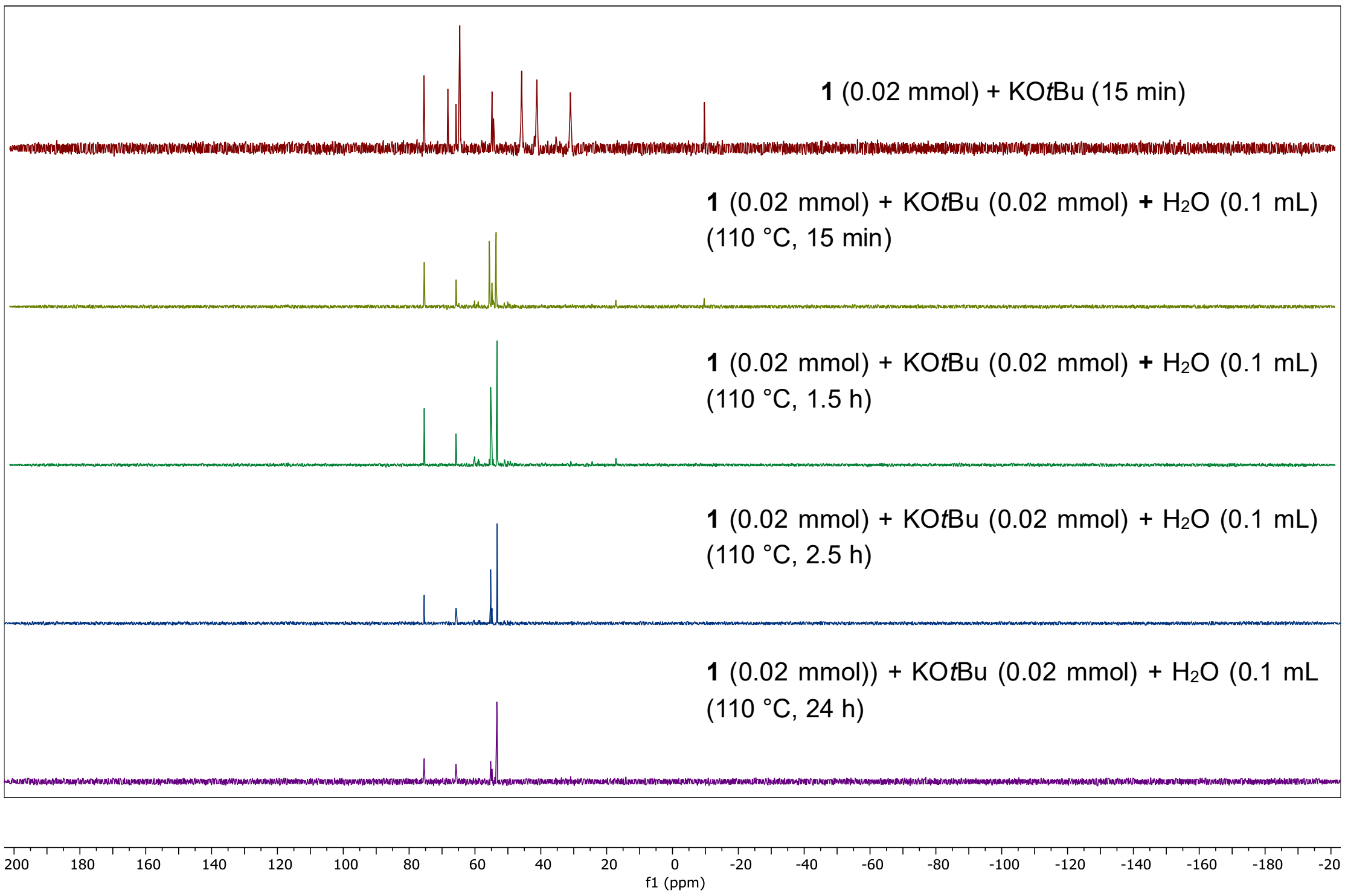

Figure S56: Overlayed $^{31}P\{^{1}H\}$ NMR spectra (d_8-toluene, 162 MHz, 298 K) corresponding to speciation of organometallic species observed during sequential addition of KO*t*Bu then H_2O to **1**.

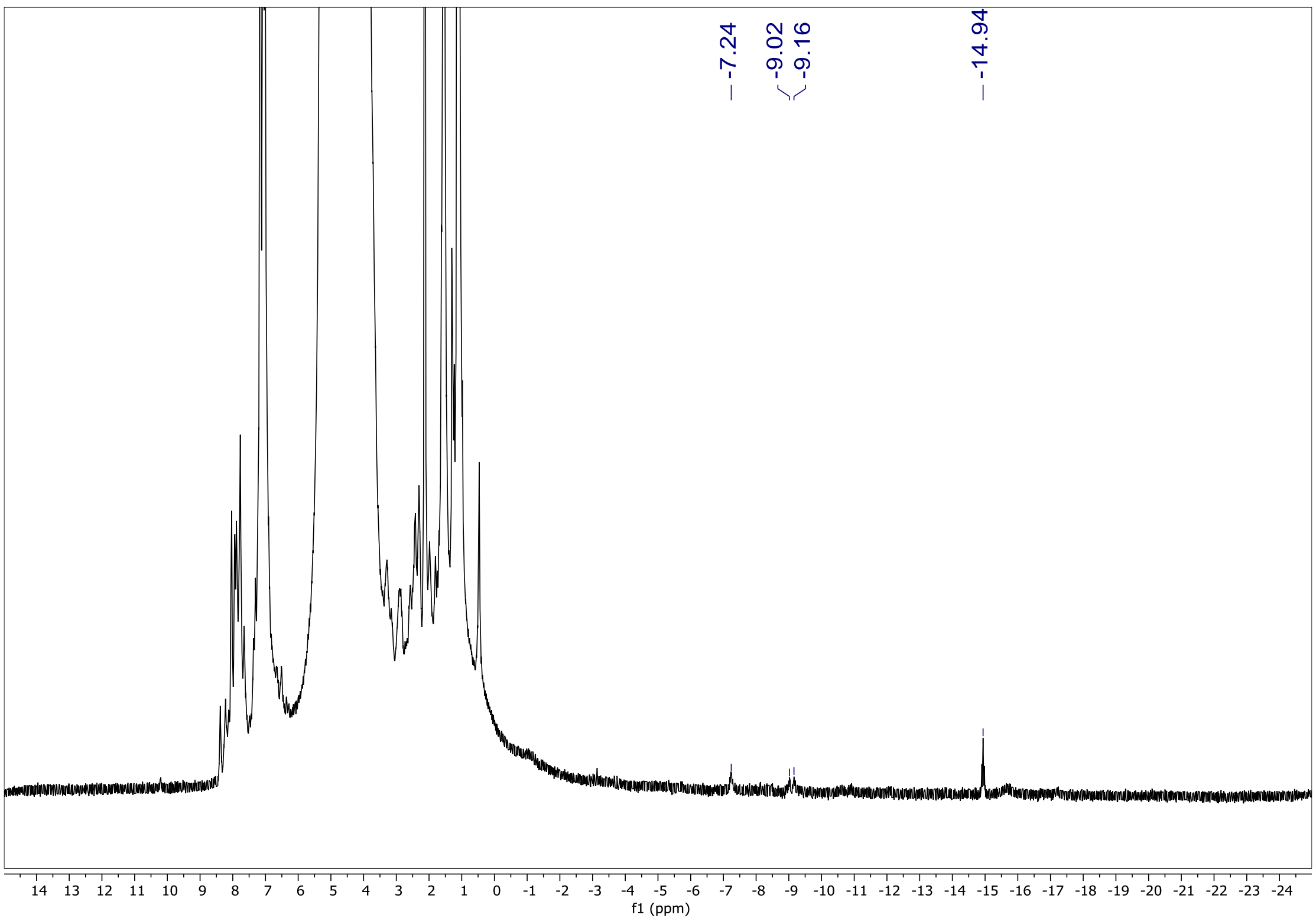

Figure S57: ^{1}H NMR spectrum (d_8-toluene, 400 MHz, 298 K) of **5** (0.02 mmol), H_2O (0.1 mmol), 110 °C, 2 h.

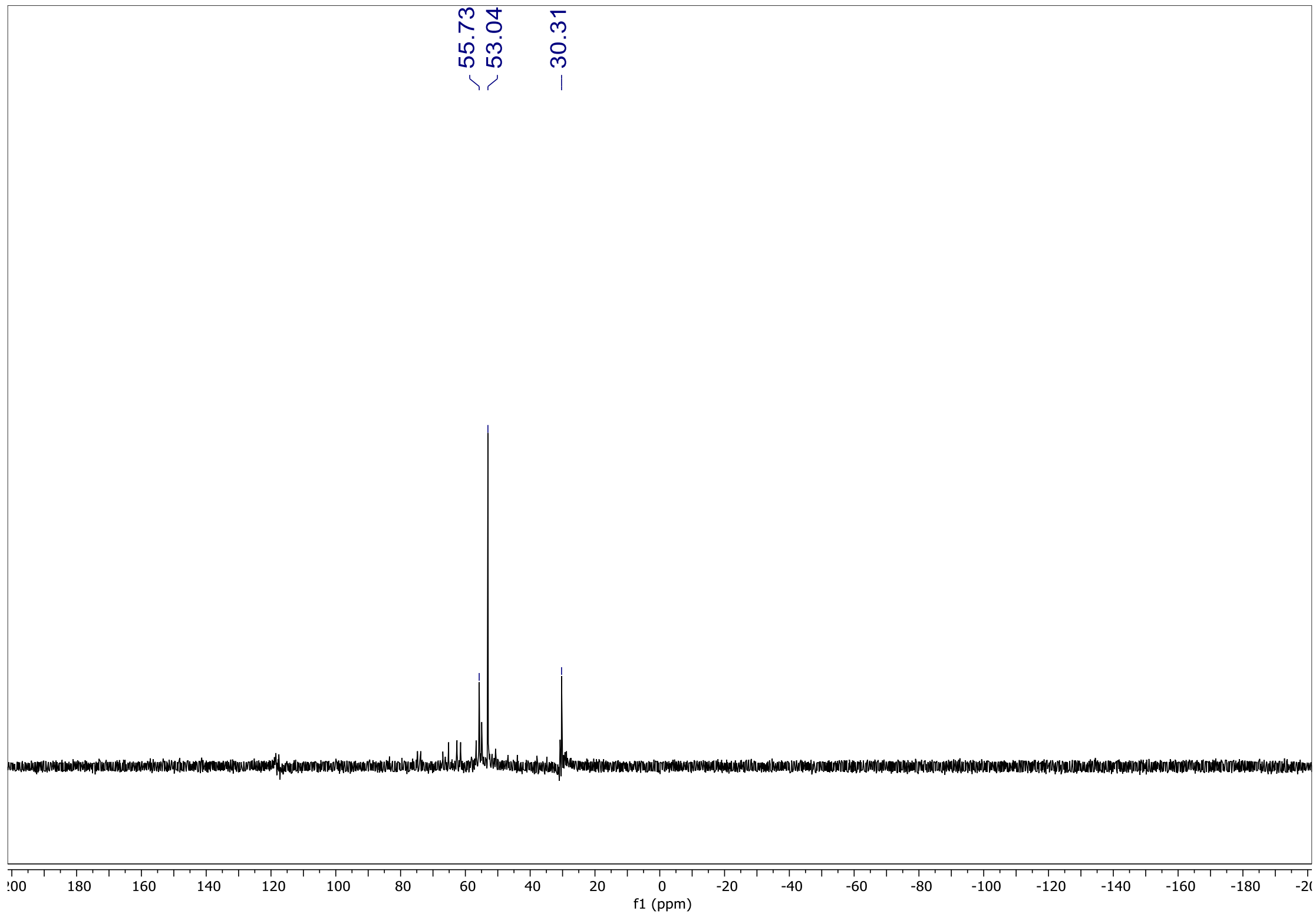

Figure S58: $^{31}P\{^{1}H\}$ NMR spectrum (d_8-toluene, 162 MHz, 298 K) of **5** (0.02 mmol), H_2O (0.1 mmol), 110 °C, 2 h.

5. GC-MS data

5.1. GC-MS data for transfer hydrogenation reactions

For GC-MS data corresponding to Table 3, entries 6 and 7, the substrates and products are not observed due to overlapping retention time with the reaction solvent.

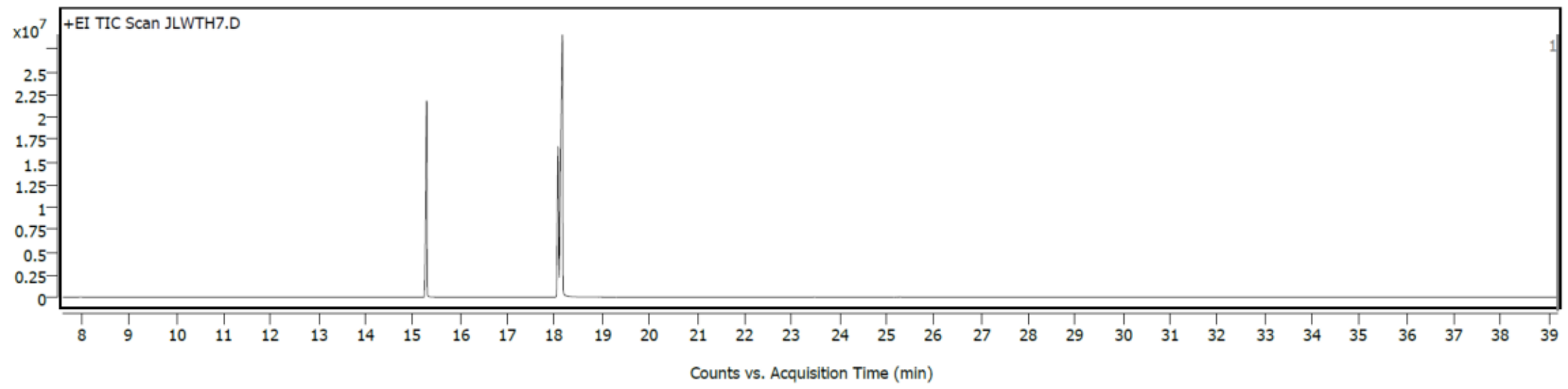

Figure S59: Gas chromatograph for Table 3, entry 1.

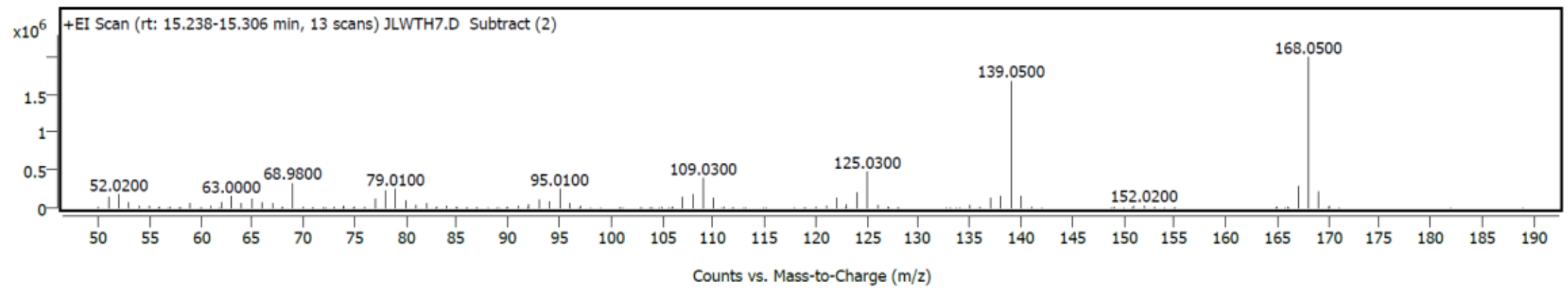

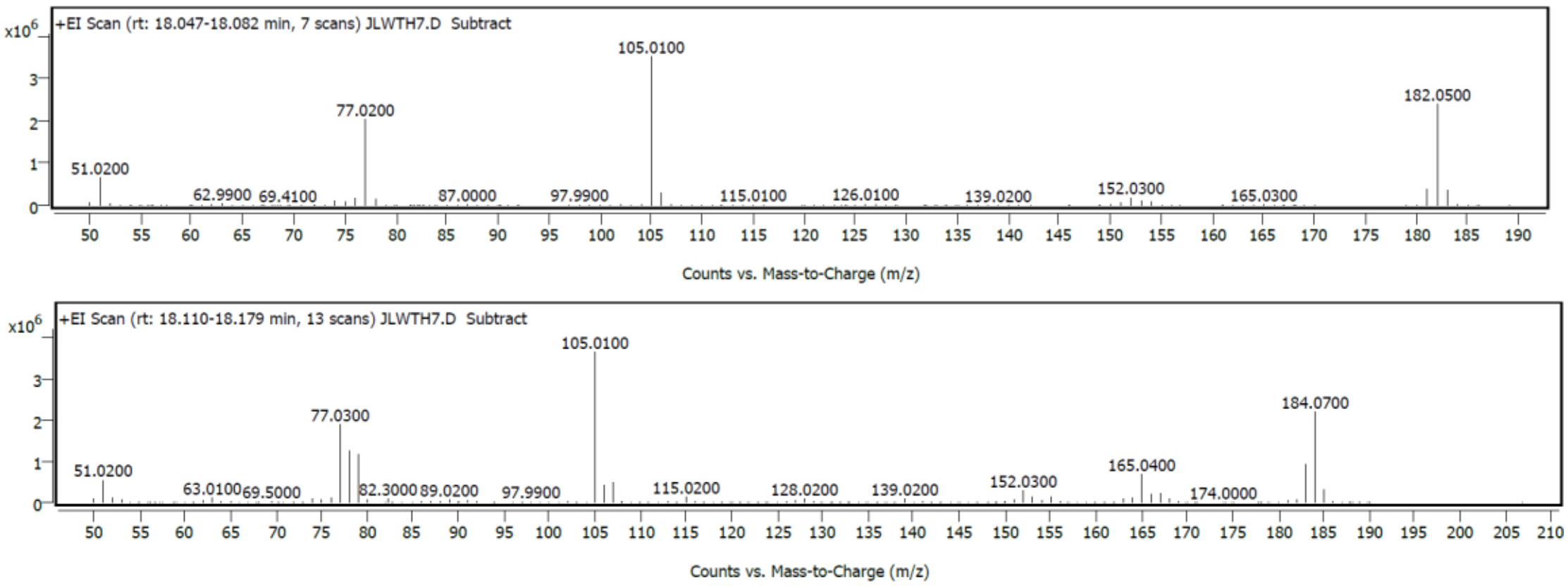

Figure S60: Mass spectra corresponding to Figure S59 (Table 3, entry 1).

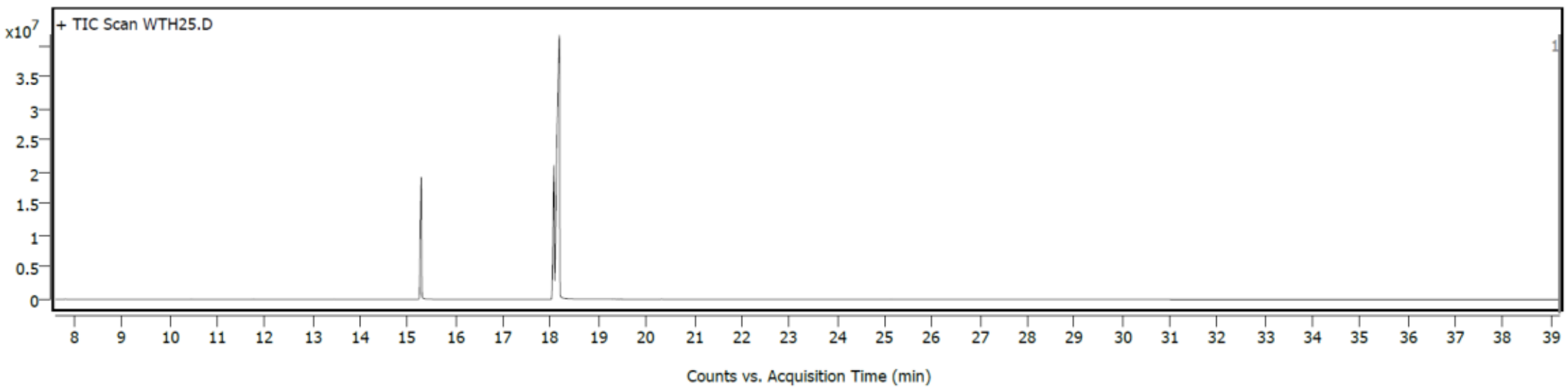

Figure S61: Gas chromatograph for Table 3, entry 2.

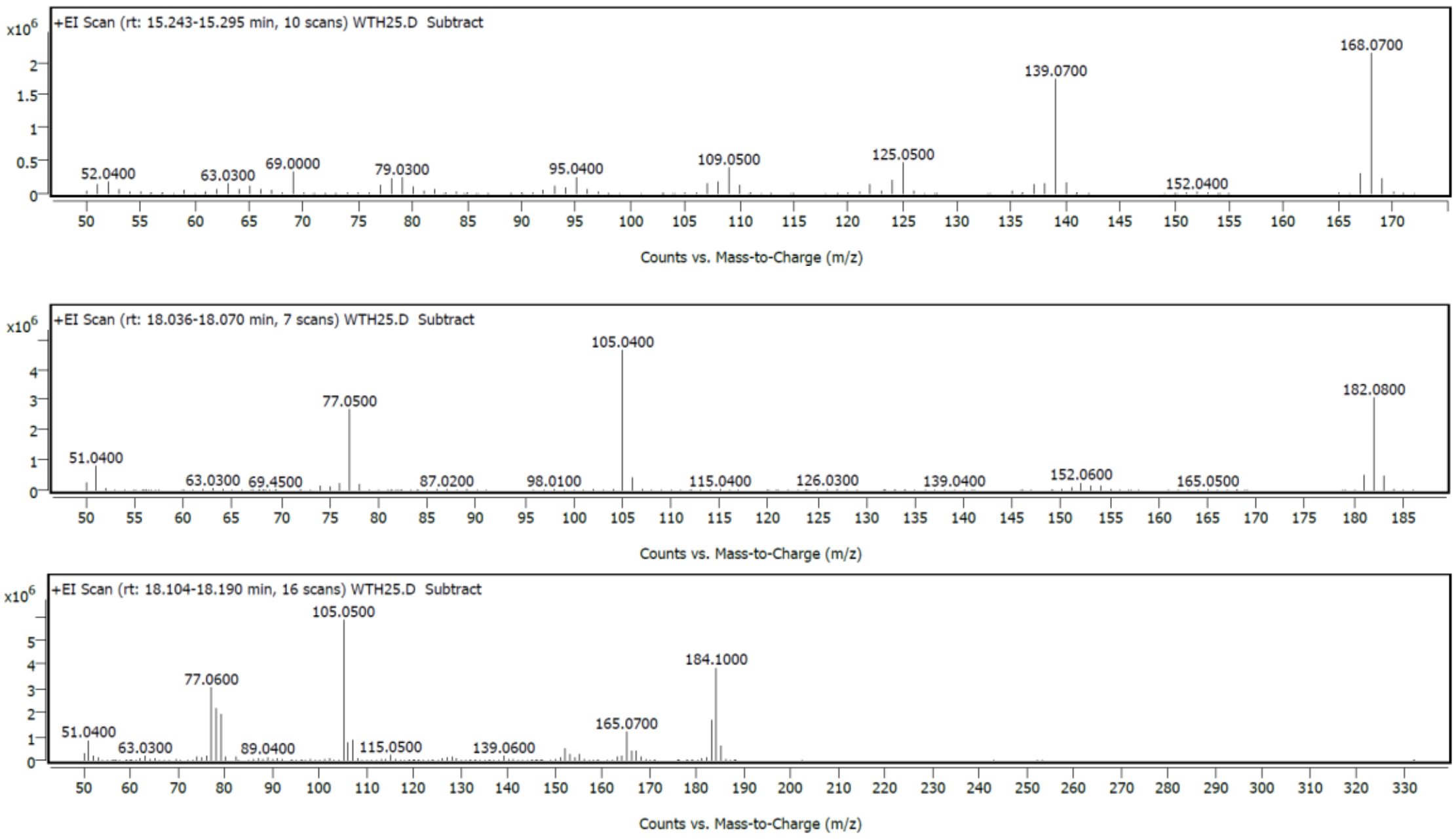

Figure S62: Mass spectra corresponding to Figure S61 (Table 3, entry 2).

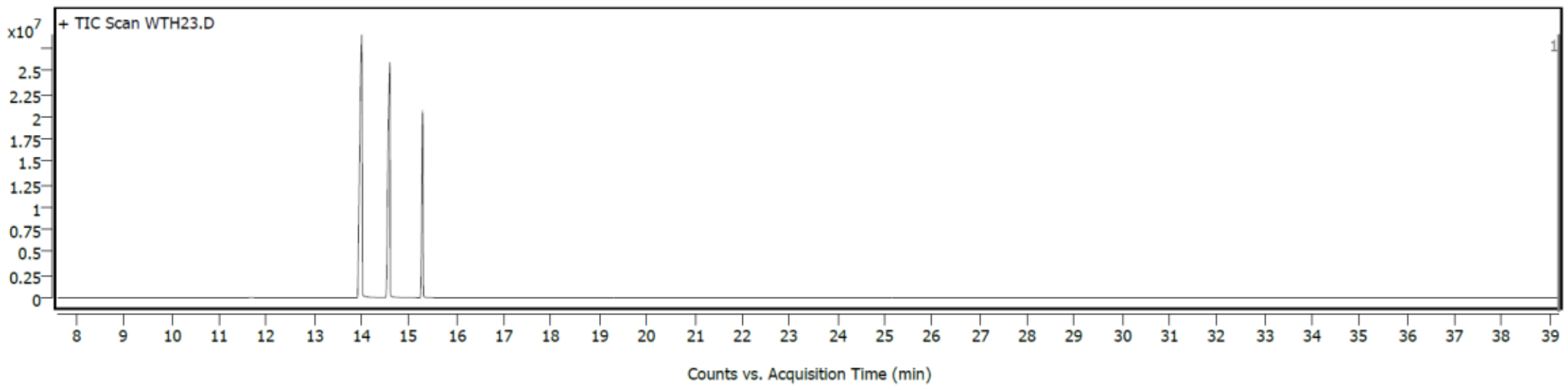

Figure S63: Gas chromatograph for Table 3, entry 3.

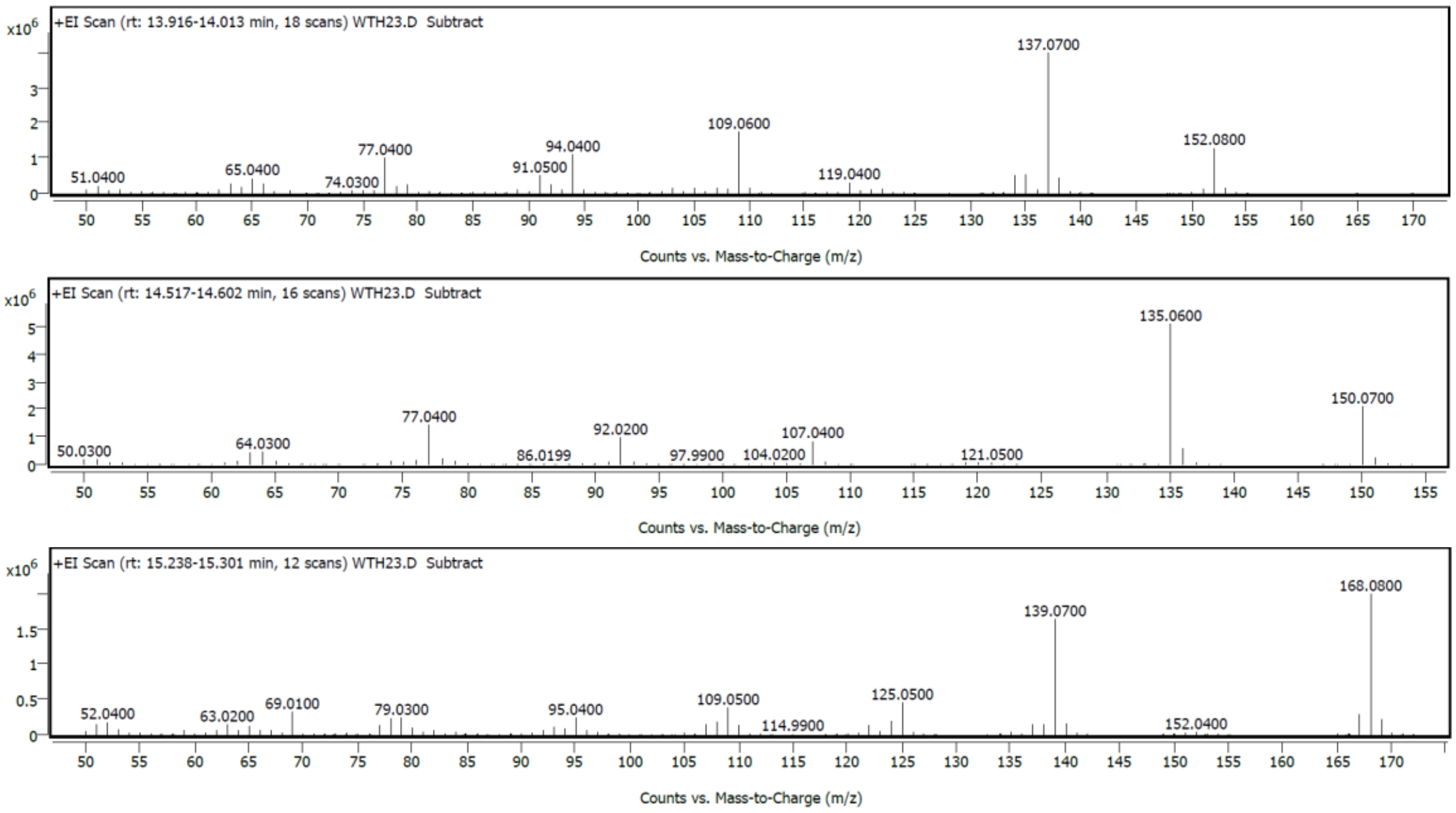

Figure S64: Mass spectra corresponding to Figure S63 (Table 3, entry 3).

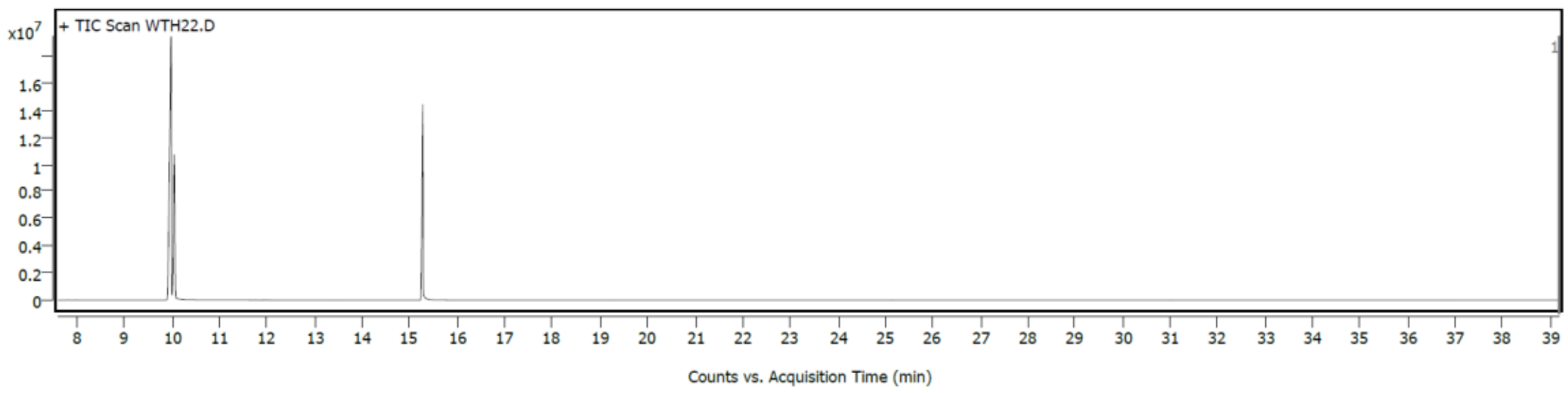

Figure S65: Gas chromatograph for Table 3, entry 4.

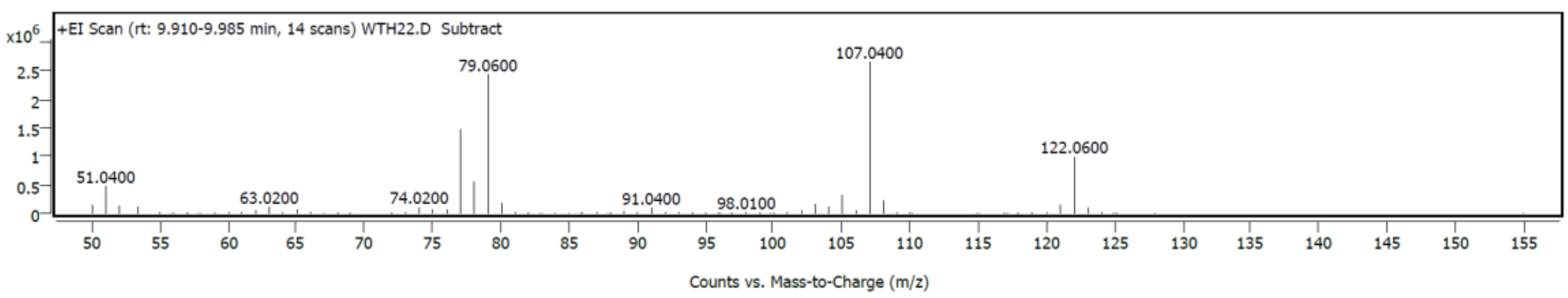

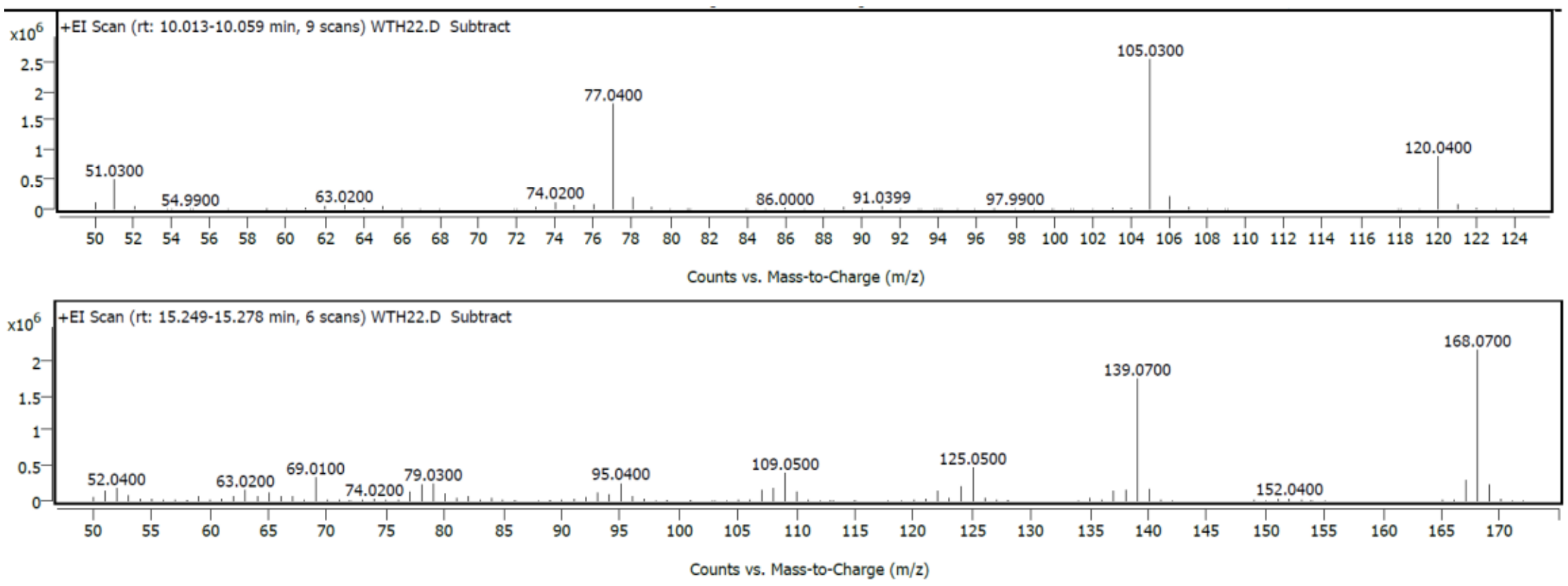

Figure S66: Mass spectra corresponding to Figure S65 (Table 3, entry 4).

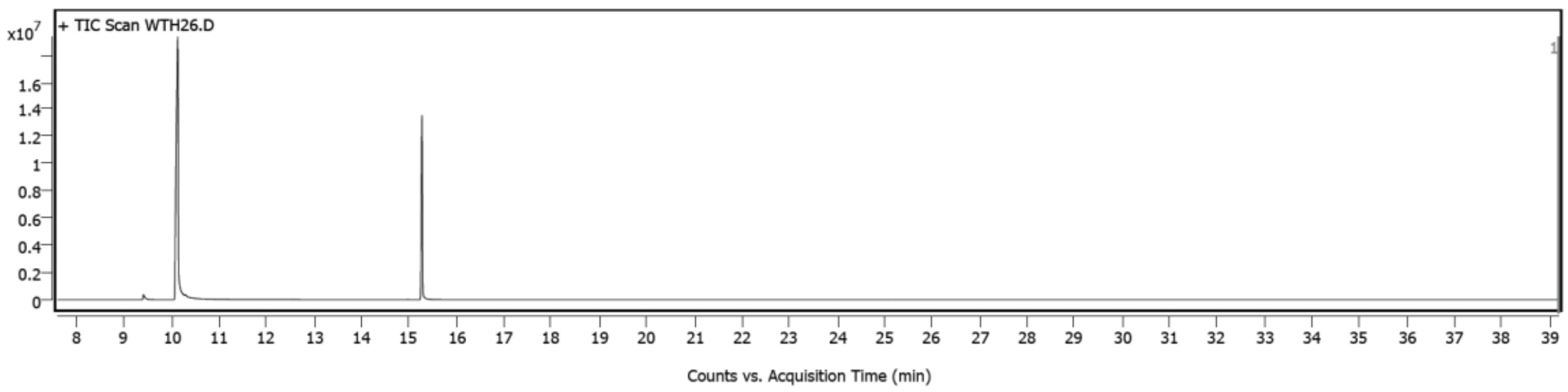

Figure S67: Gas chromatograph for Table 3, entry 5.

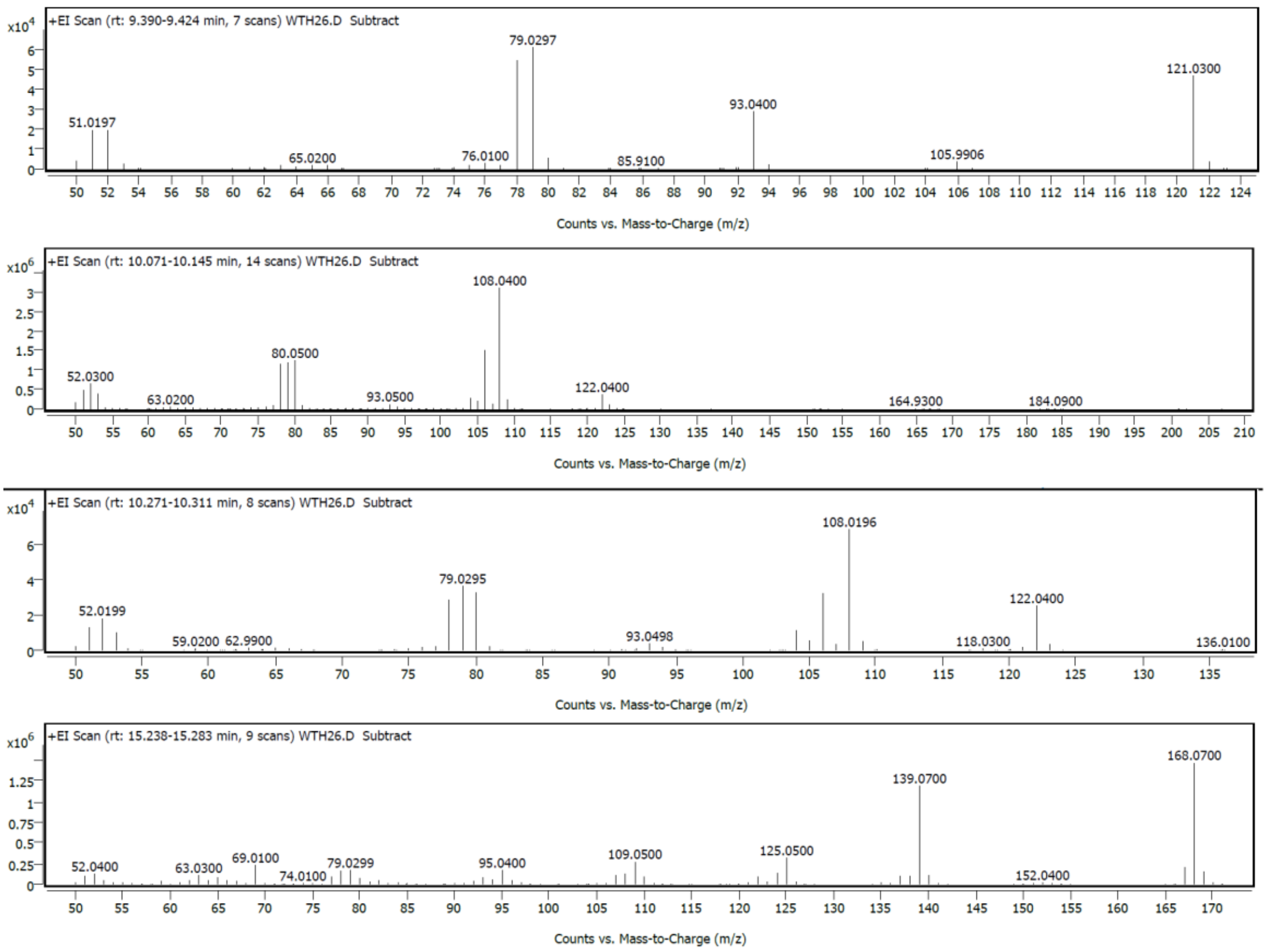

Figure S68: Mass spectra corresponding to Figure S67 (Table 3, entry 5).

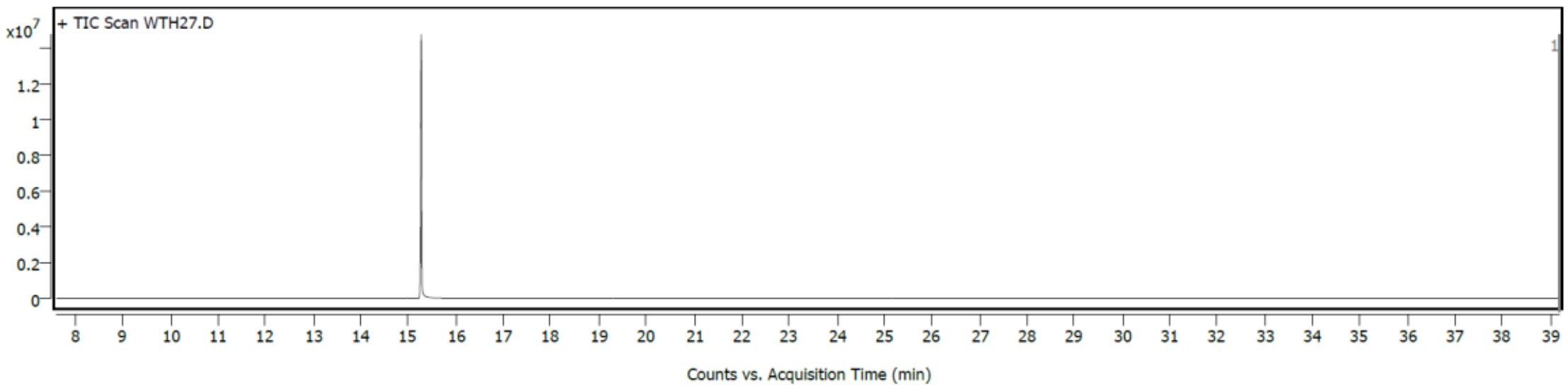

Figure S69: Gas chromatograph for Table 3, entry 6.

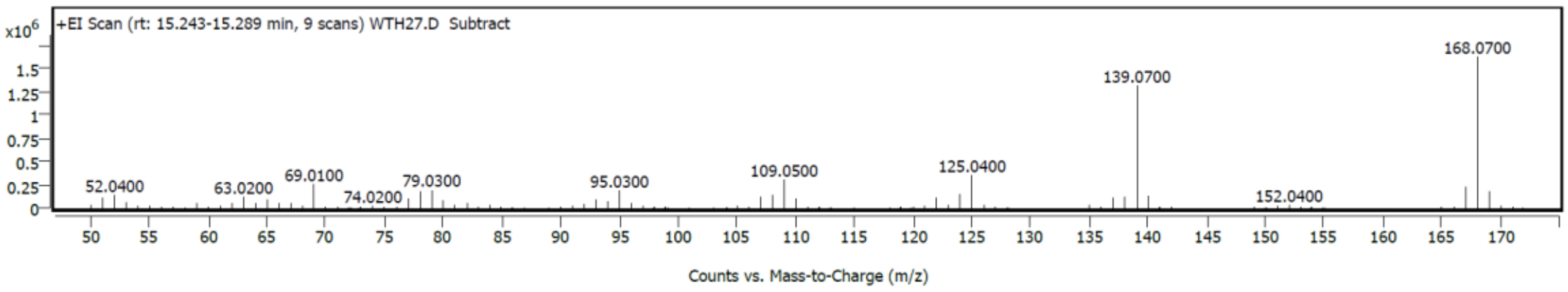

Figure S70: Mass spectrum corresponding to Figure S69 (Table 3, entry 6).

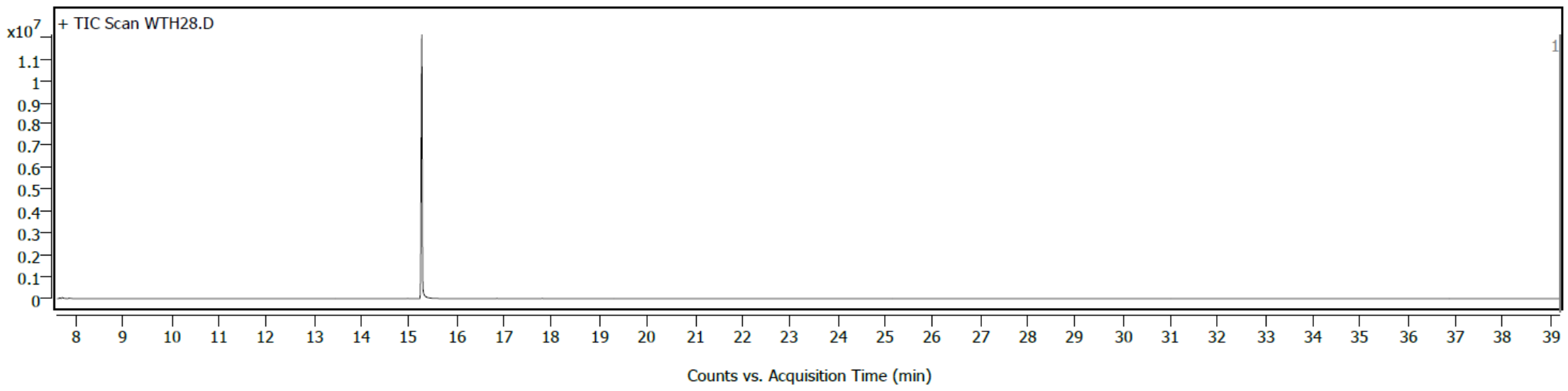

Figure S71: Gas chromatograph for Table 3, entry 7.

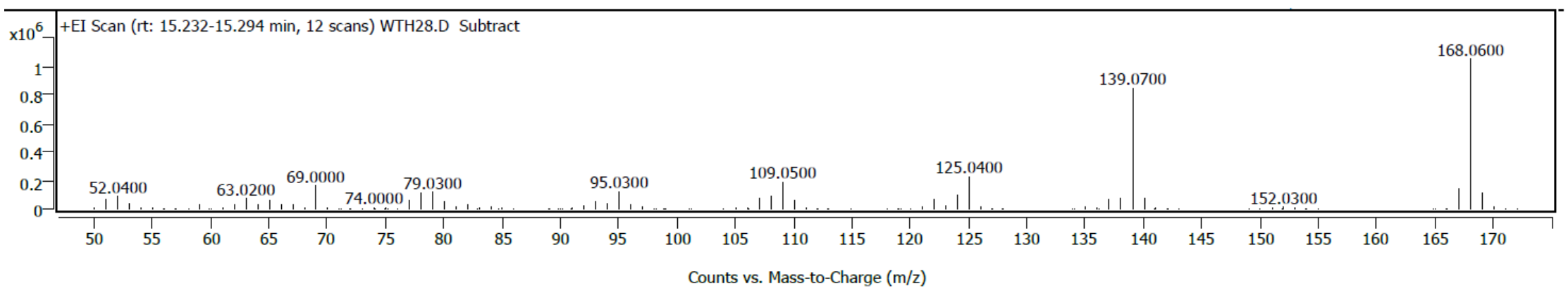

Figure S72: Mass spectrum corresponding to Figure S71 (Table 3, entry 7).

6. Crystallography data

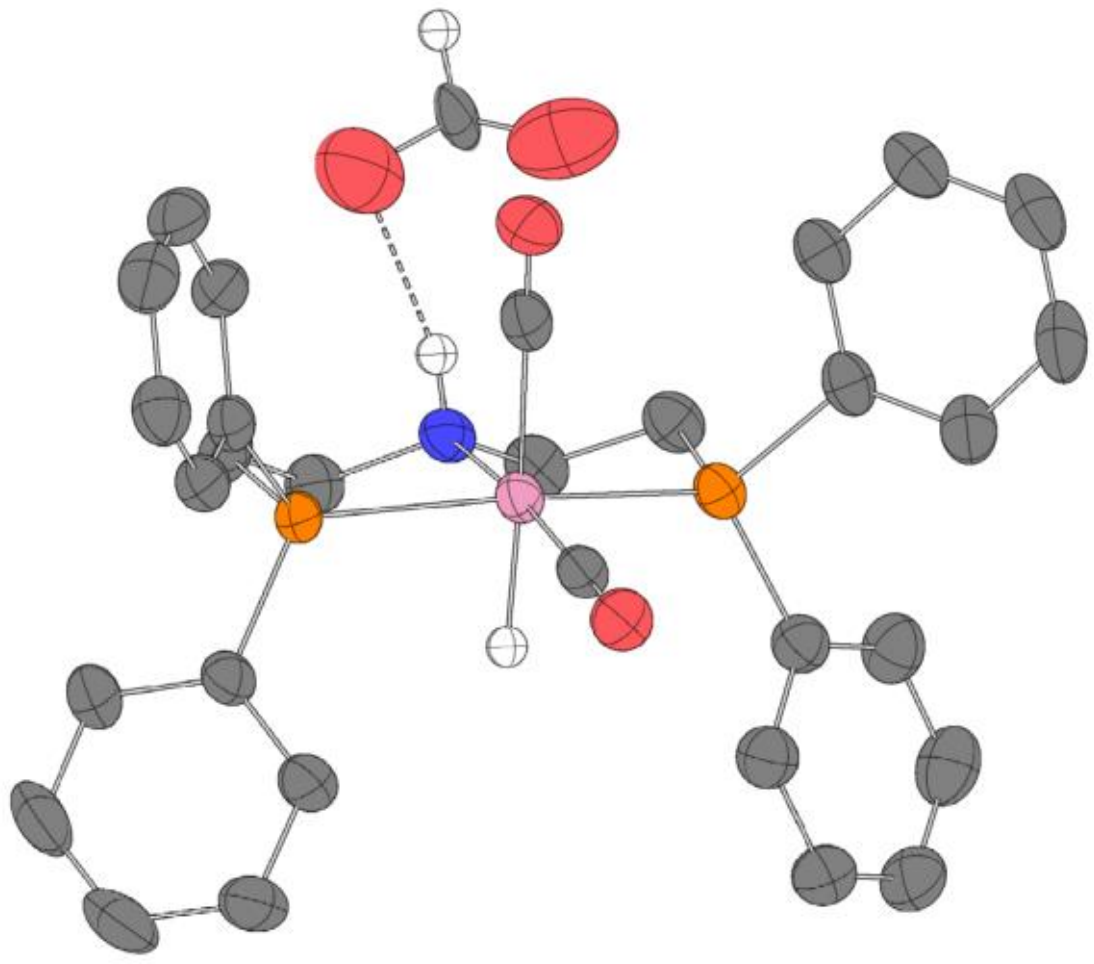

Figure S73: Single-crystal X-ray structures of the asymmetric unit cell of complex 1G. ORTEP diagrams plotted at 50% probability level. Selected hydrogen atoms have been omitted for clarity.

Table S1: Crystallographic data for complex **1G**.

**Compound**	**1G**
Chemical formula	$C_{31}H_{31}NO_4P_2Ru$
CCDC deposition number	2553626
Formula weight	644.58
Temperature (K)	100.00
Crystal System	Triclinic
Space group	P-1
a (Å)	10.87930(10)
b (Å)	12.47370(10)
c (Å)	13.47920(10)
α (°)	107.8860(10)
β (°)	99.8030(10)
γ (°)	106.6670(10)
Unit cell volume (Å^3)	1599.30(3)
Z	2
ρ calcg (cm^3)	1.339
μ (mm^{-1})	5.184
F(000)	660.0
Radiation	Cu Kα (λ = 1.54184)
2Θ range for data collection/°	8.496 to 151.494
Index ranges	-13 ≤ h ≤ 13, -15 ≤ k ≤ 15, -16 ≤ l ≤ 16
Reflections collected	59166
Independent reflections	6378 [R_{int} = 0.0764, R_{sigma} = 0.0329]
Data/restraints/parameters	6378/0/373
Goodness-of-fit on F^2	1.129
Final R indexes [I>=2σ (I)]	R_1 = 0.0386, wR_2 = 0.1034
Final R indexes [all data]	R_1 = 0.0415, wR_2 = 0.1046
Largest diff. peak/hole (e Å^{-3})	1.11/-1.12

CCDC 2553626 contains the supplementary crystallographic data for this paper. These data can be obtained free of charge from The Cambridge Crystallographic Data Centre via www.ccdc.cam.ac.uk/structures.

7. Computational data

7.1. General Considerations

DFT calculations were performed using the PBE0-D3(BJ)$_{PCM(THF)}$/def2-TZVP//RI-BP86$_{PCM(THF)}$/def2-SVP level of theory with Gaussian16, C.01.[6] Thermal corrections to enthalpies and entropies were evaluated from standard statistical thermodynamics expressions as implemented in Gaussian using standard temperature and pressure. Free energies at the specified temperature T were evaluated via

$$\Delta G(T) = \Delta H(298.15\ K) - T\ \Delta S(298.15\ K) \qquad (1).$$

This is the same level as used in previous work from our group,[7-12] benchmarked against a series of experimental 3d transition metal hydride bond strengths[13] and including Martin, Hay and Pratt empirical entropy corrections[14] (i.e. 5.09 kcal mol^{-1} per particle evaluated for THF and T = 423.15 K[9]). Ru was described using the Stuttgart-Dresden relativistic small-core pseudopotential[15] in conjunction with the def2-SVP and def2-TZVP valence basis sets. Solvent effects were included implicitly through the polarizable continuum model in its integral equation formalism.[16] For continuity, we report data using THF. Solvent effects were evaluated for selected stationary points by way of single points at the PBE0-D3(BJ)$_{PCM(xylene\text{-}mixture)}$/def2-TZVP//RI-BP86$_{PCM(THF)}$/def2-SVP level (reoptimisation at the RI-BP86$_{PCM(xylene\text{-}mixture)}$ level was not deemed worth the computational effort). As noted previously, the choice of solvent between THF (consistent with previous work[7,12]) and xylene (used experimentally) has little impact upon the overall thermodynamics (compare Figure S73 below and Figure 4 in the main paper). The stereochemistry and conformation of the metal fragment were taken as that observed in the X-ray structures, notably with the CO ligand of the [Ru](H)(CO) moiety *trans* to the MACHO-N atom, and the hydride *trans* to the bound substrates.

Table S2: Comparison between single-point energy in THF, and xylene[a] (in a.u., PBE0-D3(BJ)$_{PCM(Solvent)}$ / def2-TZVP // RI-BP86$_{PCM(THF)}$/def2-SVP level).

Molecule \ Solvent	THF	xylene
**1A**	-2029.06886	-2029.06277
**1B**	-2142.34231	-2142.04176
**1C**	-2218.75835	-2218.74911
**1D**	-2218.75946	-2218.74774
**1E**	-2030.27443	-2030.26538
**1G**	-2332.03991	-2332.02887
**TSB-C**	-2218.72941	-2218.72141
**TSC-D**	-2218.75803	-2218.73096
**TSE-A**	-2106.63445	-2106.62731

[a]Employing the parameters of a mixture of o-, m- and p-xylene.

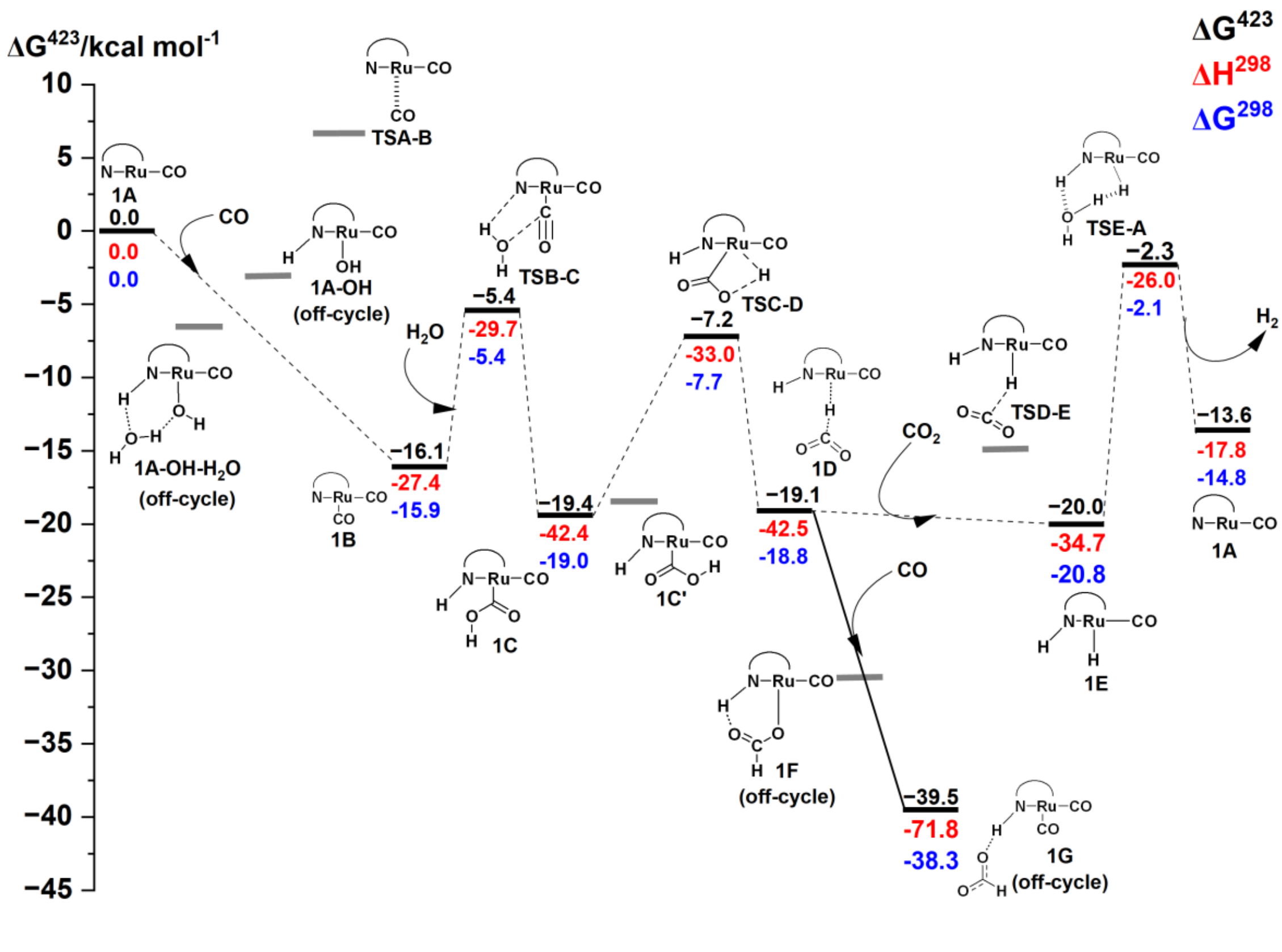

Figure S74: Energy profile showing important mechanistic steps in the water gas shift reaction using an activated Ru-MACHO catalyst, 1A (PBE0-D3(BJ)$_{PCM(xylene-mixture)}$/def2-TZVP//RI-BP86$_{PCM(THF)}$/def2-SVP level). ΔH and ΔG are relative enthalpies and free energies at the given temperature (superscript in K). Only selected stationary points were re-calculated using xylene as the solvent. Intermediates shown in grey were not re-calculated in xylene, and their corresponding energy values are therefore given as n/a. These stationary points are not expected to affect the overall energy span, given the similar relative free energies obtained in THF (see Figure 4 in the main paper) and xylene for the re-calculated species.

7.2. Computational Raw Data

CO
Frequencies, energies and thermodynamic properties:
Lowest Vibrational Mode (cm^{-1}) = 2149.206187
2nd Lowest Vibrational Mode (cm^{-1}) =
E(RB-P86) (h) = -113.22512
Thermal correction to Enthalpy (h) = 0.00820
Thermal correction to Gibbs Free Energy (h) = -0.01425
Total Entropy (cal/Kmol) = 47.251
E(RPBE1PBE) (h) = -113.231032
Optimised cartesian coordinates (Å):
C 0.0000000 0.0000000 -0.6526720
O 0.0000000 0.0000000 0.4895040

H_2O
Frequencies, energies and thermodynamic properties:
Lowest Vibrational Mode (cm^{-1}) = 1609.3992
2nd Lowest Vibrational Mode (cm^{-1}) = 3673.6140
E(RB-P86) (h) = -76.36500
Thermal correction to Enthalpy (h) = 0.02438
Thermal correction to Gibbs Free Energy (h) = 0.00290
Total Entropy (cal/Kmol) = 45.202
E(RPBE1PBE) (h) = -76.38313
Optimised cartesian coordinates (Å):
O 0.0000000 0.0000000 0.1233180
H 0.0000000 0.7574680 -0.4932730
H 0.0000000 -0.7574680 -0.4932730

CO_2
Frequencies, energies and thermodynamic properties:
Lowest Vibrational Mode (cm^{-1}) = 622.8599
2nd Lowest Vibrational Mode (cm^{-1}) =
E(RB-P86) (h) = -188.45623
Thermal correction to Enthalpy (h) = 0.01493
Thermal correction to Gibbs Free Energy (h) = -0.01007
Total Entropy (cal/Kmol) = 52.619
E(RPBE1PBE) (h) = -188.46784
Optimised cartesian coordinates (Å):
C 0.0000000 0.0000000 -0.0001190
O 0.0000000 0.0000000 1.1750910
O 0.0000000 0.0000000 -1.1750020

H_2
Frequencies, energies and thermodynamic properties:
Lowest Vibrational Mode (cm^{-1}) = 4271.1311
2nd Lowest Vibrational Mode (cm^{-1}) =
E(RB-P86) (h) = -1.17254
Thermal correction to Enthalpy (h) = 0.01303
Thermal correction to Gibbs Free Energy (h) = -0.00182

Total Entropy (cal/Kmol) = 31.263
E(RPBE1PBE) (h) = -1.16812
Optimised cartesian coordinates (Å):
H 0.0000000 0.0000000 0.3838560
H 0.0000000 0.0000000 -0.3838560

1A
Frequencies, energies and thermodynamic properties:
Lowest Vibrational Mode (cm^{-1}) = 13.9413
2nd Lowest Vibrational Mode (cm^{-1}) = 15.2297
E(RB-P86) (h) = -2029.32435
Thermal correction to Enthalpy (h) = 0.521925
Thermal correction to Gibbs Free Energy (h) = 0.418537
Total Entropy (cal/Kmol) = 217.598
E(RPBE1PBE) (h) = -2029.06886
Optimised cartesian coordinates (Å):
Ru 0.00001400 -0.22775500 0.18749600
P -2.31017800 -0.03905000 0.44937700
P 2.31019800 -0.03902900 0.44939000
N 0.00001500 0.21474700 2.16819400
O 0.00001000 -1.90669100 -2.34368300
C -3.11421300 1.51618000 -0.15922200
C 1.20927100 0.61948000 2.89637800
H 1.11824400 0.33925500 3.97540000
H 1.33270400 1.73364300 2.88760100
C -3.96628800 2.31447100 0.63573400
H -4.18955700 2.03216500 1.67594700
C 3.48762300 -1.37166500 -0.08056600
C -3.48758100 -1.37170000 -0.08060300
C 3.96606700 2.31467900 0.63570300
H 4.18926600 2.03249900 1.67596500
C -4.82540100 -1.10964000 -0.44989100
H -5.20122200 -0.07453100 -0.46478000
C 3.02198800 -2.70663900 -0.07980200
H 1.97250600 -2.91468100 0.18752200
C 2.45816600 -0.04507100 2.31105300
H 2.47747200 -1.12245300 2.58058100
H 3.40167000 0.40561200 2.68140400
C -2.45812600 -0.04516400 2.31104400
H -3.40164500 0.40546400 2.68142800
H -2.47738600 -1.12255700 2.58053000
C 3.11418700 1.51620400 -0.15927600
C -1.20926000 0.61941900 2.89638200
H -1.33273800 1.73357800 2.88762900
H -1.11822400 0.33917400 3.97539900
C 3.88359600 -3.76114600 -0.42671100
H 3.50881400 -4.79712600 -0.42191400
C 4.82548500 -1.10959300 -0.44969600
H 5.20131300 -0.07448400 -0.46448400

C -3.44202900 3.07257900 -2.02116100
H -3.23261800 3.36522300 -3.06238200
C -2.85271900 1.91291700 -1.49209500
H -2.17262200 1.30926400 -2.11432900
C 0.00001600 -1.23103900 -1.37269500
C -3.02195400 -2.70667700 -0.07971400
H -1.97249900 -2.91471200 0.18772400
C -4.54447600 3.48459100 0.10789100
H -5.20148900 4.10067100 0.74231500
C 4.54416500 3.48482600 0.10781600
H 5.20102100 4.10105200 0.74226000
C 5.68365600 -2.16684700 -0.80283500
H 6.72480000 -1.95024600 -1.09104700
C 2.85281200 1.91277400 -1.49222300
H 2.17287500 1.30897100 -2.11448600
C -4.28733000 3.86399000 -1.22067000
H -4.74223200 4.77888400 -1.63246400
C 5.21653800 -3.49334600 -0.78971700
H 5.89015400 -4.31934700 -1.06829400
C -5.68354300 -2.16691100 -0.80305400
H -6.72465400 -1.95032000 -1.09138900
C 4.28713000 3.86406500 -1.22081000
H 4.74195600 4.77898300 -1.63263500
C -3.88353400 -3.76120000 -0.42664500
H -3.50876000 -4.79718200 -0.42174600
C -5.21643700 -3.49341200 -0.78980400
H -5.89003000 -4.31942600 -1.06839800
C 3.44203700 3.07245900 -2.02133100
H 3.23272300 3.36496600 -3.06261000
H -0.00000900 0.99886400 -0.81085300

1B

Frequencies, energies and thermodynamic properties:
Lowest Vibrational Mode (cm^{-1}) = 9.1275
2nd Lowest Vibrational Mode (cm^{-1}) = 15.1636
E(RB-P86) (h) = -2142.58916
Thermal correction to Enthalpy (h) = 0.53156
Thermal correction to Gibbs Free Energy (h) = 0.42394
Total Entropy (cal/Kmol) = 226.502
E(RPBE1PBE) (h) = -2142.34231
Optimised cartesian coordinates (Å):
Ru -0.0000060 -0.2906000 0.2017240
P -2.3222640 0.0004460 0.4893310
P 2.3222640 0.0003420 0.4893060
O -0.0000630 -0.2196970 -2.8501700
N 0.0000100 -0.0164580 2.3689300
C -1.1797160 0.6497790 2.8888990
H -1.1841760 1.7584440 2.6626720
H -1.2026810 0.5842670 4.0087000

C 1.1798240 0.6496030 2.8889300
H 1.2027920 0.5840210 4.0087270
H 1.1844140 1.7582800 2.6627720
C 2.4699310 0.0314320 2.3413990
H 2.5481760 -1.0335760 2.6478360
H 3.3842070 0.5553080 2.6880250
C -2.4699010 0.0317200 2.3414290
H -3.3841040 0.5557600 2.6879980
H -2.5482920 -1.0332490 2.6479670
C -0.0000530 -0.2981230 -1.6795550
H 0.0000050 1.3717770 0.1765770
C -3.5403270 -1.2698140 -0.0864330
C -4.8877870 -1.2021820 0.3406970
C -3.1431780 -2.3108790 -0.9512680
C -5.8139040 -2.1700820 -0.0808530
H -5.2207530 -0.3871430 1.0035810
C -4.0755100 -3.2746120 -1.3791390
H -2.0985060 -2.3653160 -1.2944370
C -5.4093580 -3.2080320 -0.9421930
H -6.8591050 -2.1118660 0.2622570
H -3.7540660 -4.0811950 -2.0568960
H -6.1382180 -3.9642730 -1.2743820
C -3.0757210 1.5631970 -0.1552040
C -3.3849150 2.6593640 0.6789890
C -3.3085080 1.6716040 -1.5461740
C -3.9193860 3.8405700 0.1310060
H -3.2069220 2.6060680 1.7637100
C -3.8431710 2.8510510 -2.0891450
H -3.0729670 0.8269840 -2.2137680
C -4.1500850 3.9400000 -1.2515380
H -4.1562970 4.6875640 0.7943940
H -4.0209220 2.9197720 -3.1741290
H -4.5685410 4.8654100 -1.6779370
C 3.5402670 -1.2699020 -0.0866130
C 4.8877620 -1.2023030 0.3404100
C 3.1430280 -2.3109410 -0.9514410
C 5.8138360 -2.1701910 -0.0812620
H 5.2207910 -0.3872930 1.0032980
C 4.0753170 -3.2746640 -1.3794300
H 2.0983190 -2.3653760 -1.2944980
C 5.4092060 -3.2081050 -0.9426050
H 6.8590670 -2.1119950 0.2617590
H 3.7538040 -4.0812260 -2.0571780
H 6.1380300 -3.9643410 -1.2748840
C 3.0757550 1.5631340 -0.1550970
C 3.3855340 2.6590150 0.6792540
C 3.3080230 1.6718420 -1.5461320
C 3.9200530 3.8402410 0.1313600
H 3.2079680 2.6054830 1.7640320

C 3.8427430 2.8513030 -2.0890130
H 3.0720110 0.8274510 -2.2138500
C 4.1502310 3.9399710 -1.2512480
H 4.1574150 4.6870130 0.7948700
H 4.0200830 2.9202620 -3.1740490
H 4.5687300 4.8653950 -1.6775760
C 0.0000570 -2.2037300 0.5197750
O 0.0000160 -3.3358300 0.7976260

TSA-B
Frequencies, energies and thermodynamic properties:
Lowest Vibrational Mode (cm^{-1}) = -27.6167
2nd Lowest Vibrational Mode (cm^{-1}) = 16.0632
E(RB-P86) (h) = -2142.54863
Thermal correction to Enthalpy (h) = 0.53061
Thermal correction to Gibbs Free Energy (h) = 0.41997
Total Entropy (cal/Kmol) = 232.848
E(RPBE1PBE) (h) = -2142.30558
Optimised cartesian coordinates (Å):
Ru 0.0362570 -0.1855490 0.1710630
P -2.2693830 0.1279310 0.4090800
P 2.3475040 0.0253250 0.4339450
N 0.0362960 0.4346970 2.1129590
O 0.0389990 -1.9122210 -2.3303160
C -3.0073330 1.7489730 -0.1336690
C 1.2449750 0.8783430 2.8129470
H 1.1445370 0.6844250 3.9100810
H 1.3873570 1.9875700 2.7207900
C -2.1602070 2.8526460 -0.3759710
H -1.0684880 2.7164560 -0.3058580
C 3.5843460 -1.2798100 -0.0208650
C -3.5181450 -1.1027350 -0.1735620
C 3.7785480 2.5273060 0.5136000
H 3.9057900 2.3794980 1.5967020
C -4.4195200 -1.7572060 0.6957550
H -4.4180530 -1.5372980 1.7741570
C 3.1461970 -2.6157310 -0.1540230
H 2.0771900 -2.8496700 -0.0305820
C 2.4880400 0.1528560 2.2883190
H 2.4897040 -0.9020060 2.6359710
H 3.4375810 0.6163420 2.6270090
C -2.4209210 0.1905140 2.2674050
H -3.3606190 0.6795870 2.5978050
H -2.4216030 -0.8636330 2.6163430
C 3.0959720 1.5695730 -0.2680430
C -1.1723780 0.9056580 2.7957210
H -1.2993320 2.0157890 2.6898470
H -1.0870950 0.7224270 3.8957360
C 4.0631680 -3.6392580 -0.4513840

H 3.7084420 -4.6769830 -0.5556100
C 4.9545480 -0.9823660 -0.1997010
H 5.3104910 0.0561880 -0.1089210
C -4.9449900 3.1710610 -0.5901520
H -6.0368440 3.2894700 -0.6786350
C -4.4067220 1.9194610 -0.2440880
H -5.0827890 1.0683270 -0.0640920
C 0.0344060 -1.2273950 -1.3671150
C -3.5527820 -1.4091120 -1.5548200
H -2.8505500 -0.9148360 -2.2450840
C -2.7017470 4.1055080 -0.7163810
H -2.0291410 4.9577960 -0.9035870
C 4.3080840 3.6881840 -0.0826280
H 4.8329810 4.4287370 0.5416880
C 5.8688200 -2.0074150 -0.4993970
H 6.9344400 -1.7643650 -0.6382480
C 2.9536670 1.7972560 -1.6578420
H 2.4082250 1.0672630 -2.2775730
C -4.0942590 4.2671310 -0.8257070
H -4.5181030 5.2466030 -1.0991580
C 5.4257890 -3.3370330 -0.6250690
H 6.1434150 -4.1378770 -0.8644420
C -5.3369280 -2.6985230 0.1917930
H -6.0334350 -3.2017030 0.8812960
C 4.1707930 3.8998660 -1.4646730
H 4.5876860 4.8073430 -1.9295940
C -4.4739180 -2.3434500 -2.0549060
H -4.4895230 -2.5710190 -3.1325840
C -5.3675990 -2.9926100 -1.1820810
H -6.0863930 -3.7295260 -1.5741020
C 3.4944680 2.9483500 -2.2522350
H 3.3804360 3.1080480 -3.3362590
H 0.0977460 1.0009080 -0.8658010
O -0.8074800 -3.9817660 0.2058360
C -0.6906070 -3.1816370 1.0213880

TSB-C
Frequencies, energies and thermodynamic properties:
Lowest Vibrational Mode (cm^{-1}) = -591.7501
2nd Lowest Vibrational Mode (cm^{-1}) = 15.6882
E(RB-P86) (h) = -2218.96337
Thermal correction to Enthalpy (h) = 0.554557
Thermal correction to Gibbs Free Energy (h) = 0.445970
Total Entropy (cal/Kmol) = 228.540
E(RPBE1PBE) (h) = -2218.72941
Optimised cartesian coordinates (Å):
Ru -0.00102300 -0.27361100 0.09735600
P -2.31310900 0.05439600 0.39630100
P 2.31610200 0.01123400 0.38902600

N	0.00477900	-0.03156900	2.28725000
O	-0.01059500	-0.38112300	-2.93015800
C	-3.00371600	1.68240900	-0.15566000
C	1.23536200	0.52799800	2.86856300
H	1.24964700	0.35945300	3.97356800
H	1.27060300	1.63416300	2.71959300
C	-3.51398300	2.64792900	0.73833600
H	-3.52825500	2.45950600	1.82248000
C	3.58483700	-1.16286000	-0.27785800
C	-3.60048100	-1.12875900	-0.21429400
C	3.67641500	2.51009500	0.83738400
H	3.77429700	2.22886100	1.89696600
C	-4.96784500	-0.90611000	0.07286500
H	-5.27823800	-0.01381000	0.64048100
C	3.20035300	-2.21946800	-1.12975700
H	2.14198500	-2.34369300	-1.40255500
C	2.46935800	-0.12582800	2.24079800
H	2.47361600	-1.21670900	2.44967400
H	3.41826800	0.29129100	2.63469000
C	-2.45971400	0.00614900	2.25232100
H	-3.38492100	0.48967800	2.62612300
H	-2.50881100	-1.07293200	2.50924500
C	3.03352000	1.65350700	-0.08204100
C	-1.19009000	0.61968000	2.85078300
H	-1.16055600	1.71987200	2.66302600
H	-1.20903000	0.48825100	3.96066300
C	4.16522700	-3.11407600	-1.62953800
H	3.85410300	-3.93411600	-2.29587200
C	4.94966000	-1.00820700	0.06166300
H	5.27104700	-0.17634500	0.70929400
C	-3.50447200	3.18496100	-2.02160200
H	-3.49756700	3.38966500	-3.10397800
C	-2.99980400	1.96627700	-1.54176100
H	-2.59500200	1.22922300	-2.25405000
C	-0.00755200	-0.36565900	-1.75376000
C	-3.22756200	-2.26452800	-0.96271200
H	-2.16658400	-2.44082100	-1.19295000
C	-4.01220200	3.87322300	0.25478700
H	-4.40421800	4.61804600	0.96555100
C	4.20195800	3.74532000	0.41151800
H	4.69611600	4.40565700	1.14181300
C	5.90864800	-1.90698500	-0.43314700
H	6.96838000	-1.78012000	-0.16022300
C	2.92477200	2.05606700	-1.43388400
H	2.41185700	1.40688400	-2.16156500
C	-4.01121400	4.14394900	-1.12348300
H	-4.40258200	5.10233400	-1.49984200
C	5.51784100	-2.96164800	-1.28038400
H	6.27211500	-3.66287200	-1.67141000

C -5.94130900 -1.81585300 -0.37113800
H -7.00316900 -1.63628400 -0.13900800
C 4.09769400 4.13319400 -0.93445000
H 4.51047800 5.09923600 -1.26562800
C -4.20642000 -3.17047700 -1.41202700
H -3.90401500 -4.05310600 -1.99750700
C -5.56208900 -2.94973000 -1.11504000
H -6.32762800 -3.65980900 -1.46621900
C 3.45881100 3.28307300 -1.85749800
H 3.36923600 3.58101000 -2.91420900
H 0.00239900 1.37663700 -0.01104400
C 0.00072500 -2.28799300 0.36937200
O 0.08109700 -3.39615100 -0.01301600
O -0.32535800 -2.50011400 2.46355100
H 0.47285900 -3.00897600 2.70960000
H -0.08127100 -1.25955100 2.50583300

1C
Frequencies, energies and thermodynamic properties:
Lowest Vibrational Mode (cm^{-1}) = 13.5996
2nd Lowest Vibrational Mode (cm^{-1}) = 16.2644
E(RB-P86) (h) = -2218.98827
Thermal correction to Enthalpy (h) = 0.561874
Thermal correction to Gibbs Free Energy (h) = 0.451851
Total Entropy (cal/Kmol) = 231.562
E(RPBE1PBE) (h) = -2218.75835
Optimised cartesian coordinates (Å):
Ru -0.00000600 -0.21553100 0.13607400
P -2.30749200 0.04616200 0.40703200
P 2.30751100 0.04597100 0.40702700
N 0.00000300 -0.02678800 2.37134200
O 0.00002400 -0.35455700 -2.87331200
C -3.04535300 1.66239200 -0.12998000
C 1.23422800 0.59732800 2.91484600
H 1.27253700 0.47533800 4.02213600
H 1.18171800 1.68587900 2.70238800
C -3.60345000 2.60248700 0.76247800
H -3.63671500 2.40258000 1.84449600
C 3.59564300 -1.14990900 -0.18962400
C -3.59570000 -1.14974800 -0.18937700
C 3.60387800 2.60203100 0.76292500
H 3.63733400 2.40181200 1.84487900
C -4.97003300 -0.90421600 0.04093900
H -5.29072600 0.01609900 0.55580700
C 3.20524600 -2.32106800 -0.87376500
H 2.13716700 -2.52183200 -1.05170300
C 2.47739700 -0.01901100 2.27088600
H 2.54779800 -1.09930900 2.51958000
H 3.40762600 0.45942600 2.63946000

C	-2.47738900	-0.01849900	2.27090400
H	-3.40751200	0.46020800	2.63939800
H	-2.54804900	-1.09874500	2.51975100
C	3.04546200	1.66226400	-0.12968200
C	-1.23408600	0.59763300	2.91480500
H	-1.18134000	1.68615600	2.70226300
H	-1.27241500	0.47573900	4.02210500
C	4.17967800	-3.23696300	-1.31496500
H	3.86630500	-4.14731600	-1.85059800
C	4.97003300	-0.90419100	0.04016800
H	5.29081000	0.01626800	0.55472400
C	-3.55292000	3.17221200	-1.98961200
H	-3.52903200	3.38964800	-3.06932400
C	-3.02154000	1.96389300	-1.51261200
H	-2.57641000	1.24907300	-2.22358700
C	0.00000900	-0.30434900	-1.69390200
C	-3.20541800	-2.32072100	-0.87390400
H	-2.13737500	-2.52136900	-1.05218900
C	-4.12759800	3.81870400	0.28261700
H	-4.55611700	4.54335400	0.99331400
C	4.12810800	3.81830500	0.28329900
H	4.55687100	4.54269600	0.99411200
C	5.93704100	-1.82492000	-0.39577300
H	7.00463800	-1.62639200	-0.20920600
C	3.02143200	1.96414900	-1.51222700
H	2.57605500	1.24958900	-2.22330900
C	-4.10662600	4.10565400	-1.09215300
H	-4.51829100	5.05650300	-1.46605000
C	5.54298000	-2.99340800	-1.07579700
H	6.30282900	-3.71189900	-1.42269700
C	-5.93709700	-1.82494300	-0.39487800
H	-7.00465200	-1.62657000	-0.20790100
C	4.10691400	4.10563500	-1.09138800
H	4.51864500	5.05652700	-1.46510400
C	-4.17990800	-3.23661100	-1.31498600
H	-3.86662800	-4.14681900	-1.85091900
C	-5.54315100	-2.99323700	-1.07530400
H	-6.30305100	-3.71172300	-1.42210400
C	3.55290100	3.17251900	-1.98899800
H	3.52883800	3.39025500	-3.06864500
H	0.00000500	1.45965500	0.16264900
C	-0.00013900	-2.32689200	0.40685800
O	-0.00008100	-3.26482600	-0.39653300
O	-0.00038900	-2.71512500	1.77084300
H	-0.00044400	-3.70328300	1.76682000
H	-0.00011900	-1.03572400	2.62311500

1C‘

Frequencies, energies and thermodynamic properties:

Lowest Vibrational Mode (cm^{-1}) = 15.3146
2nd Lowest Vibrational Mode (cm^{-1}) = 24.2698
E(RB-P86) (h) = -2218.99340
Thermal correction to Enthalpy (h) = 0.562811
Thermal correction to Gibbs Free Energy (h) = 0.455378
Total Entropy (cal/Kmol) = 226.113
E(RPBE1PBE) (h) = -2218.76084
Optimised cartesian coordinates (Å):

Ru	-0.00001900	-0.28951500	0.09966400
P	-2.31044200	0.01491700	0.37846900
P	2.31045500	0.01495900	0.37844500
N	0.00001600	-0.08935600	2.32445500
O	-0.00011900	-0.46861000	-2.91222500
C	-2.98369400	1.67654000	-0.09769000
C	1.23342400	0.50725500	2.89523100
H	1.26411000	0.34437400	3.99757900
H	1.19702200	1.60416800	2.72461500
C	-3.58989900	2.56296500	0.81830300
H	-3.69700400	2.29101500	1.87955700
C	3.65451900	-1.11732600	-0.22979700
C	-3.65442200	-1.11743000	-0.22989000
C	3.58970800	2.56315500	0.81806100
H	3.69679000	2.29132700	1.87934800
C	-5.00717400	-0.70345300	-0.23924000
H	-5.27602900	0.31655100	0.07797900
C	3.33285700	-2.42346300	-0.65713500
H	2.28428700	-2.75496800	-0.65750400
C	2.47207500	-0.10283000	2.23842500
H	2.52568100	-1.19150000	2.45277600
H	3.40977800	0.35046700	2.61905600
C	-2.47205800	-0.10303900	2.23843100
H	-3.40977100	0.35018000	2.61913200
H	-2.52560200	-1.19173200	2.45268000
C	2.98358800	1.67659400	-0.09786000
C	-1.23345700	0.50706600	2.89528500
H	-1.19714200	1.60400100	2.72479100
H	-1.26413100	0.34407100	3.99761700
C	4.34639900	-3.30371100	-1.07819800
H	4.08060700	-4.31998700	-1.41010400
C	5.00725400	-0.70329700	-0.23900600
H	5.27603800	0.31672200	0.07822600
C	-3.35885200	3.31109000	-1.88132000
H	-3.26460700	3.59854100	-2.94059300
C	-2.86863000	2.06779500	-1.45257700
H	-2.38181800	1.39665800	-2.17843200
C	-0.00008100	-0.41489000	-1.73018100
C	-3.33268100	-2.42355500	-0.65720500
H	-2.28409500	-2.75500900	-0.65752600
C	-4.06926800	3.81593600	0.38860200

H -4.53398900 4.49869500 1.11769600
C 4.06901000 3.81611300 0.38824700
H 4.53365700 4.49898000 1.11728900
C 6.01710000 -1.58563000 -0.65913100
H 7.06690000 -1.25128200 -0.66104500
C 2.86856700 2.06769600 -1.45279500
H 2.38183200 1.39645100 -2.17860100
C -3.95861100 4.19158100 -0.96018800
H -4.33586700 5.17079400 -1.29515500
C 5.68918300 -2.88785500 -1.08015400
H 6.48174500 -3.57649600 -1.41376800
C -6.01695300 -1.58582200 -0.65945000
H -7.06676400 -1.25151200 -0.66145900
C 3.95838200 4.19161000 -0.96058700
H 4.33558700 5.17081200 -1.29564200
C -4.34615200 -3.30383900 -1.07836000
H -4.08029100 -4.32010100 -1.41025300
C -5.68895300 -2.88803500 -1.08044100
H -6.48145500 -3.57670400 -1.41413800
C 3.35872400 3.31097800 -1.88164900
H 3.26450600 3.59830900 -2.94095700
H -0.00002500 1.37826400 0.05351100
C 0.00006100 -2.35203800 0.63322700
O 0.00012300 -2.77363300 1.80257100
O 0.00008500 -3.36799100 -0.32502300
H -0.00001500 -2.90728900 -1.18994700
H 0.00010400 -1.12692000 2.51480200

TSC-D
Frequencies, energies and thermodynamic properties:
Lowest Vibrational Mode (cm^{-1}) = -29.1244
2nd Lowest Vibrational Mode (cm^{-1}) = 17.1788
E(RB-P86) (h) = -2218.97171
Thermal correction to Enthalpy (h) = 0.558827
Thermal correction to Gibbs Free Energy (h) = 0.451831
Total Entropy (cal/Kmol) = 225.191
E(RPBE1PBE) (h) = -2218.74033
Optimised cartesian coordinates (Å):
Ru -0.01886900 -0.20157800 -0.03564300
P -2.32980900 -0.03616800 0.35003700
P 2.28195300 0.06852000 0.30281700
N -0.14108500 0.77446200 2.00678700
O -0.00916200 -0.93238200 -2.95530500
C -3.04950500 1.64955100 0.04690700
C 1.18379100 1.09213200 2.64031500
H 1.08633900 1.05232000 3.74694700
H 1.43643200 2.13964700 2.37622400
C -3.63371200 2.44536300 1.05569500
H -3.70939700 2.07709400 2.09020500

C	3.59689200	-1.18290700	-0.09902100
C	-3.66054500	-1.16220400	-0.29001600
C	3.82803100	2.49155400	0.54414700
H	3.98387400	2.24463800	1.60587300
C	-4.95856200	-0.69006200	-0.58983600
H	-5.20018800	0.37717600	-0.47155000
C	3.23379500	-2.48380900	-0.50921500
H	2.17161300	-2.75408600	-0.59687100
C	2.29545200	0.15170000	2.16982500
H	2.07835500	-0.88924100	2.48933900
H	3.28134400	0.44764000	2.58435200
C	-2.41565600	-0.30629700	2.20764900
H	-3.30799200	0.18919900	2.64061100
H	-2.58284400	-1.39752500	2.30617700
C	3.06609800	1.64336500	-0.28979100
C	-1.10129400	0.08592100	2.92372500
H	-1.31144200	0.74197100	3.79721700
H	-0.58891400	-0.82918000	3.27200400
C	4.22422600	-3.44200000	-0.79300500
H	3.92549900	-4.45308600	-1.11251500
C	4.96874100	-0.85602100	0.01516600
H	5.27463600	0.15783600	0.31772300
C	-3.49192800	3.43334800	-1.57170900
H	-3.43362000	3.81511600	-2.60336100
C	-2.97659100	2.16364100	-1.27032500
H	-2.50078000	1.56581200	-2.06424600
C	-0.00773800	-0.68355100	-1.79897300
C	-3.37354600	-2.53693300	-0.45335200
H	-2.36596000	-2.91943500	-0.22842400
C	-4.13191000	3.72890900	0.75571500
H	-4.57626600	4.34004100	1.55739000
C	4.40326700	3.66970700	0.02970900
H	4.98829800	4.32474900	0.69484900
C	5.95485800	-1.81650300	-0.26623800
H	7.01930100	-1.54825000	-0.17221200
C	2.89236100	1.99733800	-1.64793600
H	2.28115100	1.35758100	-2.30440800
C	-4.06715800	4.22335500	-0.55686400
H	-4.46036500	5.22526300	-0.79123400
C	5.58505000	-3.11215300	-0.67244700
H	6.35933100	-3.86263900	-0.89818700
C	-5.95027400	-1.57878600	-1.04246800
H	-6.95741200	-1.19680900	-1.27388100
C	4.23434500	4.00628200	-1.32379700
H	4.68643700	4.92695500	-1.72558200
C	-4.36987900	-3.42166800	-0.90071200
H	-4.13240100	-4.49056100	-1.02191100
C	-5.65964900	-2.94550500	-1.19859100
H	-6.43757400	-3.63935900	-1.55486600

C 3.47987300 3.16397900 -2.16286100
H 3.33940000 3.42284700 -3.22439700
H -0.04983800 1.39192800 -0.54588800
C 0.09339800 -2.14266800 0.87005200
O 0.31669600 -2.43695700 2.04664200
O -0.07401500 -3.27304400 0.03609100
H -0.22601100 -2.90615800 -0.85986200
H -0.55687400 1.68004800 1.74853000

1D
Frequencies, energies and thermodynamic properties:
Lowest Vibrational Mode (cm^{-1}) = 17.6142
2nd Lowest Vibrational Mode (cm^{-1}) = 22.5724
E(RB-P86) (h) = -2218.99452
Thermal correction to Enthalpy (h) = 0.560409
Thermal correction to Gibbs Free Energy (h) = 0.450801
Total Entropy (cal/Kmol) = 230.688
E(RPBE1PBE) (h) = -2218.75946
Optimised cartesian coordinates (Å):
Ru -0.00000600 -0.12583900 0.06598500
P -2.31690700 0.07585800 0.35707200
P 2.31688400 0.07588300 0.35707800
N -0.00000800 -0.06166900 2.27774600
O 0.00000700 -0.26655100 -2.94802500
C -3.05586100 1.71796000 -0.07491600
C 1.23808000 0.50013100 2.88267000
H 1.25597300 0.29312600 3.97742300
H 1.21971600 1.60347300 2.75614200
C -3.68147100 2.56239800 0.86756900
H -3.77022500 2.26205500 1.92269200
C 3.54859200 -1.12688400 -0.32669300
C -3.54862900 -1.12690900 -0.32667900
C 3.68147900 2.56240600 0.86756900
H 3.77025700 2.26205000 1.92268700
C -4.93007900 -0.82530600 -0.34850200
H -5.28901100 0.15173100 0.01255900
C 3.10410800 -2.37775400 -0.80900100
H 2.03120600 -2.62629400 -0.80005400
C 2.47298400 -0.10764800 2.21314000
H 2.50732000 -1.20259000 2.39655100
H 3.41522400 0.31909400 2.61262600
C -2.47300800 -0.10767800 2.21313600
H -3.41524700 0.31906800 2.61262200
H -2.50736000 -1.20262000 2.39654100
C 3.05583800 1.71798500 -0.07491000
C -1.23810700 0.50009500 2.88267800
H -1.21975800 1.60344000 2.75617300
H -1.25600000 0.29306800 3.97742700
C 4.03150200 -3.31603600 -1.29763300

H	3.67405500	-4.28858000	-1.67121100
C	4.93004500	-0.82529600	-0.34851200
H	5.28899000	0.15172700	0.01257700
C	-3.49502900	3.38132700	-1.81604200
H	-3.41861300	3.69693200	-2.86860500
C	-2.96235800	2.14413800	-1.42127700
H	-2.46447000	1.50433600	-2.16755800
C	0.00000100	-0.21905000	-1.77103300
C	-3.10416100	-2.37779800	-0.80895000
H	-2.03126000	-2.62634600	-0.80000700
C	-4.20485200	3.80815300	0.47074000
H	-4.68655900	4.45777600	1.21866900
C	4.20486000	3.80816100	0.47074200
H	4.68659100	4.45777200	1.21866600
C	5.85178900	-1.76615000	-0.83790800
H	6.92599500	-1.52198800	-0.85085700
C	2.96230600	2.14417900	-1.42126400
H	2.46439300	1.50439100	-2.16754000
C	-4.11600600	4.21886900	-0.86958300
H	-4.52752300	5.19282400	-1.17831600
C	5.40421300	-3.01292800	-1.31344700
H	6.12796500	-3.74755200	-1.70089200
C	-5.85183500	-1.76616500	-0.83786700
H	-6.92603800	-1.52199100	-0.85081900
C	4.11598500	4.21889400	-0.86957400
H	4.52750300	5.19285000	-1.17830600
C	-4.03156700	-3.31608500	-1.29755100
H	-3.67413300	-4.28864300	-1.67110400
C	-5.40427500	-3.01296200	-1.31337000
H	-6.12803700	-3.74758900	-1.70079100
C	3.49497700	3.38136800	-1.81602800
H	3.41853800	3.69698700	-2.86858500
H	-0.00000900	1.47249500	-0.02924300
C	0.00016200	-3.03380000	1.03285200
O	0.00022800	-2.83340700	2.26549200
O	0.00013700	-4.02080300	0.29926600
H	0.00007900	-1.97801700	0.35965000
H	0.00002200	-1.11011200	2.45507300

TSD-E

Frequencies, energies and thermodynamic properties:

Lowest Vibrational Mode (cm^{-1}) = -230.5702

2nd Lowest Vibrational Mode (cm^{-1}) = 16.7072

E(RB-P86) (h) = -2218.98273

Thermal correction to Enthalpy (h) = 0.557600

Thermal correction to Gibbs Free Energy (h) = 0.447297

Total Entropy (cal/Kmol) = 232.151

E(RPBE1PBE) (h) = -2218.74630

Optimised cartesian coordinates (Å):

Ru	-0.00000100	-0.00621300	0.06238100
P	-2.30498800	0.12367400	0.35655600
P	2.30498700	0.12367400	0.35655600
N	-0.00000100	-0.03568600	2.30092600
O	0.00000000	-0.00420600	-2.95028100
C	-3.15561000	1.71443600	-0.07935600
C	1.23885700	0.53283100	2.89622700
H	1.26711300	0.33377700	3.99261500
H	1.20457000	1.63360700	2.75681100
C	-3.74975600	2.57310300	0.87028900
H	-3.75603600	2.31250600	1.93978200
C	3.49904700	-1.13294200	-0.31174200
C	-3.49904800	-1.13294300	-0.31174200
C	3.74975100	2.57310600	0.87029000
H	3.75602600	2.31251100	1.93978300
C	-4.89740100	-0.94984200	-0.20517600
H	-5.30278000	-0.03257500	0.25179000
C	2.99927300	-2.30412900	-0.91954700
H	1.90914700	-2.43995100	-1.00370100
C	2.47205900	-0.06429200	2.21565100
H	2.52341800	-1.15848500	2.40008700
H	3.41099800	0.37238600	2.61234300
C	-2.47206000	-0.06429200	2.21565100
H	-3.41099900	0.37238500	2.61234300
H	-2.52341900	-1.15848600	2.40008700
C	3.15561000	1.71443700	-0.07935600
C	-1.23885800	0.53283000	2.89622700
H	-1.20457200	1.63360600	2.75681000
H	-1.26711400	0.33377800	3.99261500
C	3.88483100	-3.28297600	-1.40741500
H	3.48407500	-4.19221500	-1.88325700
C	4.89740000	-0.94984200	-0.20517200
H	5.30277900	-0.03257700	0.25179800
C	-3.76805300	3.29607100	-1.84581300
H	-3.77031500	3.57439800	-2.91173600
C	-3.16595200	2.09381200	-1.44282800
H	-2.69171700	1.44518400	-2.19733300
C	0.00000000	-0.00225000	-1.76778700
C	-2.99927400	-2.30413100	-0.91954400
H	-1.90914700	-2.43995500	-1.00369500
C	-4.34510300	3.78361800	0.46518400
H	-4.80166500	4.44366100	1.22000300
C	4.34509900	3.78362000	0.46518500
H	4.80165700	4.44366600	1.22000400
C	5.77869000	-1.93135800	-0.68946300
H	6.86656500	-1.78102600	-0.60033700
C	3.16595700	2.09381000	-1.44282800
H	2.69172600	1.44517900	-2.19733400
C	-4.35891600	4.14676500	-0.89162500

H -4.82601200 5.09316300 -1.20713000
C 5.27392100 -3.09949600 -1.29209200
H 5.96685500 -3.86485300 -1.67673500
C -5.77869100 -1.93135800 -0.68946700
H -6.86656600 -1.78102400 -0.60034400
C 4.35891700 4.14676400 -0.89162500
H 4.82601400 5.09316200 -1.20713000
C -3.88483200 -3.28297900 -1.40741100
H -3.48407600 -4.19222000 -1.88325000
C -5.27392200 -3.09949700 -1.29209200
H -5.96685600 -3.86485400 -1.67673500
C 3.76805900 3.29606800 -1.84581400
H 3.77032500 3.57439200 -2.91173700
H -0.00000200 1.65696300 0.17913100
C 0.00000400 -3.41425200 1.11232000
O 0.00000400 -3.04318700 2.24715400
O 0.00000400 -4.14575500 0.17862100
H 0.00000100 -1.72990300 0.17946200
H 0.00000000 -1.05619300 2.48832400

1E

Frequencies, energies and thermodynamic properties:

Lowest Vibrational Mode (cm^{-1}) = 17.1986

2nd Lowest Vibrational Mode (cm^{-1}) = 25.6509

E(RB-P86) (h) = -2030.52663

Thermal correction to Enthalpy (h) = 0.542594

Thermal correction to Gibbs Free Energy (h) = 0.441834

Total Entropy (cal/Kmol) = 212.066

E(RPBE1PBE) (h) = -2030.27443

Optimised cartesian coordinates (Å):

Ru -0.00000100 -0.16773800 0.15471500
P -2.30343800 -0.06767000 0.44153600
P 2.30343700 -0.06766800 0.44153900
N 0.00000000 -0.05308000 2.40387500
O -0.00001700 -0.34989600 -2.85123900
C -3.21852500 1.43905100 -0.14336300
C 1.24009500 0.56537100 2.94263800
H 1.27171400 0.46886600 4.05279400
H 1.20267800 1.64740100 2.69831000
C -3.92710100 2.31394800 0.70793200
H -3.98270400 2.12376900 1.79074300
C 3.45944200 -1.43109600 -0.08043500
C -3.45944300 -1.43109800 -0.08044700
C 3.92708400 2.31396100 0.70791800
H 3.98267800 2.12379700 1.79073200
C -4.86180100 -1.25187200 -0.10224600
H -5.29859200 -0.27329800 0.15441000
C 2.91832700 -2.68642800 -0.43047600
H 1.82155700 -2.80802800 -0.41856000

C	2.46929700	-0.09071500	2.31351500
H	2.52287300	-1.16451800	2.59465300
H	3.40895300	0.37682800	2.67103600
C	-2.46929600	-0.09073500	2.31351300
H	-3.40895700	0.37679300	2.67104100
H	-2.52286000	-1.16454100	2.59464200
C	3.21852400	1.43904900	-0.14337300
C	-1.24010200	0.56536100	2.94263700
H	-1.20269400	1.64739100	2.69830900
H	-1.27172000	0.46885600	4.05279300
C	3.76625000	-3.75064300	-0.78754100
H	3.33333300	-4.72584000	-1.06296900
C	4.86180100	-1.25186600	-0.10221200
H	5.29858500	-0.27329200	0.15445700
C	-3.82649200	2.85482800	-2.04795000
H	-3.78106800	3.06190300	-3.12905600
C	-3.17027800	1.72800000	-1.52822800
H	-2.60538000	1.06847800	-2.20684800
C	-0.00000400	-0.27311200	-1.66930600
C	-2.91832100	-2.68643300	-0.43047100
H	-1.82155000	-2.80803000	-0.41854400
C	-4.57549100	3.45060600	0.18670800
H	-5.12003400	4.12509000	0.86677800
C	4.57547200	3.45061700	0.18668700
H	5.12000100	4.12511500	0.86675400
C	5.70664800	-2.31764400	-0.45806800
H	6.79829900	-2.16834300	-0.47136500
C	3.17030000	1.72797300	-1.52824400
H	2.60541900	1.06843500	-2.20686300
C	-4.53078100	3.72210900	-1.19076100
H	-5.03973500	4.61041800	-1.59738100
C	5.16083500	-3.56897400	-0.80112100
H	5.82473200	-4.40118700	-1.08515200
C	-5.70664200	-2.31765400	-0.45810600
H	-6.79829200	-2.16835400	-0.47142000
C	4.53077800	3.72210000	-1.19078700
H	5.03973100	4.61040700	-1.59741300
C	-3.76623700	-3.75065300	-0.78753900
H	-3.33331400	-4.72585000	-1.06295400
C	-5.16082200	-3.56898600	-0.80114100
H	-5.82471400	-4.40120200	-1.08517500
C	3.82651100	2.85479900	-2.04797400
H	3.78110200	3.06185600	-3.12908300
H	0.00000000	1.51824300	0.22725900
H	0.00000300	-1.04672700	2.68182400
H	0.00000600	-1.85238500	0.35490900

TSE-A

Frequencies, energies and thermodynamic properties:

Lowest Vibrational Mode (cm^{-1}) = -877.0059
2nd Lowest Vibrational Mode (cm^{-1}) = 13.5361
E(RB-P86) (h) = -2106.87833
Thermal correction to Enthalpy (h) = 0.562320
Thermal correction to Gibbs Free Energy (h) = 0.455903
Total Entropy (cal/Kmol) = 223.974
E(RPBE1PBE) (h) = -2106.63445
Optimised cartesian coordinates (Å):

Ru	0.00132800	-0.28120000	0.08264100
P	-2.30494300	-0.04596500	0.39601400
P	2.31223400	-0.08418400	0.38149400
N	0.00603100	-0.16662000	2.28581100
O	-0.01347200	-0.24422600	-2.94566700
C	-3.06061100	1.56133900	-0.13296000
C	1.23183200	0.38892700	2.87764000
H	1.25178900	0.19717900	3.97990600
H	1.26449600	1.50040700	2.75654100
C	-3.52633300	2.53437600	0.77728000
H	-3.47963100	2.35654400	1.86242400
C	3.53693900	-1.31326000	-0.27846600
C	-3.54693700	-1.27661900	-0.22295900
C	3.71375300	2.39186200	0.84380300
H	3.77601800	2.11953500	1.90840100
C	-4.93124800	-1.08343600	-0.00580200
H	-5.29047300	-0.17622500	0.50682700
C	3.07488800	-2.49880100	-0.88879200
H	1.99103000	-2.66331900	-0.99765000
C	2.47251700	-0.23942000	2.23558500
H	2.49409100	-1.33278300	2.43013000
H	3.42062700	0.18149700	2.62814200
C	-2.46025300	-0.12793500	2.25454400
H	-3.38493000	0.35427000	2.63228000
H	-2.52394200	-1.21106500	2.49090600
C	3.08788700	1.53278000	-0.08511900
C	-1.18776000	0.46342100	2.87105300
H	-1.16555900	1.57178300	2.72341700
H	-1.20845300	0.29728200	3.97731700
C	3.98909200	-3.45921200	-1.35965300
H	3.61678000	-4.37904900	-1.83807500
C	4.92917400	-1.09789400	-0.15324300
H	5.30657600	-0.17004300	0.30634600
C	-3.66832500	3.04399500	-1.98406500
H	-3.72048200	3.23809300	-3.06723100
C	-3.13248800	1.83227500	-1.52003700
H	-2.76231200	1.08980500	-2.24562700
C	-0.00862200	-0.28638000	-1.76740500
C	-3.10927200	-2.43506600	-0.89860200
H	-2.03192400	-2.58218900	-1.07485200
C	-4.05664500	3.75227700	0.30954600

H -4.41409300 4.50232600 1.03302500
C 4.26704800 3.61637000 0.42189900
H 4.74843700 4.27776800 1.15980400
C 5.83987900 -2.05991900 -0.62163800
H 6.92245700 -1.88319900 -0.51850000
C 3.02293300 1.92458800 -1.44338300
H 2.52274600 1.27462100 -2.17928000
C -4.13139800 4.00942900 -1.06946500
H -4.54764900 4.96212400 -1.43348200
C 5.37158700 -3.24241000 -1.22545400
H 6.08750900 -3.99296400 -1.59666000
C -5.85798400 -2.04177700 -0.44874300
H -6.93404000 -1.88400600 -0.27247300
C 4.20709200 3.99213800 -0.93027900
H 4.64156600 4.94963400 -1.25864800
C -4.04013000 -3.39091700 -1.34607500
H -3.68774800 -4.29017000 -1.87591900
C -5.41389600 -3.19721100 -1.11997100
H -6.14272200 -3.94529700 -1.47054600
C 3.58426200 3.14080400 -1.86315500
H 3.52877300 3.42950600 -2.92482300
H 0.01228300 1.33236000 0.06115300
H -0.07610100 -1.49234200 2.48468200
H 0.01822400 -2.10726800 0.01552600
O -0.25390600 -2.66897900 2.39479000
H -0.07030900 -2.23394800 0.89326200
H 0.58411400 -3.09982300 2.65468900

1A-OH
Frequencies, energies and thermodynamic properties:
Lowest Vibrational Mode (cm^{-1}) = 6.2610
2nd Lowest Vibrational Mode (cm^{-1}) = 17.7791
E(RB-P86) (h) = -2105.71761
Thermal correction to Enthalpy (h) = 0.549702
Thermal correction to Gibbs Free Energy (h) = 0.443517
Total Entropy (cal/Kmol) = 223.486
E(RPBE1PBE) (h) = -2105.47753
Optimised cartesian coordinates (Å):
Ru -0.00167000 -0.13143400 0.13215500
P -2.32656500 -0.02065500 0.41162100
P 2.29537600 0.05122100 0.43248700
N -0.01529400 0.13524000 2.33658100
O -0.00399400 -0.52204200 -2.85453100
C -3.24411700 1.51025700 -0.08660700
C 1.20136200 0.74453000 2.91626900
H 1.21627500 0.61219400 4.02378200
H 1.17314900 1.83723400 2.71737200
C -4.40992800 1.94736700 0.58437400
H -4.80531800 1.38564400 1.44558400

C	3.44915900	-1.31862600	-0.05609200
C	-3.39619700	-1.41305100	-0.18314800
C	3.67671500	2.57535100	0.60948500
H	3.59636800	2.50693200	1.70516800
C	-4.68395200	-1.23941600	-0.73563300
H	-5.11896500	-0.23236400	-0.82975800
C	2.92301800	-2.45609600	-0.70597000
H	1.84466000	-2.49362400	-0.93435400
C	2.45213700	0.10529700	2.30194800
H	2.52380700	-0.95949500	2.61074600
H	3.38057300	0.60455100	2.64595800
C	-2.46996600	-0.10472600	2.28250700
H	-3.43618800	0.28854600	2.65708100
H	-2.43345700	-1.18634300	2.53190500
C	3.17151500	1.54869800	-0.21672500
C	-1.28378600	0.62635100	2.91881300
H	-1.35291400	1.72127200	2.74092000
H	-1.29125100	0.47195100	4.02357900
C	3.76687100	-3.52308900	-1.06663800
H	3.34639800	-4.40407500	-1.57701900
C	4.83359200	-1.26030600	0.22469600
H	5.26261500	-0.37315100	0.71854600
C	-3.45764800	3.40341900	-1.62348300
H	-3.07919500	3.97122600	-2.48832500
C	-2.77395300	2.25300300	-1.19283300
H	-1.85493000	1.92932500	-1.70549200
C	-0.00065300	-0.38402200	-1.67902600
C	-2.83773000	-2.71247400	-0.09290800
H	-1.80576900	-2.81526000	0.31124600
C	-5.08662300	3.10474300	0.15898000
H	-5.98985500	3.43654000	0.69547300
C	4.29160900	3.70859800	0.04312500
H	4.67988100	4.50200300	0.70153300
C	5.67345200	-2.32837500	-0.13324000
H	6.75120500	-2.27492900	0.08915400
C	3.28671700	1.68109100	-1.62120600
H	2.88430300	0.89648900	-2.28260500
C	-4.61314000	3.83386400	-0.94671500
H	-5.14411700	4.74020000	-1.27886600
C	5.14143800	-3.46113200	-0.77912800
H	5.80306200	-4.29533000	-1.06195000
C	-5.41752700	-2.35684800	-1.17647900
H	-6.42115900	-2.21338900	-1.60833200
C	4.41108800	3.82686500	-1.35160600
H	4.89387100	4.71320900	-1.79280900
C	-3.58336700	-3.82132500	-0.52950900
H	-3.14950300	-4.83221700	-0.45661400
C	-4.87252300	-3.64865700	-1.06936500
H	-5.45017100	-4.52125200	-1.41483600

C 3.90694400 2.80815500 -2.18300400
H 3.99312400 2.89428100 -3.27788500
H -0.00034000 1.46581300 -0.13297100
O -0.05375000 -2.14371800 1.04934300
H 0.73631200 -2.64774100 0.77673300
H 0.03082900 -0.92091900 2.39070600

1A-OH-H_2O
Frequencies, energies and thermodynamic properties:
Lowest Vibrational Mode (cm^{-1}) = 15.9214
2nd Lowest Vibrational Mode (cm^{-1}) = 22.2684
E(RB-P86) (h) = -2182.31183
Thermal correction to Enthalpy (h) = 0.577388
Thermal correction to Gibbs Free Energy (h) = 0.468910
Total Entropy (cal/Kmol) = 228.311
E(RPBE1PBE) (h) = -2181.88893
Optimised cartesian coordinates (Å):
Ru -0.00262800 -0.02392200 0.06357900
P -2.30122800 -0.06398800 0.37144900
P 2.26852300 0.17660500 0.36859600
N 0.00344500 -0.02094400 2.29374800
O -0.03915200 0.32903900 -2.92093400
C -3.22538100 1.49035700 0.03496900
C 1.16255900 0.72324800 2.83700400
H 1.19627600 0.63614800 3.94783100
H 1.04260800 1.79851500 2.58675700
C -4.12637800 2.07205400 0.95035600
H -4.30825500 1.60509900 1.92992100
C 3.33294700 -1.20342300 -0.22429400
C -3.36320400 -1.36319300 -0.37972200
C 4.59190000 1.70406100 -0.22669800
H 5.16866800 0.80895100 0.05549500
C -4.72746300 -1.15595100 -0.67520800
H -5.19463000 -0.17926100 -0.47343700
C 3.13985800 -1.63458700 -1.55721200
H 2.38330100 -1.13804000 -2.18533000
C 2.44987300 0.16407400 2.23052300
H 2.56773400 -0.89740700 2.53262500
H 3.34588500 0.72370800 2.56426200
C -2.42555600 -0.35922700 2.20714100
H -3.41856000 -0.10091100 2.62554100
H -2.27984900 -1.45178300 2.34315200
C 3.17997400 1.67570700 -0.19500900
C -1.28690200 0.40484100 2.88279800
H -1.40432200 1.49961900 2.73226600
H -1.29072100 0.21550200 3.98104900
C 3.90062900 -2.69318100 -2.07810900
H 3.73997900 -3.02157900 -3.11677500
C 4.29648700 -1.85176400 0.57719300

H	4.46615300	-1.53150500	1.61638600
C	-3.70667700	3.28677900	-1.55014600
H	-3.53922300	3.75935300	-2.53067400
C	-3.01623300	2.11167900	-1.21702700
H	-2.30196000	1.67197000	-1.93015600
C	-0.02271000	0.16069300	-1.74956400
C	-2.75486900	-2.61000800	-0.65524200
H	-1.67343900	-2.73559400	-0.43395900
C	-4.80676700	3.25852800	0.62001500
H	-5.50304100	3.70735900	1.34575300
C	5.26304400	2.86972500	-0.62983100
H	6.36403400	2.88565700	-0.65530300
C	5.05470800	-2.91414500	0.05322600
H	5.80385000	-3.41369900	0.68733900
C	2.45005900	2.81912100	-0.57878600
H	1.34898600	2.77240500	-0.55972900
C	-4.60137100	3.86572300	-0.63013100
H	-5.13607000	4.79325500	-0.88813800
C	4.85857400	-3.33664100	-1.27284800
H	5.45094000	-4.17105600	-1.67947200
C	-5.48738400	-2.19747500	-1.23480900
H	-6.55181000	-2.03418200	-1.46695600
C	4.53131700	4.01194500	-1.00617000
H	5.06015900	4.92355900	-1.32610100
C	-3.52708200	-3.64503300	-1.21049500
H	-3.05816800	-4.61849800	-1.42706900
C	-4.88946200	-3.44305900	-1.50044900
H	-5.48730600	-4.25688400	-1.94091200
C	3.12590100	3.98439600	-0.98074700
H	2.55001300	4.87377300	-1.28150400
H	-0.13236900	1.57983900	0.20788900
O	0.15463800	-2.22955200	0.17811500
H	0.95411400	-2.49877600	-0.31429000
H	0.13242900	-1.04887100	2.54222800
O	0.22287600	-2.74019300	2.62885600
H	1.11850100	-3.05450900	2.85638500
H	0.23896000	-2.63800600	1.56790800

1F

Frequencies, energies and thermodynamic properties:

Lowest Vibrational Mode (cm^{-1}) = 15.3506

2nd Lowest Vibrational Mode (cm^{-1}) = 23.4806

E(RB-P86) (h) = -2219.01298

Thermal correction to Enthalpy (h) = 0.562772

Thermal correction to Gibbs Free Energy (h) = 0.454460

Total Entropy (cal/Kmol) = 227.962

E(RPBE1PBE) (h) = -2218.77896

Optimised cartesian coordinates (Å):

H	-0.09143900	1.49435400	0.13140200

Ru	-0.00880000	-0.09978100	0.04270600
O	0.09503400	-2.35471200	0.04719200
P	-2.33190200	-0.02026800	0.34695200
P	2.29875600	0.14024400	0.35899900
C	-2.47206400	-0.31993300	2.18886700
C	2.45954400	0.06437400	2.22939400
H	-2.43079600	-1.42300500	2.31232000
H	2.60540700	-0.99798200	2.51199900
H	-3.44167000	0.01630400	2.60967100
H	3.34513500	0.63302400	2.57771700
C	-1.28276500	0.34029900	2.89040100
C	1.16945000	0.59719000	2.85494500
H	-1.29242200	0.09004600	3.97689100
H	1.19818000	0.45088200	3.96006000
H	-1.34894000	1.44715100	2.81002900
H	1.06166800	1.68781900	2.66989200
N	-0.00635700	-0.09650100	2.27431500
H	0.11654000	-1.14255500	2.42254600
C	-0.01305100	0.03070700	-1.78625800
O	-0.02119400	0.15696700	-2.95939800
C	0.24968700	-3.13176200	1.05012700
O	0.37371700	-2.82521700	2.26441800
H	0.27582000	-4.22969300	0.78078900
C	-3.15903800	1.60444900	0.01763600
C	-3.05580300	2.13835500	-1.28905800
C	-3.86784700	2.33239200	0.99779500
C	-3.65941000	3.36491900	-1.60862700
H	-2.49311500	1.59204500	-2.06295100
C	-4.46236800	3.56832300	0.67780600
H	-3.96649100	1.94670700	2.02395300
C	-4.36286000	4.08553000	-0.62443900
H	-3.57395600	3.76450600	-2.63163000
H	-5.00803900	4.12611600	1.45545100
H	-4.83007300	5.05143700	-0.87360900
C	-3.50995300	-1.23894100	-0.40266200
C	-4.90359100	-1.00374300	-0.43720900
C	-2.99158100	-2.44059500	-0.93591200
C	-5.76769100	-1.96346500	-0.99195200
H	-5.31718900	-0.06434600	-0.03665000
C	-3.86272900	-3.39657500	-1.48987300
H	-1.90373700	-2.61995200	-0.90401500
C	-5.24890300	-3.16082700	-1.51949200
H	-6.85251300	-1.77235100	-1.01635000
H	-3.45251800	-4.33062500	-1.90613200
H	-5.92786600	-3.90911000	-1.95879500
C	3.47146000	-1.14441200	-0.27886100
C	4.17693100	-0.92223900	-1.48487800
C	3.58020700	-2.40567200	0.35209800
C	4.99020300	-1.92912900	-2.03261700

H 4.09625000 0.04773600 -2.00028700
C 4.40020000 -3.40778200 -0.19563700
H 3.01745000 -2.62135400 1.27376500
C 5.10909500 -3.17316100 -1.38729200
H 5.53595800 -1.73709800 -2.97017400
H 4.48026300 -4.38141000 0.31357200
H 5.74973000 -3.96051800 -1.81507400
C 3.13698600 1.73171900 -0.09432200
C 4.48848500 1.95435800 0.26373400
C 2.43174200 2.74448400 -0.77727300
C 5.11639600 3.16992000 -0.05337000
H 5.06125600 1.17122400 0.78704000
C 3.06493900 3.95982700 -1.09890100
H 1.37766500 2.57425000 -1.04627700
C 4.40538700 4.17543600 -0.73637000
H 6.16782800 3.33254100 0.23245400
H 2.50369300 4.74189800 -1.63450700
H 4.90002100 5.12762300 -0.98609200

1G
Frequencies, energies and thermodynamic properties:
Lowest Vibrational Mode (cm^{-1}) = 14.0764
2nd Lowest Vibrational Mode (cm^{-1}) = 20.7664
E(RB-P86) (h) = -2332.26481
Thermal correction to Enthalpy (h) = 0.572181
Thermal correction to Gibbs Free Energy (h) = 0.455931
Total Entropy (cal/Kmol) = 244.667
E(RPBE1PBE) (h) = -2332.03990
Optimised cartesian coordinates (Å):
Ru 0.04787400 -0.07194100 -0.08483100
P -2.28793300 0.19607300 0.23946300
P 2.38469900 0.01547500 0.30821200
N -0.00158400 -0.41710000 2.10500900
O 0.13036100 0.94225200 -2.94399200
C -2.96013800 1.91119900 0.06776700
C 1.22874600 0.02133800 2.81137800
H 1.21108700 -0.36535400 3.85600300
H 1.23744200 1.13146700 2.86758700
C -3.34593300 2.69040800 1.17965200
H -3.27951700 2.28675600 2.20125900
C 3.57222400 -1.07970200 -0.58751400
C -3.51755500 -0.80784200 -0.70486500
C 3.64026200 2.39473300 1.31270100
H 3.53485300 1.98070800 2.32678300
C -4.84140500 -0.93666000 -0.22293600
H -5.14389000 -0.44930000 0.71750300
C 3.19912400 -1.71120400 -1.79238400
H 2.18900600 -1.55977400 -2.20189600
C 2.46958200 -0.50721700 2.09159100

H	2.44748900	-1.61768600	2.08491700
H	3.40730100	-0.18668900	2.58854800
C	-2.47029800	-0.30558900	2.01456100
H	-3.39194200	0.10029300	2.47893300
H	-2.52374000	-1.42327100	2.03480600
C	3.19625700	1.67229800	0.18434900
C	-1.21409300	0.12091900	2.77409500
H	-1.12371400	1.22721300	2.84012100
H	-1.26352400	-0.27096700	3.81610400
C	4.11446000	-2.53293900	-2.47544800
H	3.81264600	-3.02026300	-3.41578700
C	4.87695100	-1.27558700	-0.07624700
H	5.19332800	-0.77928100	0.85509900
C	-3.54574700	3.77069800	-1.40951200
H	-3.62356800	4.18773700	-2.42601600
C	-3.06331600	2.46455600	-1.22975500
H	-2.76792700	1.87177200	-2.11049200
C	0.09678500	0.52137000	-1.85320200
C	-3.16010500	-1.43085100	-1.91903000
H	-2.13403900	-1.33445600	-2.30529400
C	-3.82592400	4.00077000	0.99550800
H	-4.12394800	4.59736600	1.87221000
C	4.22952600	3.66341800	1.15537700
H	4.57137400	4.21641400	2.04452200
C	5.78429900	-2.10393200	-0.75617800
H	6.79535200	-2.25659700	-0.34675700
C	3.35097800	2.23995900	-1.10199100
H	3.00877500	1.69209100	-1.99481900
C	-3.92718500	4.54356100	-0.29639200
H	-4.30383200	5.56883800	-0.43789000
C	5.40489400	-2.73348800	-1.95693100
H	6.11942700	-3.38106300	-2.48915900
C	-5.78446100	-1.68876400	-0.94184900
H	-6.81041900	-1.79030800	-0.55419100
C	4.38296400	4.21988000	-0.12539900
H	4.84508500	5.21235300	-0.24560300
C	-4.11052300	-2.17571400	-2.64112000
H	-3.82022200	-2.65726200	-3.58809200
C	-5.42120400	-2.30857300	-2.15248300
H	-6.16345800	-2.89672600	-2.71494900
C	3.94307500	3.50356600	-1.25467500
H	4.06012300	3.93214100	-2.26253100
H	0.10233600	1.52612600	0.34677400
H	-0.01983200	-1.50585200	2.21820400
C	-0.02329800	-1.97529200	-0.52124300
O	-0.06419500	-3.08417900	-0.86590600
C	-0.81452500	-3.75348300	2.67600200
O	-2.01806500	-3.41924100	2.59861200
O	0.22870000	-3.03310000	2.50214900

H -0.58981500 -4.84144400 2.93662500

8. References

1. X. Liu and T. Werner, *Adv. Synth. Catal.*, 2021, **363**, 1096–1104.
2. Z. Han, L. Rong, J. Wu, L. Zhang, Z. Wang and K. Ding, *Angew. Chem., Int. Ed.*, 2012, **51**, 13041–13045.
3. C. Gunanathan and D. Milstein, *Angew. Chem., Int. Ed.*, 2008, **47**, 8661–8664.
4. G. M. Sheldrick, *Acta Crystallogr., Sect. A: Found. Adv.*, 2015, **71**, 3–8.
5. G. M. Sheldrick, *Acta Crystallogr., Sect. C: Struct. Chem.*, 2015, **71**, 3–8.
6. M. J. Frisch, G. W. Trucks, H. B. Schlegel, G. E. Scuseria, M. A. Robb, J. R. Cheeseman, G. Scalmani, V. Barone, G. A. Petersson, H. Nakatsuji, X. Li, M. Caricato, A. V. Marenich, J. Bloino, B. G. Janesko, R. Gomperts, B. Mennucci, H. P. Hratchian, J. V. Ortiz, A. F. Izmaylov, J. L. Sonnenberg, D. Williams-Young, F. Ding, F. Lipparini, F. Egidi, J. Goings, B. Peng, A. Petrone, T. Henderson, D. Ranasinghe, V. G. Zakrzewski, J. Gao, N. Rega, G. Zheng, W. Liang, M. Hada, M. Ehara, K. Toyota, R. Fukuda, J. Hasegawa, M. Ishida, T. Nakajima, Y. Honda, O. Kitao, H. Nakai, T. Vreven, K. Throssell, J. A. Montgomery Jr., J. E. Peralta, F. Ogliaro, M. J. Bearpark, J. J. Heyd, E. N. Brothers, K. N. Kudin, V. N. Staroverov, T. A. Keith, R. Kobayashi, J. Normand, K. Raghavachari, A. P. Rendell, J. C. Burant, S. S. Iyengar, J. Tomasi, M. Cossi, J. M. Millam, M. Klene, C. Adamo, R. Cammi, J. W. Ochterski, R. L. Martin, K. Morokuma, O. Farkas, J. B. Foresman and D. J. Fox, *Gaussian 16*, Revision C.01, Gaussian, Inc., Wallingford, CT, 2019.
7. C. N. Brodie, A. E. Owen, J. S. Kolb, M. Bühl and A. Kumar, *Angew. Chem., Int. Ed.*, 2023, **62**, e202306655.
8. E. Owen, A. Preiss, A. McLuskie, C. Gao, G. Peters, M. Bühl and A. Kumar, *ACS Catal.*, 2022, **12**, 6923–6933.
9. E. Owen, A. Preiss, A. McLuskie, C. Gao, G. Peters, M. Bühl and A. Kumar, *ACS Catal.*, 2023, **13**, 10796–10797.
10. L. Oates, A. S. Goodfellow, M. Bühl and M. Clarke, *Angew. Chem., Int. Ed.*, 2023, **62**, e202212479.
11. L. Oates, A. S. Goodfellow, M. Bühl and M. Clarke, *Green Chem.*, 2023, **25**, 3864–3868.
12. J. Luk, L. Andrew, G. Saini, M. J. Andrews, E. Feeke, A. P. McKay, D. B. Cordes, M. Bühl and A. Kumar, *ACS Catal.*, 2026, **16**, 2309–2317.
13. A. S. Goodfellow and M. Bühl, *Molecules*, 2021, **26**, 4072.
14. R. L. Martin, P. J. Hay and L. R. Pratt, *J. Phys. Chem. A*, 1998, **102**, 3565–3573.
15. Andrae, U. Haeussermann, M. Dolg, H. Stoll and H. Preuss, *Theor. Chim. Acta*, 1990, **77**, 123–141.

16. J. Tomasi, B. Mennucci and R. Cammi, *Chem. Rev.*, 2005, **105**, 2999–3093.
